# Aromaticity‐Engineered Open‐Shell Radical Anions for Air‐Stable Doublet Emission in *N*‐Annulated Perylene Diimide

**DOI:** 10.1002/advs.75858

**Published:** 2026-06-15

**Authors:** Jeongyoon Kim, Soyoon Lee, Min‐Ji Kim, Mina Ahn, Kyung‐Ryang Wee

**Affiliations:** ^1^ Department of Advanced Materials Chemistry and Division of Smart Energy Convergence Engineering Korea University Sejong Republic of Korea; ^2^ Center for Photovoltaic Materials Korea University Sejong Republic of Korea

**Keywords:** aromaticity, donor‐acceptor systems, luminescent radical ions, perylene diimides, through‐space charge‐transfer

## Abstract

We report the doublet emissive open‐shell radical anions, where the *N*‐annulated perylene diimide (PDIN–H) is functionalized with aromatic rings. This resulting [**PDI**–**MeR**]**^•−^** (R = BZ, benzene; NP, naphthalene; PY, pyrene) shows a high quantum yield, a long lifetime, and a high stability in air, as the ring size increases. The key to these properties is the combination of PDIN's aromaticity with predominant through‐space charge‐transfer (TSCT). This finding provides fundamental insights into unique open‐shell electronic structures and tunable optical functions, thereby paving the way for the development of novel doublet emitters and electroactive materials.

## Graphical abstract

Open‐shell type radical anions with stable doublet emission have been synthesized that feature aromaticity engineering, through‐space charge‐transfer (TSCT), and electro(fluoro)chromism.

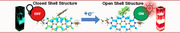

## 1 Introduction

Luminescent organic radicals represent powerful candidates for molecular quantum technology, including quantum sensing and information processing [1, 2], beyond their applications in organic electronics, especially in organic light‐emitting diodes (OLEDs) [3, 4]. Different from closed‐shell organic emitters, luminescent organic radicals are open‐shell molecules with one or more unpaired electrons in the outermost singly occupied molecular orbital (SOMO) [5], which generally exhibit short lifetimes and high reactivity [6]. This feature enables spin‐allowed doublet emission via transitions from the lowest‐energy doublet excited state (D_1_) to the doublet ground state (D_0_), without requiring intersystem crossing [7, 8, 9]. As a result, higher quantum efficiencies can be achieved because all recombination processes contribute to light emission without substantial nonradiative losses, outperforming closed‐shell organic emitters with singlet and triplet excited states [10].

Several different compounds were investigated as prominent light‐emitting radicals, usually falling into three categories according to the charge state of the chemical species (neutral, cationic, anionic) [11, 12, 13]. The majority of luminescent neutral π‐radicals are chlorinated triphenylmethyl (trityl) derivatives [14], such as tris(2,4,6‐trichlorophenyl)methyl (TTM) [15, 16, 17, 18] and perchlorotriphenylmethyl (PTM) [19, 20, 21, 22]; however, despite their stability, their photoluminescence quantum efficiencies (PLQEs) are marginal in solution [23, 24, 25]. Higher efficiencies can be achieved with constructing a donor–acceptor system that induces intramolecular charge transfer (CT) from donor to TTM in the excited state [26, 27, 28, 29, 30, 31, 32, 33]. In contrast, radical ions (both cationic and anionic) were considered unstable and essentially non‐emissive, or only weakly emissive, under ambient conditions, which have been major obstacles to the full investigation of doublet luminescence [34, 35, 36, 37, 38, 39].

However, ongoing research into rational molecular design has enabled the realization of long‐term stable and luminescent organic radical ions. A notable strategy is utilizing conformationally rigid *N*‐annulated perylene diimide (PDIN–H) [40, 41, 42]. The *N*‐annulated pyrrolic N–H site allows for an extra site for tunability, enabling fine control over optoelectronic properties, improving solubility, and lowering the electron affinity [43, 44]. Upon deprotonation of the N–H site, further substitution with flexible alkyl chains or bulky aryl groups effectively enforces planarity, thus preserving π‐conjugation even with bulky substituents present to stabilize the formed radical state [42]. Notably, the combination of PDIN and aromatic rings can promote efficient luminescence by suppressing aggregation and reducing structural flexibility [45]. Another is incorporating flexible alkyl chains to induce a through‐space charge‐transfer (TSCT) via the spatial separation of the donor and acceptor [46, 47, 48]. D–σ–A, formed by inserting sp^3^ hybridized carbon atoms into the core of the molecular structure, can regulate electronic coupling [49, 50]. This can enhance the efficiency of the charge transfer process, thereby improving the performance of luminescent materials [51, 52]. These strategies can weaken the electron‐vibrational coupling within the molecule, thereby reducing the rate of non‐radiative transitions and achieving the high PLQEs.

Hereby pendanting aromatic rings of varying sizes onto the PDIN–H, we employed TSCT characters to modulate the electronic structures and thus photophysical properties of organic radicals to yield a novel class of highly efficient open‐shell radical emitters, [**PDI**–**MeR**]**^•−^**. Progressive substitution from BZ to NP to PY extends the excited state character of [**PDI**–**MeR**]**^•−^** from local to TSCT in nature, leading to higher PLQEs and long‐lived doublet state emission. Notably, [**PDI**–**MePY**]**^•−^** shows remarkable stability of red doublet emission in air over several months. Along with the TSCT character, the enhanced delocalization and aromaticity within the PDIN highlight the promising potential as an electrochromic/electrofluorochromic material.

## 2 Results and Discussion

**FIGURE 1:**
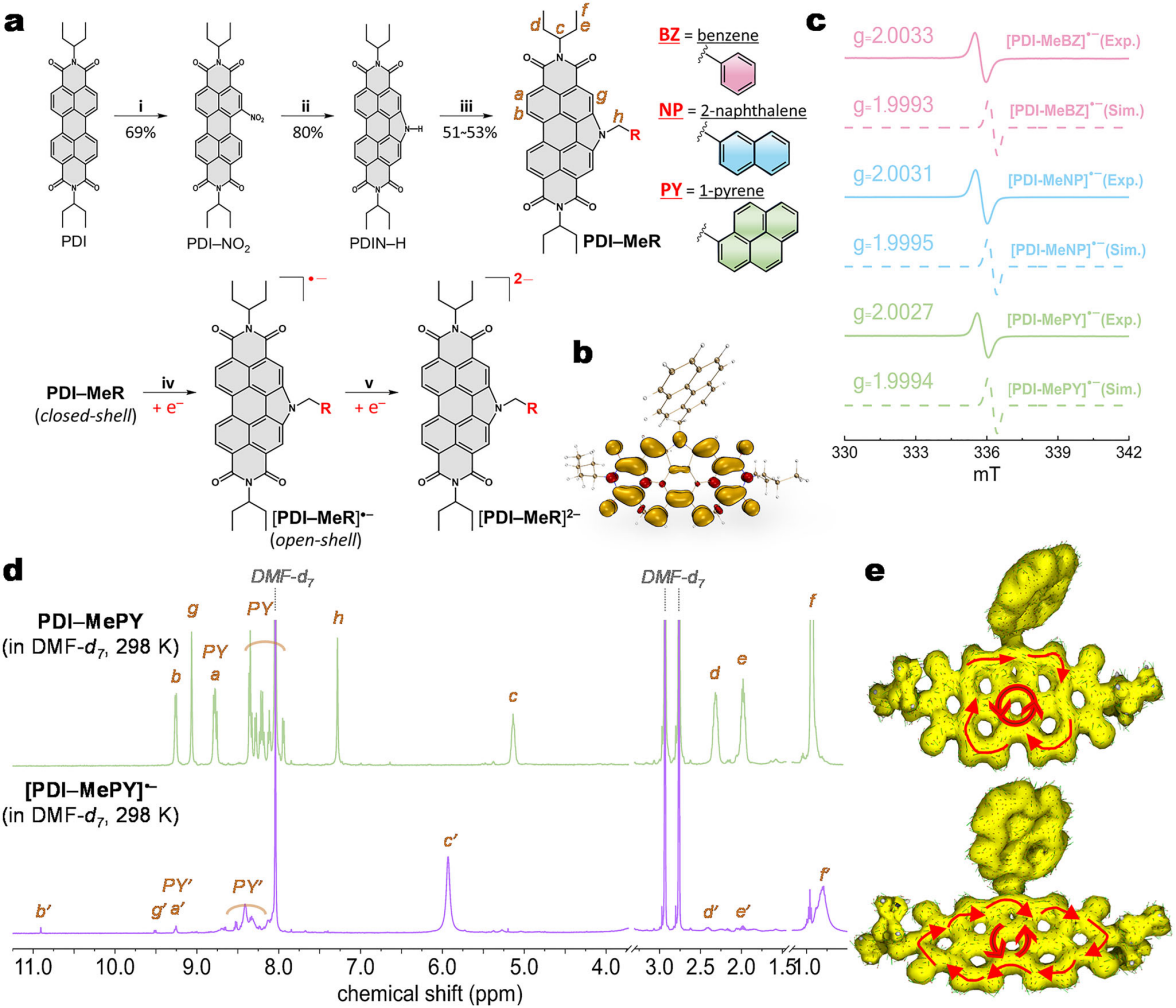
(a) Synthesis of closed‐shell **PDI**–**MeR** and open‐shell [**PDI**–**MeR**]**^•−^**. Reagents and conditions: (i) PDI, (NH_4_)_2_Ce(NO_3_)_6_, HNO_3_, H_2_SO_4_, DCM, rt, 15 h. (ii) PDI–NO_2_, PPh_3_, ODCB, 190°C, 3 h. (iii) PDIN–H, RCH_2_Br, K_2_CO_3_, Acetone, 65°C, 5 h. (iv) CoCp_2_ (5.0‐10.0 equiv), DMF, under Ar; or electrochemical reduction. (v) CoCp_2_ (1.0 equiv), DMF, under Ar; or electrochemical reduction. (b) Spin density map of [**PDI**–**MePY**]**^•−^**. (c) Experimental and simulated EPR spectra of [**PDI**–**MeR**]**^•−^** in DMF (298 K). (d) ^1^H NMR spectra of **PDI**–**MePY** and [**PDI**–**MePY**]**^•−^** in DMF‐d_7_ (298 K) with proton assignments (as labeled in Figure 1: *a*‐*h* for **PDI**–**MePY** and *a’*‐*g’* for [**PDI**–**MePY**]**^•−^**); peaks labeled PY correspond to pyrene protons. (e) AICD plots of **PDI**–**MePY** and [**PDI**–**MePY**]**^•−^** (B3LYP/6‐31G(d), isovalue = 0.05).

Figure 1a illustrates the synthetic route for PDI‐based luminescent organic radicals bearing three different aromatic pendant. PDIN–H was prepared by nitration of commercially available *N,N*‐Bis(ethylpropyl)perylene‐3,4,9,10‐tetracarboxylic diimide (PDI), followed by *N*‐annulation. Subsequent N‐alkylation of this key precursor using a base (K_2_CO_3_) afforded the target compounds **PDI**–**MeBZ**, **PDI**–**MeNP**, and **PDI**–**MePY** in ∼50% yield. Equimolar addition of the one‐electron reductant, cobaltocene (CoCp_2_), to a solution of PDI under an inert atmosphere fully converted to the neutral species into the corresponding radical anions. ICP‐OES analysis identified Co^3+^ as the counter cation of the open‐shell products, indicative of a doublet ground state for [**PDI**–**MeR**]^**•−**^ [CoCp_2_]^+^ (Figure S15).

Upon the reduction, the ^1^H NMR signals in the bay and ortho regions of PDI, as well as those from the aromatic substituents, were largely quenched, while some alkyl resonances in the imide region remained detectable (Figure 1d; Figure S5–S13). This is because the unpaired electrons accelerate nuclear spin relaxation processes, significantly broadening the ^1^H NMR signals of the open‐shell molecules [53]. In contrast, the resulting open‐shell [**PDI**–**MeR**]^**•−**^ exhibited clear EPR activity, showing a structureless one‐line signal centered at g value ≈ 2.003, characteristic of organic monoradicals (Figure 1c; Figure S21).

Calculated Spin density showed that the unpaired electron is mainly delocalized over the central N‐containing PDI core, whereas no density extends to the aromatic rings (Figure 1b; Figure S25). Such spin delocalization over an extended π‐systems dilutes the local spin density and reduces the reactivity, leading to enhanced overall stability. The anisotropy of the induced current density (AICD) plot possesses more extended global aromaticity in the radical anion than in the neutral state, characterized by a more dominant diatropic (clockwise) ring current flow along the outer rim, while a local paratropic (counterclockwise) current persists in the center (Figure 1e; Figure S26).

To illuminate the electronic properties of **PDI**–**MeR**, we first conducted cyclic voltammetry (CV) measurements (1 mm, 0.1 M TBAP in DCM, 100 mV s^−1^) (Figure S19 and Table S2). All compounds exhibit two reversible reduction waves at E_1/2_^(red1)^ = –0.73 to –0.76 V and E_1/2_^(red2)^ = –0.99 to –1.01 V (vs. SCE), separated by a small gap of ∼0.26 V. Relative to the parent PDIN–H (E_1/2_^(red1)^ = –1.28 V vs. Fc/Fc^+^) [54], the much less negative E_1/2_^(red1)^ indicates enhanced electron affinity of inner PDIN in **PDI**–**MeR**. Opting for DMF solution, the CV peaks merge into a single two‐electron reduction peak [55], as the solvent's high polarity and donor ability stabilize both the radical anions and the dianions (Figure S19 and Table S3). Repeated CV scan over 200 cycles demonstrated excellent electrochemical redox reversibility between **PDI**–**MeR** and [**PDI**–**MeR**]**^•−^** (Figure S20). Furthermore, the CV peaks of the [**PDI**–**MeR**]**^•−^** radical anion generated upon addition of CoCp_2_ reflect electron‐transfer interactions between **PDI**–**MeR** and CoCp_2_ (Figures S16 and S22) [56].

**FIGURE 2:**
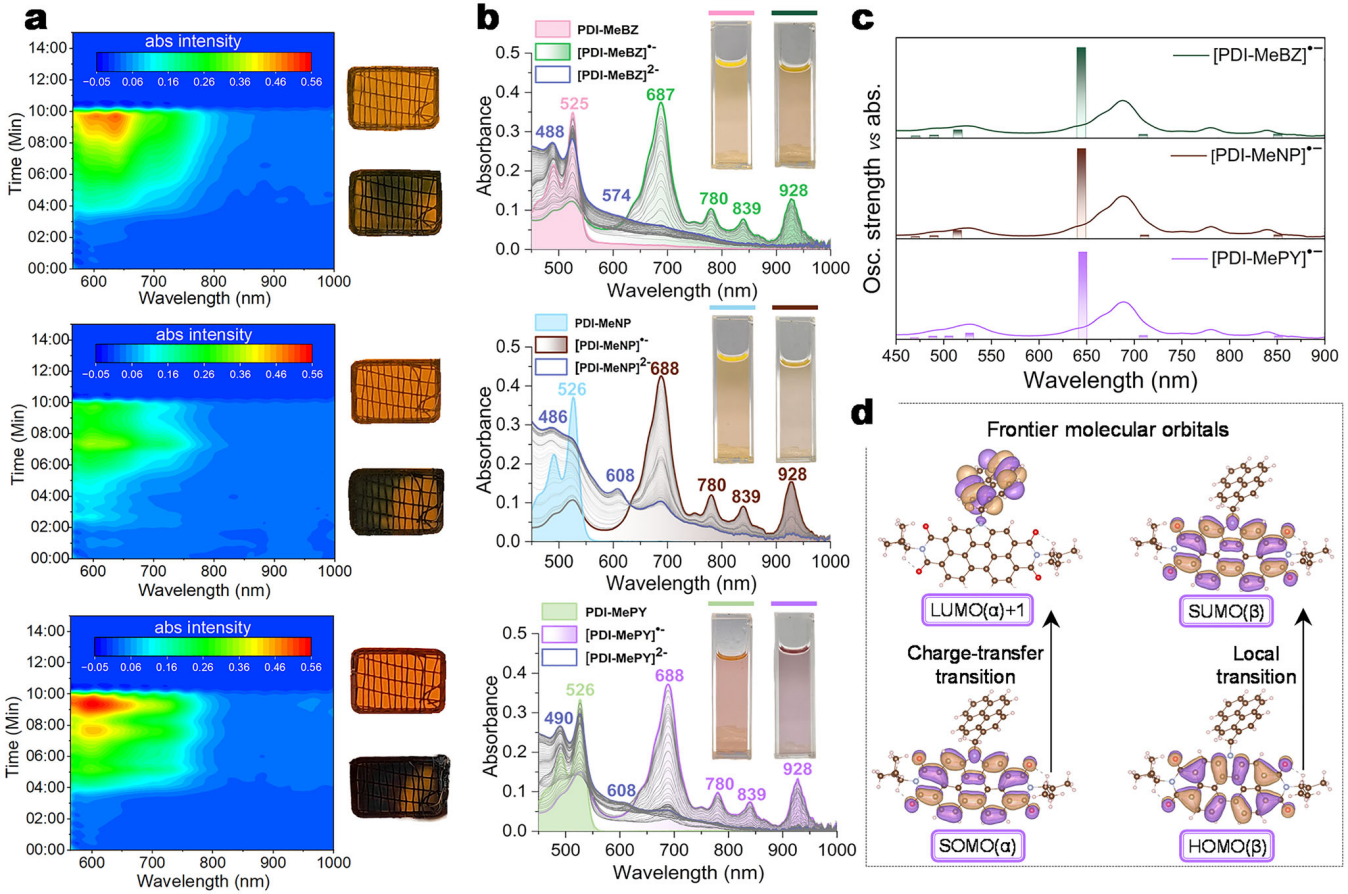
(a) Absorption map as a function of time and photographs of solution color changes around the Pt mesh electrode during spectroelectrochemical reduction of **PDI**–**MeR** (1 mm, 0.1 m TBAP in THF). (b) UV–vis–NIR absorption spectral changes of **PDI**–**MeR** (10 µm in DMF) during chemical reduction with CoCp_2_ and photographs of the neutral and radical anion solutions. (c) Experimental absorption spectra of [**PDI**–**MePY**]^**•−**^ in DMF at 298 K (lines) with TDDFT/PCM(DMF)‐B3LYP/6‐31+G(d) oscillator strengths (sticks). (d) Frontier molecular orbitals of [**PDI**–**MePY**]^**•−**^ involved in spin‐allowed D_0_ → D_1_ transition (oscillator strength (*f*) = 0.036).

In situ spectroelectrochemistry in solution examines a real‐time variation of absorption spectra, which was ascribed to electrochemical generation of radical anion [**PDI**–**MeR**]**^•−^**. Upon prolonged electrochemical bias, the absorption bands of **PDI**–**MeR** gradually decreased, while new peaks appeared at 600–800 nm, reaching maximum intensity within 8 min (Figure 2a; Figure S23). By chemical reduction with specific equivalents of CoCp_2_ (E = ‐1.3 V) [57, 58] in DMF, the radical anion (**^•−^**) and dianion (^2^ **^−^**) state could be selectively generated and clearly assigned (Figure 2b) [59]. Treating of **PDI**–**MeR** solution with 1 equivalents of CoCp_2_ afforded the monoanionic radical, which was air‐stable and exhibited distinct absorption spectral changes. By adding an excess of CoCp_2_, the generated radical anion was transformed into the dianion through the reduction of the formed radical anion. Time‐dependent DFT (TDDFT) calculations assign these absorptions of [**PDI**–**MeBZ**]**^•−^** and [**PDI**–**MeNP**]**^•−^** to the transitions at ∼850 nm (D_0_ → D_1_), ∼709 nm (D_0_ → D_2_), and ∼644 nm (D_0_ → D_3_) (Figure 2c; Table S11). For [**PDI**–**MePY**]**^•−^**, these absorptions are assigned to transitions at ∼850 nm (D_0_ → D_1_), ∼803 nm (D_0_ → D_2_, spin‐forbidden), ∼709 nm (D_0_ → D_3_), and ∼644 nm (D_0_ → D_4_) (Figure 2c; Table S11). Among these, the spin‐allowed D_0_ → D_1_ transition in [**PDI**–**MeBZ**]**^•−^** is confined to PDIN local excitation, whereas in [**PDI**–**MeNP**]**^•−^** and [**PDI**–**MePY**]**^•−^**, a combination of PDIN‐localized excitation and charge‐transfer excitation is involved. In particular, charge‐transfer excitation occurs from the PDIN (donor) to the NP/PY (acceptor), corresponding to SOMO(α) → LUMO(α) for [**PDI**–**MeNP**]**^•−^** and SOMO(α) → LUMO+1(α) for [**PDI**–**MePY**]**^•−^**, respectively (Figure 2d; Figure S27–S32).

**FIGURE 3:**
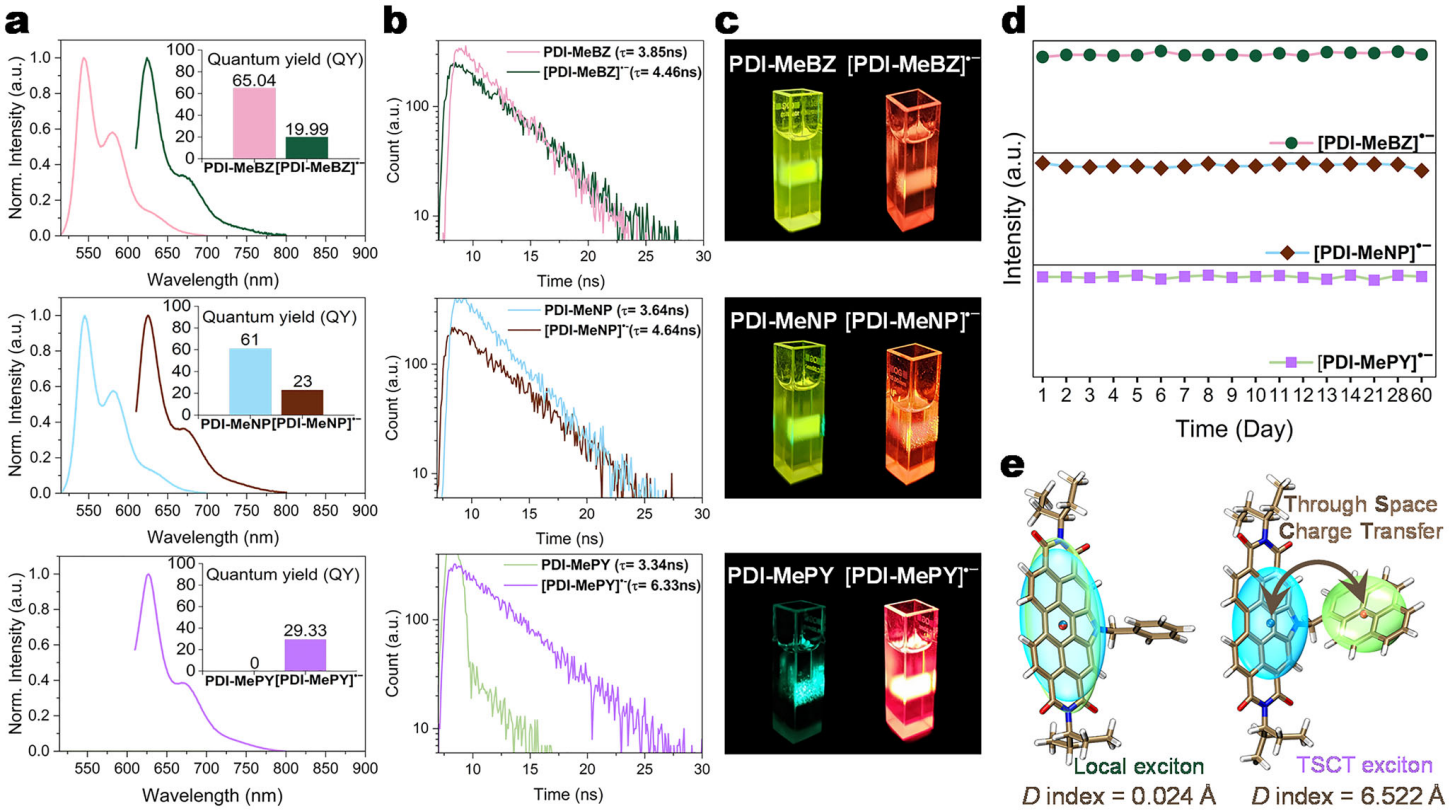
(a) Normalized PL spectra (lines) and PLQYs (inset) of **PDI**–**MeR** and [**PDI**–**MeR**]^**•−**^ (10 µm in DMF). (b) Emission lifetimes of **PDI**–**MeR** and [**PDI**–**MeR**]^**•−**^. (c) Emission photographs of **PDI**–**MeR** and [**PDI**–**MeR**]^**•−**^ under 520 and 610 nm laser excitation, respectively. (d) Doublet emission stability of [**PDI**–**MePY**]^**•−**^ over Day 1–Day 60, evaluated from PL intensity at 625 nm. (e) Hole (blue) and electron (lime) distributions in the D_2_ excited states of [**PDI**–**MeBZ**]^**•−**^ and [**PDI**–**MePY**]^**•−**^ with centroid separation (*D* index).

Closed‐shell **PDI**–**MeBZ** and **PDI**–**MeNP** exhibited intense emission over 520–700 nm with PLQEs of ca. 60% and relatively small Stokes shift (Figure S17), whereas **PDI**–**MePY** was non‐emissive. This **PDI**–**MeR** emission was insensitive to solvent polarity (Figure S18), consistent with a locally excited (LE) state. Open‐shell [**PDI**–**MeR**]^**•−**^ showed efficient doublet photoluminescence (PL) in DMF, featuring redshifted emission (625–800 nm) and progressively enhanced PLQEs (ca. 20%–29%) with larger aromatic pendant size (Figure 3a). In addition, emission lifetimes were collected by time‐correlated single photon counting (TCSPC) measurements, which revealed excited state lifetime of ca. 4–6 ns ([**PDI**–**MeBZ**]^**•−**^ (τ = 4.46 ns), [**PDI**–**MeNP**]^**•−**^ (τ = 4.64 ns), and [**PDI**–**MePY**]^**•−**^ (τ = 6.33 ns)) (Figure 3b). Notably, reduction of **PDI**–**MePY** leads to increased PLQE and decreased non‐radiative decay rate, despite the smaller bandgap for the radical anionic state relative to the neutral state. Altogether, we find k_r_’s of the same order of magnitude for all three radical ion series, whereas a slight decrease of k_nr_ is observed for the [**PDI**–**MePY**]^**•−**^ relative to the others (Table S1). Such red doublet emission from [**PDI**–**MeR**]^**•−**^ showed excellent stability with the almost unchanged PL intensity for several months (Figure 3c,d; Figure S24). Figure 3e reveals hole–electron overlap in [**PDI**–**MeBZ**]^**•−**^ (D = 0.024 Å) and separation in [**PDI**–**MePY**]^**•−**^ (D = 6.522 Å) in the D_2_ state, followed by recombination in the D_1_ state (Figure S33).

Overall, the emission of **PDI**–**MeR** and its radical anion ([**PDI**–**MeR**]^**•−**^) mainly occurs from the lower‐energy excited states, localized on the PDIN moiety, in accordance with Kasha's rule [60]. The aromatic pendant plays a dual role, acting as an electron donor in **PDI**–**MeR** and as an electron acceptor in [**PDI**–**MeR**]^**•−**^, thereby modulating PDIN‐localized emission. In the **PDI**–**MeR**, the TSCT between aryl donor and PDIN acceptor results in the gradual PDIN emission quenching (**PDI**–**MeBZ** > **PDI**–**MeNP** > **PDI**–**MePY**). In contrast, in the [**PDI**–**MeR**]^**•−**^, as the LE excited state extends beyond its localized character to form the TSCT excited state, the charge transfer between PDIN donor and aryl acceptor results in the opposite trend, with PDIN emission gradually enhancing from [**PDI**–**MeBZ**]^**•−**^ to [**PDI**–**MeNP**]^**•−**^ to [**PDI**–**MePY**]^**•−**^.

**FIGURE 4:**
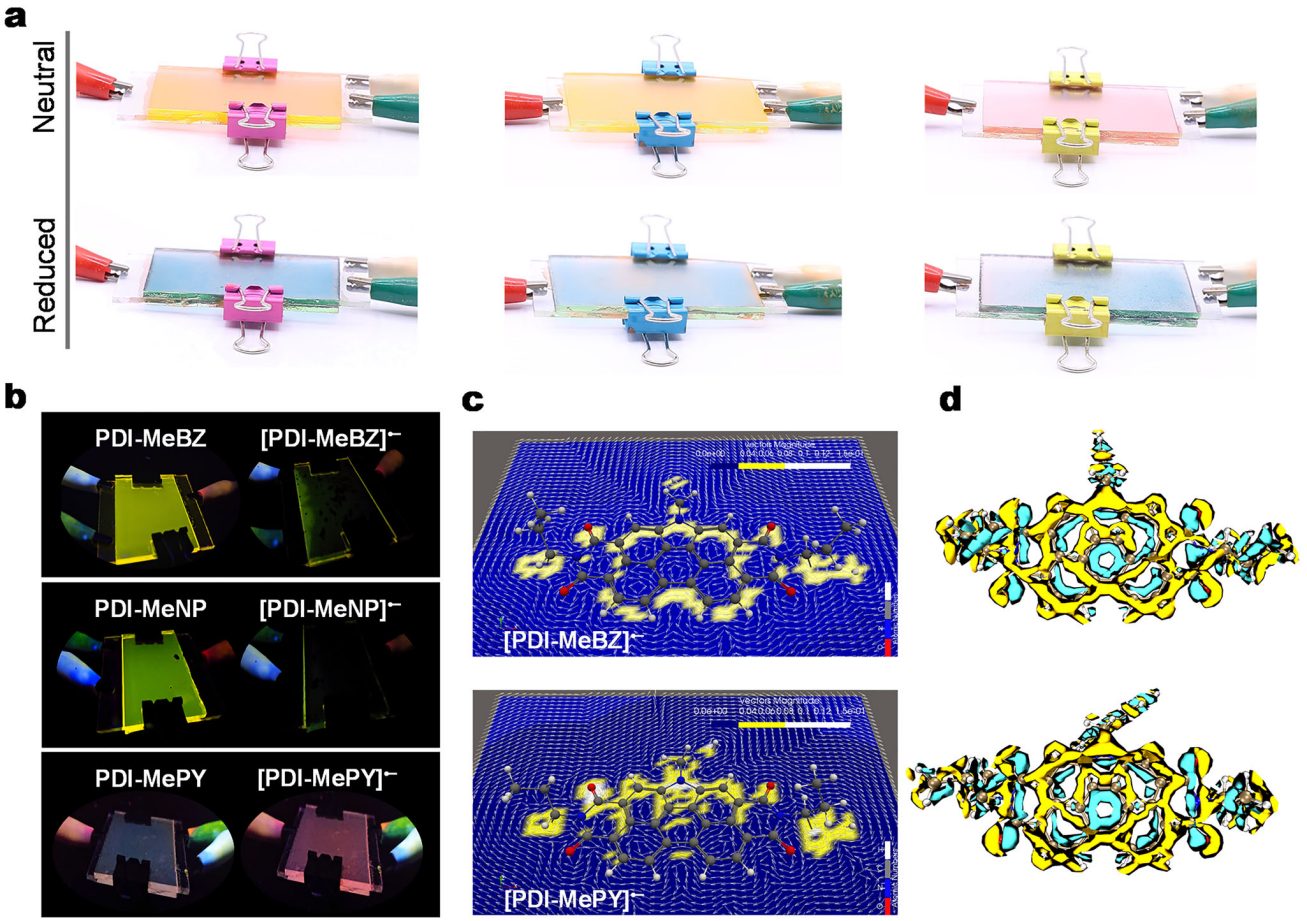
(a) Electrochromic device: initial state (top) and reduced state (‐1.6 V, bottom); left to right: **PDI**–**MeBZ**, **PDI**–**MeNP**, and **PDI**–**MePY**. (b) Electrofluorochromic device: initial state (left) and reduced state (‐1.6 V, right). (c) MICD visualization using streamlines and ring‐current flow direction in a plane placed 1.5 bohr above the molecular plane. (d) Signed MICD maps in [**PDI**–**MeBZ**]^**•−**^ and [**PDI**–**MePY**]^**•−**^ (diatropic, yellow; paratropic, cyan).

To demonstrate the feasibility of the electroactive **PDI**–**MeR** as electrochromic/electrofluorochromic materials, a proof‐of‐concept device was fabricated. This simple device consists of two FTO‐coated glass substrates with a DMF solution of the respective compound containing an electrolyte introduced between them. Applying ‐1.6 V between the two glass electrodes induces a gradual color change from orange red to green (Figure 4a; Movie S1–S3), characteristic of the radical species, accompanied by a clear change in electrofluorescence under UV irradiation (Figure 4b; Movie S4–S6). For the **PDI**–**MeBZ** and **PDI**–**MeNP**, the device emits light‐green fluorescence in the neutral state, which is quenched upon reduction. In contrast, the **PDI**–**MePY**‐based device is non‐emissive in the neutral state, but turns on red‐light emission in the reduced state. The initial color and emission of the devices are restored via oxidation induced by air diffusion into the cell.

To quantify the relative aromaticity of the [**PDI**–**MeR**]^**•−**^ with the PDI core aligned parallel to the XY plane and to elucidate the origin of its intrinsic stability, gauge‐including magnetically induced current (GIMIC) was analyzed. The signed magnetically induced current density (MICD) map illustrates the coexistence of diatropic and paratropic ring‐currents throughout PDIN rings (Figure 4d). Additionally, the current flow around the bay‐fused ring of the PDI core, particularly near the *N*‐annulated substitutional sites, increases from [**PDI**–**MeBZ**]^**•−**^ to [**PDI**–**MePY**]^**•−**^ (Figure 4c). Quantitatively, the net ring‐current densities of the PDI core are at +10.2 nA/T in [**PDI**–**MeBZ**]^**•−**^ and ‐10.1 nA/T in [**PDI**–**MePY**]^**•−**^, further supporting the stronger aromaticity in [**PDI**–**MePY**]^**•−**^. (Figure S34 and Table S18–S20).

## 3 Conclusion

In conclusion, we introduce a new of stable PDI‐based radical anions, [**PDI**–**MeR**]**^•−^**, as efficient doublet emitters, distinct from the conventional triphenylmethyl radicals. Combined experimental and theoretical studies demonstrate that TSCT between the PDIN and aromatic pendants enhances doublet photoluminescence quantum yield, lifetime, and stability. These findings establish a strategy for stabilizing emissive open‐shell ionic states and enabling bright doublet emission, expanding the scope of radical‐based optoelectronic materials.

## Funding

The National Research Foundation of Korea (NRF) grant funded by the Korea government (MSIT) (NRF‐2023R1A2C1005399 and RS‐2026‐25519439)

## Conflicts of Interest

The authors declare no conflicts of interest.

## Supporting information




**Supporting File 1**: advs75858‐sup‐0001‐SuppMat.docx.

**Supporting File 2**: advs75858‐sup‐0002‐MovieS1.mp4.

**Supporting File 3**: advs75858‐sup‐0003‐MovieS2.mp4.

**Supporting File 4**: advs75858‐sup‐0004‐MovieS3.mp4.

**Supporting File 5**: advs75858‐sup‐0005‐MovieS4.mp4.

**Supporting File 6**: advs75858‐sup‐0006‐MovieS5.mp4.

**Supporting File 7**: advs75858‐sup‐0007‐MovieS6.mp4.

## Data Availability

The data that support the findings of this study are available from the corresponding author upon reasonable request.
