# Supplementary material for: Aromaticity‐Engineered Open‐Shell Radical Anions for Air‐Stable Doublet Emission in N‐Annulated Perylene Diimide
Source: Adv Sci (Weinh). 2026 Jun 15;13(47):e75858. doi: 10.1002/advs.75858 (PMC13335971; doi:10.1002/advs.75858)
Supplement: Supplementary file 1 [file ADVS-13-e75858-s003.docx]

Supporting Information

Aromaticity-Engineered Open-Shell Radical Anions for Air-Stable Doublet Emission in *N*-Annulated Perylene Diimide

Jeongyoon Kim, Soyoon Lee, Min-Ji Kim, Mina Ahn* and Kyung-Ryang Wee*

| Contents | | Page |
| --- | --- | --- |
| General Methods | | S3–S4 |
| Synthetic procedures and compound data | | S5–S7 |
| Figure S1–S13. ^1^H-NMR and ^13^C{^1^H}-NMR spectra of **PDI**–**NO_2_**, **PDIN**–**H**,  **PDI**–**MeR**, [**PDI**–**MeR**]**^•−^** | | S8–S14 |
| Figure S14. GC-MS data of **PDI**–**MeR** | | S15 |
| Figure S15. ICP–OES spectra of **PDI**–**MeR** with characteristic cobalt emission lines | | S16 |
| Figure S16. Cyclic voltammogram for the oxidation and reduction of **Cobaltocene** | | S17 |
| Table S1. Photophysical parameters of synthesized **PDI**–**MeR** and [**PDI–MeR**]^•−^ | | S18 |
| Table S2. CV data for the observed reduction wave of **PDI**–**MeR** in DCM | | S18 |
| Table S3. CV data for the observed reduction wave of **PDI**–**MeR** in DMF | | S18 |
| Figure S17. UV-vis absorption/ fluorescence spectra of **PDI**–**MeR** in DMF | | S19 |
| Figure S18. UV-vis absorption/ fluorescence spectra of **PDI**–**MeR** in solvents | | S20 |
| Figure S19. Cyclic voltammogram for the oxidation and reduction of **PDI**–**MeR** | | S21 |
| Figure S20. Cyclic voltammogram for the reduction of **PDI**–**MeR** over 200 cycles | | S22 |
| Figure S21. CW EPR spectra of [**PDI**–**MeR**]**^•−^** in DMF | | S23 |
| Figure S22. CV comparison of **PDI**–**MeR** and [**PDI**–**MeR**]**^•−^** in DMF | | S24 |
| Figure S23. UV-vis absorption spectral change of **PDI**–**MeR** to [**PDI**–**MeR**]**^•−^** | | S25 |
| Figure S24. Doublet emission stability of chemically generated [**PDI**–**MeR**]**^•−^** | | S26 |
| Calculation Details | S27 | |
| Figure S25. Spin density maps of [**PDI**–**MeR**]**^•−^** radical anion states | S28 | |
| Figure S26. AICDplots of **PDI**–**MeR** and [**PDI**–**MeR**]**^•−^ (**R = BZ, NP) | S29 | |
| Figure S27-S32. Energy levels and electronic transitions | S30–S35 | |
| Table S4. Cartesian coordinates for optimized structure for **PDI**–**MeBZ** | S36 | |
| Table S5. Cartesian coordinates for optimized structure for [**PDI**–**MeBZ**]**^•−^** | S37 | |
| Table S6. Cartesian coordinates for optimized structure for **PDI**–**MeNP** | S38 | |
| Table S7. Cartesian coordinates for optimized structure for [**PDI**–**MeNP**]**^•−^** | S39 | |
| Table S8. Cartesian coordinates for optimized structure for **PDI**–**MePY** | S40 | |
| Table S9. Cartesian coordinates for optimized structure for [**PDI**–**MePY**]**^•−^** | S41 | |
| Table S10. TDDFT values of the five lowest energy transition of **PDI**–**MeR** | S42 | |
| Table S11. TDDFT values of the five lowest energy transition of [**PDI**–**MeR**]**^•−^** | S43 | |
| Table S12. Excited states calculated by TDDFT Calculations of **PDI**–**MeBZ** | S44–S45 | |
| Table S13. Excited states calculated by TDDFT Calculations of **PDI**–**MeNP** | S46–S47 | |
| Table S14. Excited states calculated by TDDFT Calculations of **PDI**–**MePY** | S48–S49 | |
| Table S15. Excited states calculated by TDDFT Calculations of [**PDI**–**MeBZ**]**^•−^** | S50–S54 | |
| Table S16. Excited states calculated by TDDFT Calculations of [**PDI**–**MeNP**]**^•−^** | S55–S58 | |
| Table S17. Excited states calculated by TDDFT Calculations of [**PDI**–**MePY**]**^•−^** | S59–S63 | |
| Figure S33. Excited state electron and hole distributions of [**PDI**–**MeR**]**^•−^** | S64 | |
| Figure S34. Current strength susceptibilities and pathways for [**PDI–MeR**]**^•−^** | S65 | |
| Table S18–S20. The induced current strengths of selected bonds in [**PDI–MeR**]**^•−^** | S65–S66 | |
| References | S67 | |

**General Methods**

Based on standard Schlenk techniques, all of the synthesis experimental procedures were performed under a dry argon condition. Reagents and solvents were purchased from commercial sources and used as received without further purification, unless otherwise stated. All steps of synthesis were monitored using thin layer chromatography (TLC) on silica gel-precoated glass plates. Flash column chromatography was performed with silica gel 60 G (230–400 mesh). The synthesized compounds were characterized by ^1^H-NMR or ^13^C{^1^H}-NMR, and elemental analysis. 1H and proton decoupled 13C spectra were measured using a Bruker500 spectrometer operating at 500 MHz and 125 MHz, respectively, and all proton and carbon chemical shifts were measured relative to the internal residual chloroform (99.5% CDCl_3_) or *N,N*-Dimethylformamide (99.5% DMF-d_7_) in the locking solvent. The GC-MS analysis was performed using a highly sensitive Gas Chromatograph/Mass Selective Detector spectrometer (Agilent, 7890B-5977B GC/MSD). The Radical analyses were performed using an electron spin resonance (ESR) spectrometer (Bruker, EMXplus-9.5/2.7). The presence of Co from cobaltocene was confirmed using an inductively coupled plasma–optical emission spectrometer (ICP-OES) (PerkinElmer Avio 500 & Avio 550). The **PDI**–**NO_2_** and **PDIN**–**H** were prepared based on the previously published method. ^[1, 2]^

**Spectroscopic measurements**: The UV-vis absorption spectra were recorded using a Scinco Mega-2100 spectrophotometer in dual beam mode, and the fluorescence emission measurements were carried out using Shimadzu fluorometer (RF-6000) with a wavelength resolution of ~1 nm. Fluorescence lifetimes were measured using a time-resolved photoluminescence spectrometer (Edinburgh Instruments, FLS-1000) equipped with a time-correlated single photon counting (TCSPC) module. A pulsed diode laser was used as the excitation source, and the repetition rate was adjusted to avoid pulse pile-up effects. The excitation wavelength was set to 520 nm for **PDI**–**MeR** and 610 nm for [**PDI**–**MeR**]**^•−^**. The instrument response function (IRF) was measured separately and used for iterative reconvolution fitting of the decay curves. The emission quantum yields (Φ_PL_) were determined using an integrating sphere module equipped in the FLS-1000 system, enabling absolute quantum yield measurements under identical experimental conditions.

**Cyclic Voltammetry (CV)**: A CH Instruments 701D potentiate was used for electrochemical measurements, and cyclic voltammetry (CV) was performed in an electrolytic solution containing the electroactive compounds (1 mM) and tetra-*n*-butylammonium perchlorate (0.1 M TBAP) in deoxygenated DCM and DMF. A three-electrode configuration, glassy carbon, platinum wire, and SCE, were used as working, counter, and reference electrodes, respectively.^[3, 4]^ The scanning rate was 50 mV s^−1^.

**Spectroelectrochemical measurements**: SEC Measurements were carried out using a custom-made, optically transparent, thin-layer electrochemical cell (light pass length = 1 mm) equipped with a platinum mesh working electrode and a platinum coil counter electrode, and a SCE was used as the reference electrode.^[4, 5]^ Three electrodes were placed into the **PDI**–**MeR** solution with 0.1 M TBAP. Potentials were applied using a commercial electrochemical analyzer (CH Instruments 701D potentiostat). The electrochemical analyzer controlled the potential of the working electrode, and a Scinco Nano-MD UV-vis spectrophotometer obtained the absorption spectra of the redox species. SEC absorption spectra was recorded at ~10 sec intervals for 1200 sec to monitor the potential induced spectral evolution.

**Device Fabrication**: Electrochromic and electrofluorochromic devices^[6]^ were fabricated in a sandwich configuration using Fluorine-doped Tin Oxide (FTO)-coated glass substrates (300 × 300 × 2.2 mm, 6 ~ 9 Ω/sq). The device architecture was FTO-coated glass / DMF solution (1 mM **PDI**–**MeR**, 0.1 M TBAP) / FTO-coated glass. All devices were fabricated and measured in air. An external potential of -1.6 V was applied using CH Instruments 701D potentiostat to evaluate the colorimetric and electrofluorochromic performance.

**Calculation Summary**: Geometry optimizations, TD-DFT calculations, electron-hole and spin density analyses, anisotropy of the induced current density (AICD) calculations, and gauge-including magnetically induced current (GIMIC) calculations were performed. More details are provided in the Calculation Details section. **Synthetic procedures and compound data**

*Synthesis of* ***PDI****–****NO_2_*** : A mixture of 2,9-di(pentan-3-yl)anthra[2,1,9-def:6,5,10-d'e'f']diisoquinoline-1,3,8,10(2H,9H)-tetraone (0.5 g, 0.38 mmol), Ammonium Cerium(IV) Nitrate (1 g, 0.76 mmol) HNO_3_ (0.4 mL, 3.5 mmol), H_2_SO_4_ (0.5 mL, 3.94 mmol) in Dichloromethane (50 mL) was refluxed under argon at RT for 15 h. deionized water (50 mL) was poured, and organic layer was separated using a separating funnel. The water layer was washed using DCM (×3) for the extracted remained organic residue. After combined all of the organic solvents, the organic layer was dried over anhydrous MgSO_4_, and then filtered off. The solvent was removed under reduced pressure, and the residue was purified by silica gel column chromatography using DCM/petroleum ether mixture eluent.

*5-nitro-2,9-di(pentan-3-yl)anthra[2,1,9-def:6,5,10-d'e'f']diisoquinoline-1,3,8,10(2H,9H)-tetraone* ***(PDI****–****NO_2_)***. maroon powder (0.374 g, Yield: 69%). ^1^H-NMR (500 MHz, CDCl_3_, ppm): δ= 8.80 (d, J = 8.0 Hz, 1H), 8.74–8.70 (m, 4H), 8.60 (d, J = 8.5 Hz, 1H), 8.26 (d, J = 8.5 Hz, 1H), 5.08–5.02 (m, 2H), 2.29–2.20 (m, 4H), 1.98–1.89 (m, 4H), 0.92 (t, 12H, J = 7.5 Hz). ^13^C{^1^H}-NMR (125 MHz, CDCl_3_, ppm): δ= 147.73, 135.52, 132.97, 129.54, 129.41, 129.16, 127.97, 127.56, 126.72, 126.50, 124.51, 124.05, 58.24, 57.95, 24.99, 24.91, 11.31, 11.28.

*Synthesis of* ***PDIN****–****H*** : A mixture of 5-nitro-2,9-di(pentan-3-yl)anthra[2,1,9-def:6,5,10-d'e'f']diisoquinoline-1,3,8,10(2H,9H)-tetraone (PDI–NO_2_, 0.2 g, 0.35 mmol), Triphenylphosphane (0.23 g, 0.87 mmol) in *o*-Dichlorobenzene (3 mL) was refluxed under argon at 190 ℃ for 3 h. After completing the reaction, remove the solvent. and the residue was purified by silica gel column chromatography using DCM/Acetone mixture eluent.

*2,8-di(pentan-3-yl)-1H-pyrido[3',4',5':4,5]naphtho[2,1,8-cde]pyrido[3',4',5':4,5]naphtho[8,1,2-ghi]isoindole-1,3,7,9(2H,5H,8H)-tetraon* ***(PDIN****–****H)****.* (DCM/Acetone = 20:1). Dark red powder (0.15 g, Yield: 80%). ^1^H-NMR (500 MHz, CDCl_3_, ppm): δ= 9.86 (s, 1H), 9.06 (s, 2H), 9.00 (d, *J* = 8.0 Hz, 2H), 8.88 (d, *J* = 8.0 Hz, 2H), 5.25–5.19 (m, 2H), 2.39–2.32 (m, 4H), 2.04–1.99 (m, 4H), 0.98 (t, *J* = 7.5 Hz, 12H).
^13^C{^1^H}-NMR (125 MHz, CDCl_3_, ppm): δ= 133.68, 133.29, 125.05, 124.17, 122.17, 57.81, 25.25, 11.46.

*Synthesis of* ***PDI****–****MeBZ*** : A mixture of 2,8-di(pentan-3-yl)-1H-pyrido[3',4',5':4,5]naphtho[2,1,8-cde]pyrido[3',4',5':4,5]naphtho[8,1,2-ghi]isoindole-1,3,7,9(2H,5H,8H)-tetraon (PDIN–H, 0.1 g, 0.183 mmol), Benzyl bromide (0.044 mL, 0.368 mmol), K_2_CO_3_ (0.102 g, 0.736 mmol) in Acetone (15 mL) was refluxed under argon at 65 °C for 4 h.
After cooling to RT, deionized water (50 mL) was poured, and organic layer was separated using a separating funnel. The water layer was washed using DCM (×3) for the extracted remained organic residue. After combined all of the organic solvents, the organic layer was dried over anhydrous MgSO_4_, and then filtered off. The solvent was removed under reduced pressure, and the residue was purified by silica gel column chromatography using DCM/*n*-Hexane mixture eluent.

*5-benzyl-2,8-di(pentan-3-yl)-1H-pyrido[3',4',5':4,5]naphtho[2,1,8-cde]pyrido[3',4',5':4,5]naphtho[8,1,2-ghi]isoindole-1,3,7,9(2H,5H,8H)-tetraone* ***(PDI****–****MeBZ)****.* (DCM/*n*-Hexane = 4:1). Red powder (0.06 g, Yield: 53%). ^1^H-NMR (500 MHz, DMF-d_7_, ppm): δ= 9.15 (d, *J* = 8.0 Hz, 2H), 9.06 (s, 2H), 8.73 (d, *J* = 8.0 Hz, 2H), 7.57 (d, *J* = 7.0 Hz, 2H), 7.44−7.41 (m, 2H), 7.37−7.34 (m, 1H), 6.45 (s, 2H), 5.21−5.15 (m, 2H), 2.42−2.33 (m, 4H), 2.08−1.99 (m, 4H), 1.00 (t, *J* = 7.5 Hz, 12H). ^13^C{^1^H}-NMR (125 MHz, CDCl_3_, ppm): δ= 136.17, 135.11, 132.91, 129.35, 128.68, 127.10, 124.89, 124.06, 121.99, 119.93, 57.81, 50.44, 25.24, 11.48. GC-MS (*m/z*) calcd. for C_41_H_35_N_3_O_4_: 633.26; found: 633.4 [M]^+^.

*Synthesis of* ***PDI****–****MeNP*** : A mixture of *2,8-di(pentan-3-yl)-1H-pyrido[3',4',5':4,5]naphtho[2,1,8-cde]pyrido[3',4',5':4,5]naphtho[8,1,2-ghi]isoindole-1,3,7,9(2H,5H,8H)-tetraon* (PDIN–H, 0.1g, 0.183 mmol), 2-(Bromomethyl)naphthalene (0.081 g, 0.368 mmol),
K_2_CO_3_ (0.102 g, 0.736 mmol) in Acetone (15 mL) was refluxed under argon at 65 °C for 4 h. After cooling to RT, deionized water (50 mL) was poured, and organic layer was separated using a separating funnel. The water layer was washed using DCM (×3) for the extracted remained organic residue. After combined all of the organic solvents, the organic layer was dried over anhydrous MgSO_4_, and then filtered off. The solvent was removed under reduced pressure, and the residue was purified by silica gel column chromatography using DCM/*n*-Hexane mixture eluent.

*5-(naphthalen-2-ylmethyl)-2,8-di(pentan-3-yl)-1H-pyrido[3',4',5':4,5]naphtho[2,1,8-cde]pyrido[3',4',5':4,5]naphtho[8,1,2-ghi]isoindole-1,3,7,9(2H,5H,8H)-tetraone*. ***(PDI****–****MeNP)*** (DCM/*n*-Hexane = 4:1). Red powder (0.064 g, Yield: 51%). ^1^H-NMR (500 MHz, DMF-d_7_, ppm): δ= 9.22 (d, *J* = 8.0 Hz, 2H), 9.14 (s, 2H), 8.77 (d, *J* = 8.0 Hz, 2H), 8.13 (s, 1H), 7.96 (d, *J* = 8.5 Hz, 1H), 7.93−7.88 (m, 2H), 7.65 (dd, *J* = 8.5, 1.5 Hz, 1H), 7.53−7.51 (m, 2H), 6.66 (s, 2H), 5.20−5.14 (m, 2H), 2.39−2.33 (m, 4H) 2.06−1.98 (m, 4H), 0.98 (t, *J* = 7.5 Hz, 12H). ^13^C{^1^H}-NMR (125 MHz, CDCl_3_, ppm): δ= 135.19, 133.67, 133.31, 133.14, 132.95, 129.40, 127.96, 127.80, 126.77, 126.59, 126.00, 124.93, 124.46, 124.09, 122.04, 120.00, 57.81, 50.60, 31.60, 25.23, 22.66, 14.12, 11.47. GC-MS (*m/z*) calcd. for C_45_H_37_N_3_O_4_: 683.28; found: 683.4 [M]^+^.

*Synthesis of* ***PDI****–****MePY***: A mixture of *2,8-di(pentan-3-yl)-1H-pyrido[3',4',5':4,5]naphtho[2,1,8-cde]pyrido[3',4',5':4,5]naphtho[8,1,2-ghi]isoindole-1,3,7,9(2H,5H,8H)-tetraon* (PDIN–H, 0.1g, 0.183 mmol), 1-(Bromomethyl)pyrene (0.081 g, 0.276 mmol), K_2_CO_3_ (0.076g, 0.552 mmol) in Acetone (15 mL) was refluxed under argon at 65°C for 4 h. After cooling to RT, deionized water (50 mL) was poured, and organic layer was separated using a separating funnel. The water layer was washed using DCM (×3) for the extracted remained organic residue. After combined all of the organic solvents, the organic layer was dried over anhydrous MgSO_4_, and then filtered off. The solvent was removed under reduced pressure, and the residue was purified by silica gel column chromatography using DCM/*n*-Hexane mixture eluent.

*2,8-di(pentan-3-yl)-5-(pyren-1-ylmethyl)-1H-pyrido[3',4',5':4,5]naphtho[2,1,8-cde]pyrido[3',4',5':4,5]naphtho[8,1,2-ghi]isoindole-1,3,7,9(2H,5H,8H)-tetraone* ***(PDI****–****MePY)*** (DCM/*n*-Hexane = 4:1). Orange powder (0.074 g, Yield: 53%). ^1^H-NMR (500 MHz, DMF-d_7_, ppm): δ= 9.26 (d, *J* = 8.0 Hz, 2H), 9.06 (s, 2H), 8.80–8.75 (m, 3H), 8.36–8.33 (m, 3H), 8.28 (d, *J* = 8.0 Hz, 1H), 8.23–8.18 (m, 2H), 8.12 (t, *J* = 7.5 Hz, 1H), 7.94 (d, *J* = 7.5 Hz, 1H), 7.28 (s, 2H), 5.14 (s, 2H), 2.33–2.30 (m, 4H), 2.01–1.95 (m, 4H), 0.94 (t, *J* = 7.5Hz, 12H). ^13^C{^1^H}-NMR (125 MHz, CDCl_3_, ppm): δ= 135.42, 132.92, 131.78, 131.18, 130.46, 129.04, 128.57, 128.40, 127.96, 127.31, 126.27, 125.88, 125.68 125.06, 124.89, 124.50, 124.05, 122.03, 121.27, 120.05, 57.76, 48.85, 25.19, 11.46. GC-MS (*m/z*) calcd. for C_51_H_39_N_3_O_4_: 757.31; found: 757.4 [M]^+^.

Figure S1. ^1^H-NMR spectrum of **PDI**–**NO_2_** in CDCl_3_ (500 MHz, 293 K).

Figure S2. ^13^C{^1^H}-NMR spectrum of **PDI**–**NO_2_** in CDCl_3_ (125 MHz, 293 K).

Figure S3. ^1^H-NMR spectrum of **PDIN**–**H** in CDCl_3_ (500 MHz, 293 K).

Figure S4. ^13^C{^1^H}-NMR spectrum of **PDIN**–**H** in CDCl_3_ (125 MHz, 293 K).

Figure S5. ^1^H-NMR spectrum of **PDI**–**MeBZ** in DMF-d_7_ (500 MHz, 293 K).

Figure S6. ^13^C{^1^H}-NMR spectrum of **PDI**–**MeBZ** in CDCl_3_ (125 MHz, 293 K).

 Figure S7. ^1^H-NMR spectrum of [**PDI**–**MeBZ**]**^•−^** in DMF-d_7_ (500 MHz, 293 K).

Figure S8. ^1^H-NMR spectrum of **PDI**–**MeNP** in DMF-d_7_ (500 MHz, 293 K).

Figure S9. ^13^C{^1^H}-NMR spectrum of **PDI**–**MeNP** in CDCl_3_ (125 MHz, 293 K).

 Figure S10. ^1^H-NMR spectrum of [**PDI**–**MeNP**]**^•−^** in DMF-d_7_ (500 MHz, 293 K).

Figure S11. ^1^H-NMR spectrum of **PDI**–**MePY** in DMF-d_7_ (500 MHz, 293 K).

Figure S12. ^13^C{^1^H}-NMR spectrum of **PDI**–**MePY** in CDCl_3_ (125 MHz, 293 K).

 Figure S13. ^1^H-NMR spectrum of [**PDI**–**MePY**]**^•−^** in DMF-d_7_ (500 MHz, 293 K).

**(A)**

**(B)**

**(C)**

Figure S14. GC-MS data of (A) **PDI**–**MeBZ**, (B) **PDI**–**MeNP**, and (C) **PDI**–**MePY**.

**(B)**

**(A)**

**(C)**

Figure S15. ICP–OES spectra of (A) [**PDI**–**MeBZ**]**^•−^**, (B) [**PDI**–**MeNP**]**^•−^**, and (C) [**PDI**–**MePY**]**^•−^**, showing the characteristic emission line of Co^3+^ at 228.616 nm.

Figure S16. Cyclic voltammogram for the oxidation and reduction of **Cobaltocene** (1 mM) containing 0.1 M TBAP as a supporting electrolyte at room temperature under an argon atmosphere (scan rate= 50 mV s^−1^).

Table S1. Photophysical parameters of the synthesized **PDI**–**MeR** and their chemically generated radical anions [**PDI**–**MeR**]^•−^

| Compound | λ_abs_ /  nm ^a,b)^ | ε /  10^3^ M^–1^ cm^–1 a,b)^ | λ_em_ /  nm ^a,c)^ | Stokes shift /  nm ^a,b,c)^ | *Φ*_PL_ /  % ^a,d)^ | *τ*_PL_/  ns ^a,e)^ | k_r_ /  10^7^ s^–1 a,f)^ | k_nr_ /  10^7^ s^–1 a,g)^ |
| --- | --- | --- | --- | --- | --- | --- | --- | --- |
| **PDI**–**MeBZ** | 525 | 38.08 | 544 | 19 | 65.04 | 3.85 | 16.89 | 9.08 |
| **PDI**–**MeNP** | 526 | 37.46 | 544 | 20 | 61.00 | 3.64 | 16.76 | 10.71 |
| **PDI**–**MePY** | 526 | 34.19 | - | - | 0 | 3.34 | 0 | 29.94 |
| [**PDI**–**MeBZ**]^•−^ | 687,780,  839,928 | 37.71,10.62,  7.96,13.08 | 624 | - | 19.99 | 4.46 | 4.482 | 17.94 |
| [**PDI**–**MeNP**]^•−^ | 688,780,  839,928 | 42.78,12.21,  9.28,15.58 | 624 | - | 23.00 | 4.64 | 4.96 | 16.60 |
| [**PDI**–**MePY**]^•−^ | 688,780,  839,928 | 37.41,10.03,  7.65,13.50 | 625 | - | 29.33 | 6.33 | 4.63 | 11.16 |

^a)^Sample in 0.1mM *N,N*-dimethylformamide solution.

^b)^Peaks of UV-vis absorption.

^c)^Photoluminescence peak wavelength, λ_ex_ = 520 nm for PDI–MeR and 610 nm for [PDI–MeR]^•−^

^d)^Photoluminescence quantum efficiency, λ_ex_ = 520 nm for PDI–MeR and 609 nm for [PDI–MeR]^•−^

^e)^Photoluminescence lifetime, λ_ex_ = 525 nm for PDI–MeR and 610 nm for [PDI–MeR]^•−^

^f)^Radiative decay rate.

^g)^Nonradiative decay rate.

Table S2. Cyclic voltammetry data for the observed reduction waves of **PDI**–**MeR** measured in DCM with 0.1 M TBAP *vs. SCE*

|  | 1e^–^ waves (0/–1) | | | | 1e^–^ waves (–1/–2) | | | |
| --- | --- | --- | --- | --- | --- | --- | --- | --- |
| Compound | E_onset_ ^red1^ /  eV ^a)^ | E_pc_ ^red1^ /  eV ^b)^ | E_pa_ ^red1^ /  eV ^b)^ | E_1/2_ ^red1^ /  eV ^b)^ | E_onset_ ^red2^ /  eV ^a)^ | E_pc_ ^red2^ /  eV ^b)^ | E_pa_ ^red2^ /  eV ^b)^ | E_1/2_ ^red2^ /  eV ^b)^ |
| **PDI**–**MeBZ** | -0.6611 | -0.8317 | -0.6830 | -0.7574 | -0.9439 | -1.0788 | -0.9375 | -1.0082 |
| **PDI**–**MeNP** | -0.6365 | -0.8408 | -0.6502 | -0.7455 | -0.9530 | -1.0962 | -0.9129 | -1.0046 |
| **PDI**–**MePY** | -0.6247 | -0.8207 | -0.6420 | -0.7314 | -0.9366 | -1.0725 | -0.9028 | -0.9877 |

^a)^Onset potentials of the first and second reversible reduction waves.
^b)^E_1/2_ = (E_pa_ + E_pc_) / 2, in which E_pa_ (E_pc_) are anodic (cathodic) peak potentials in cyclic voltammetry.

Table S3. Cyclic voltammetry data for the observed reduction waves of **PDI**–**MeR** and [**PDI**–**MeR**]^•−^ measured in DMF with 0.1 M TBAP *vs. SCE*

|  | 1e^–^ waves (0/–1) | | | | 1e^–^ waves (–1/–2) | | | |
| --- | --- | --- | --- | --- | --- | --- | --- | --- |
| Compound | E_onset_ ^red1^ /  eV ^a)^ | E_pc_ ^red1^ /  eV ^b)^ | E_pa_ ^red1^ /  eV ^b)^ | E_1/2_ ^red1^ /  eV ^b)^ | E_onset_ ^red2^ /  eV ^a)^ | E_pc_ ^red2^ /  eV ^b)^ | E_pa_ ^red2^ /  eV ^b)^ | E_1/2_ ^red2^ /  eV ^b)^ |
| **PDI**–**MeBZ** | -0.8830 | -0.9569 | -0.8344 | -0.8956 | - | - | - | - |
| **PDI**–**MeNP** | -0.8755 | -0.9675 | -0.7946 | -0.8811 | - | - | - | - |
| **PDI**–**MePY** | -0.8777 | -0.9608 | -0.7985 | -0.8830 | - | - | - | - |
| [**PDI**–**MeBZ**]^•−^ | -0.6766 | -0.6942 | -0.6401 | -0.6672 | -0.9072 | -0.9723 | -0.8445 | -0.9084 |
| [**PDI**–**MeNP**]^•−^ | -0.6611 | -0.6858 | -0.6268 | -0.6563 | -0.8828 | -0.9361 | -0.8344 | -0.8853 |
| [**PDI**–**MePY**]^•−^ | -0.7979 | -0.6880 | -0.9176 | -0.8028 | -1.1604 | -0.9530 | -1.3564 | -1.1547 |

^a)^Onset potentials of the first and second reversible reduction waves.
^b)^E_1/2_ = (E_pa_ + E_pc_) / 2, in which E_pa_ (E_pc_) are anodic (cathodic) peak potentials in cyclic voltammetry.

**(A)**

**(B)**

**(C)**

Figure S17. UV-vis absorption and fluorescence spectra in DMF solution of (A) **PDI**–**MeBZ**, (B) **PDI**–**MeNP**, and (C) **PDI**–**MePY**.

**(B)**

**(A)**

**(C)**

Figure S18. UV–vis absorption and fluorescence spectra in different solvents of (A) **PDI**–**MeBZ**, (B) **PDI**–**MeNP**, and (C) **PDI**–**MePY**.

**(B)**

**(A)**

Figure S19. Cyclic voltammogram for the oxidation and reduction of **PDI**–**MeBZ**, **PDI**–**MeNP**, and **PDI**–**MePY** in (A) DCM and (B) DMF solution (1 mM) containing 0.1 M TBAP as a supporting electrolyte at room temperature under an argon atmosphere (scan rate= 50 mV s^−1^).

Figure S20. Cyclic voltammograms for the reduction of **PDI**–**MeBZ**, **PDI**–**MeNP**, and **PDI**–**MePY** in DMF solution (1 mM) containing 0.1 M TBAP as a supporting electrolyte at room temperature under an argon atmosphere (scan rate = 50 mV s⁻¹), recorded over 200 consecutive cycles.

Figure S21. Continuous-wave (CW) EPR spectra of [**PDI**–**MeBZ**]**^•−^**, [**PDI**–**MeNP**]**^•−^**, and
[**PDI**–**MePY**]**^•−^** (2 mM in DMF at room temperature) measured immediately after synthesis, confirming the formation and persistence of radical anions.

**(A)**

**(B)**

**(C)**

Figure S22. Comparison of cyclic voltammograms for the reduction of the neutral and chemically generated radical anion states of (A) **PDI**–**MeBZ**, [**PDI**–**MeBZ**]**^•−^**, (B) **PDI**–**MeNP**, [**PDI**–**MeNP**]**^•−^**, and (C) **PDI**–**MePY**, [**PDI**–**MePY**]**^•−^** in DMF solution (1 mM) containing 0.1 M TBAP as a supporting electrolyte at room temperature under an argon atmosphere (scan rate = 50 mV s^−1^).

**(A)**

**(B)**

**(C)**

Figure S23. UV-vis absorption spectral change during the electrochemical reduction from the neutral species to the corresponding radical anion species (1 mM, 0.1 M TBAP in THF): (A) **PDI**–**MeBZ** to [**PDI**–**MeBZ**]**^•−^**, (B) **PDI**–**MeNP** to [**PDI**–**MeNP**]**^•−^**, and (C) **PDI**–**MePY** to [**PDI**–**MePY**]**^•−^**.

**(C)**

**(B)**

**(A)**

Figure S24. Doublet emission stability of chemically generated [**PDI**–**MeR**]**^•−^** radical anions and corresponding photographs of emissive solution in a quartz cell under 610 nm laser excitation monitored from Day 1 to Day 60: (A) [**PDI**–**MeBZ**]**^•−^**, (B) [**PDI**–**MeNP**]**^•−^**, and (C) [**PDI**–**MePY**]**^•−^**.

**Calculation Details**

Quantum chemical calculation were carried out using Gaussian 16 software package.^[7]^ The ground-state geometry of **PDI**–**MeR** and [**PDI**–**MeR**]**^•−^** has been optimized at the density function theory (DFT) level. Full geometry optimizations in their ground singlet and doublet state were performed under the B3LYP function with the 6-31G(d,p) basis set for **PDI**–**MeR**, and under the B3LYP function with the 6-31+G(d,p) basis set for [**PDI**–**MeR**]**^•−^**. All of the Cartesian coordinates for optimized structure of **PDI**–**MeR** (Table S4, S6, and S8) and [**PDI**–**MeR**]**^•−^** (Table S5, S7, and S9) are also summarized. The frontier molecular orbitals (Figure S27-S32) were generated at an isovalue of 0.02 a.u.

On basis of the geometry optimization at ground state, time-dependent density functional theory (TDDFT) at B3LYP/6-31+G(d) was used to calculate 50 vertical excitation and oscillator strengths, and the simulated absorption spectra and transition assignments were obtained by the GaussSum program. Afterwards, analyzing wavefunction by Multiwfn, electron-hole distribution^[8]^ could be obtained. The spin density maps obtained from ca. 1770000 grid points for [**PDI**–**MeR**]**^•−^** used Multiwfn (version 3.8)^[9]^ and VMD (version 1.9.3)^[10]^ for analysis and visualization.

To conduct an in‐depth analysis of the aromaticity characteristics, anisotropy of the induced current density (AICD)^[11]^ and gauge-including magnetically induced current (GIMIC)^[12]^ calculations were performed using the CSGT method (CSGT/B3LYP/6-31G(d)), based on the optimized ground-state geometries. In both calculations, the external magnetic field was applied along the z-axis, perpendicular to the molecular plane. AICDplots, rendered by POV-Ray (version 3.7) software^[13]^, represent the density of delocalized electrons. The current density vectors (arrows on the AICDisosurface) indicate a diatropic or paratropic ring current depending on the clockwise or counterclockwise direction, respectively. The magnetically induced current pathways were visualized using Paraview^[14]^.

**(A)**

Figure S25. Spin density maps of radical anion states calculated at B3LYP/6-31+G(d,p) level (isovalue = 0.001 a.u.). (A) [**PDI**–**MeBZ**]**^•−^**, (B) [**PDI**–**MeNP**]**^•−^**, and (C) [**PDI**–**MePY**]**^•−^**.

**(B)**

**(C)**

**(C)**

**(D)**

**(B)**

**(A)**

Figure S26. AICDplots of neutral and radical anion states calculated at B3LYP/6-31G(d,p) and B3LYP/6-31G(d) level (isovalue = 0.05). (A) **PDI**–**MeBZ**, (B) [**PDI**–**MeBZ**]**^•−^**, (C) **PDI**–**MeNP**, and (D) [**PDI**–**MeNP**]**^•−^**.

Figure S27. (A) Energy levels and isodensity plots (isovalue = 0.02 a.u.) of **PDI**–**MeBZ**,
(B) Electronic transition of simulated (lines)/experimental (sticks) absorption spectra of **PDI**–**MeBZ**.

**(A)**

**(B)**

Figure S28. (A) Energy levels and isodensity plots (isovalue = 0.02 a.u.) of **PDI**–**MeNP**,
(B) Electronic transition of simulated (lines)/experimental (sticks) absorption spectra of **PDI**–**MeNP**.

**(B)**

**(A)**

Figure S29. (A) Energy levels and isodensity plots (isovalue = 0.02 a.u.) of **PDI**–**MePY**,
(B) Electronic transition of simulated (lines)/experimental (sticks) absorption spectra of **PDI**–**MePY**.

**(A)**

**(B)**

Figure S30. (A) Energy levels and isodensity plots (isovalue = 0.02 a.u.) of [**PDI**–**MeBZ**]**^•−^**, (B) Electronic transition of simulated (lines)/experimental (sticks) absorption spectra of [**PDI**–**MeBZ**]**^•−^**.

Figure S31. (A) Energy levels and isodensity plots (isovalue = 0.02 a.u.) of [**PDI**–**MeNP**]**^•−^**, (B) Electronic transition of simulated (lines)/experimental (sticks) absorption spectra of [**PDI**–**MeNP**]**^•−^**.

**(B)**

**(A)**

**(B)**

**(A)**

Figure S32. (A) Energy levels and isodensity plots (isovalue = 0.02 a.u.) of [**PDI**–**MePY**]**^•−^**, (B) Electronic transition of simulated (lines)/experimental (sticks) absorption spectra of [**PDI**–**MePY**]**^•−^**.

**(A)**

**(B)**

Table S4. Cartesian coordinates for optimized structure for **PDI**–**MeBZ**

Symbolic Z-matrix:

Charge = 0 Multiplicity = 1

| Atom | X | Y | Z | Atom | X | Y | Z |
| --- | --- | --- | --- | --- | --- | --- | --- |
| C | 2.47098 | 1.48606 | 0.686398 | C | -0.88079 | 4.188508 | 0.235968 |
| C | 1.085231 | 1.202371 | 0.626335 | C | -0.33644 | 4.273067 | -1.05225 |
| C | 0.739413 | -0.09226 | 0.244828 | C | -0.89838 | 5.125205 | -2.00044 |
| C | 1.569977 | -1.14879 | -0.08417 | C | -2.00979 | 5.906134 | -1.67198 |
| C | 2.947306 | -0.86289 | -0.02301 | C | -2.5563 | 5.828586 | -0.39194 |
| C | 3.36482 | 0.453822 | 0.36265 | C | -1.99388 | 4.971319 | 0.556437 |
| C | 1.004731 | -2.41331 | -0.44988 | H | 2.890357 | 2.446815 | 0.965351 |
| C | 1.945285 | -3.40604 | -0.76405 | H | 1.619907 | -4.40038 | -1.05409 |
| C | 3.327619 | -3.14379 | -0.71244 | H | 4.035531 | -3.92759 | -0.96034 |
| C | 3.841521 | -1.89478 | -0.34771 | H | -3.19462 | 1.924637 | 0.902386 |
| C | -0.63032 | -0.21185 | 0.236728 | H | -3.19773 | -4.55859 | -1.00116 |
| C | -1.26059 | -1.39681 | -0.09923 | H | -0.7365 | -4.60608 | -1.06852 |
| C | -0.48094 | -2.54324 | -0.45785 | H | 7.197621 | 0.959604 | 0.575048 |
| C | -1.20201 | 1.002126 | 0.609518 | H | -7.15246 | -2.46032 | 0.19379 |
| C | -2.61697 | 1.043389 | 0.648542 | H | 7.166673 | -1.49568 | 1.776278 |
| C | -3.31299 | -0.13065 | 0.323592 | H | 8.399917 | -1.79505 | 0.579236 |
| C | -2.66702 | -1.35416 | -0.05169 | H | 7.67413 | -0.98581 | -1.72019 |
| C | -3.36533 | -2.52384 | -0.38511 | H | 7.10512 | 0.676973 | -1.85766 |
| C | -2.63914 | -3.66453 | -0.74551 | H | 9.408372 | -0.97047 | 2.712606 |
| C | -1.23199 | -3.68353 | -0.78204 | H | 9.683713 | 0.24747 | 1.464686 |
| C | 4.822467 | 0.727269 | 0.415502 | H | 8.406493 | 0.486283 | 2.660108 |
| C | 5.297533 | -1.6342 | -0.29722 | H | 9.510864 | 0.599318 | -2.37554 |
| C | -4.79949 | -0.11231 | 0.351466 | H | 9.295673 | 1.495383 | -0.86725 |
| C | -4.84344 | -2.52206 | -0.34448 | H | 9.881958 | -0.17547 | -0.83663 |
| N | -5.47375 | -1.319 | 0.066728 | H | -7.49569 | 0.307012 | 1.419318 |
| N | 5.696291 | -0.34273 | 0.102749 | H | -8.64633 | -1.02489 | 1.379502 |
| O | 5.259064 | 1.831545 | 0.719622 | H | -7.20965 | -1.51608 | -1.98583 |
| O | 6.124034 | -2.49227 | -0.58153 | H | -8.69384 | -1.24798 | -1.10046 |
| O | -5.41582 | 0.912768 | 0.618038 | H | -7.52673 | -0.96241 | 3.58541 |
| O | -5.49856 | -3.51193 | -0.64528 | H | -7.04628 | -2.44034 | 2.73995 |
| N | -0.12887 | 1.886912 | 0.856643 | H | -5.93488 | -1.06644 | 2.834803 |
| C | 7.163677 | -0.04919 | 0.161348 | H | -8.16901 | 0.733117 | -2.45349 |
| C | -6.97306 | -1.3853 | 0.141213 | H | -8.04889 | 1.183645 | -0.7476 |
| C | 7.903941 | -0.99699 | 1.139139 | H | -6.58551 | 0.919812 | -1.68727 |
| C | 7.742259 | 0.006973 | -1.26705 | H | -0.85241 | 3.313339 | 2.20355 |
| C | 8.907862 | -0.26558 | 2.040496 | H | 0.746208 | 3.62278 | 1.542008 |
| C | 9.188055 | 0.509469 | -1.33344 | H | 0.526106 | 3.666121 | -1.31433 |
| C | -7.57669 | -0.77958 | 1.420036 | H | -0.46875 | 5.181907 | -2.99615 |
| C | -7.6458 | -0.92692 | -1.17039 | H | -2.44718 | 6.569061 | -2.41237 |
| C | -6.98598 | -1.34623 | 2.714595 | H | -3.42298 | 6.428012 | -0.1303 |
| C | -7.60518 | 0.561945 | -1.52996 | H | -2.42514 | 4.91168 | 1.552826 |
| C | -0.26008 | 3.279833 | 1.282478 |  |  |  |  |

Table S5. Cartesian coordinates for optimized structure for [**PDI**–**MeBZ**]**^•−^**

Symbolic Z-matrix:

Charge = -1 Multiplicity = 2

| Atom | X | Y | Z | Atom | X | Y | Z |
| --- | --- | --- | --- | --- | --- | --- | --- |
| C | 2.407078 | 1.402334 | 0.696294 | C | -0.43389 | 4.303112 | 0.228122 |
| C | 1.032765 | 1.158717 | 0.627592 | C | -0.24438 | 4.011526 | -1.12639 |
| C | 0.635938 | -0.12882 | 0.235099 | C | -0.39061 | 5.010616 | -2.09396 |
| C | 1.43555 | -1.21203 | -0.09203 | C | -0.72773 | 6.312865 | -1.71928 |
| C | 2.830421 | -0.97304 | -0.02074 | C | -0.92148 | 6.611437 | -0.36668 |
| C | 3.288614 | 0.324734 | 0.371311 | C | -0.77666 | 5.611934 | 0.596867 |
| C | 0.821029 | -2.45406 | -0.46088 | H | 2.851257 | 2.351819 | 0.974742 |
| C | 1.738711 | -3.49362 | -0.77807 | H | 1.37339 | -4.47366 | -1.07073 |
| C | 3.11626 | -3.28238 | -0.7204 | H | 3.796287 | -4.09183 | -0.96768 |
| C | 3.689857 | -2.04404 | -0.34651 | H | -3.22749 | 2.010431 | 0.917535 |
| C | -0.73485 | -0.20604 | 0.22343 | H | -3.41135 | -4.49637 | -1.02592 |
| C | -1.4019 | -1.37234 | -0.11416 | H | -0.96058 | -4.60465 | -1.0906 |
| C | -0.64644 | -2.53673 | -0.47251 | H | 7.106752 | 0.693833 | 0.588571 |
| C | -1.28148 | 1.028049 | 0.606484 | H | -7.30403 | -2.27857 | 0.140678 |
| C | -2.67552 | 1.115377 | 0.65256 | H | 6.997639 | -1.78557 | 1.773179 |
| C | -3.42381 | -0.05592 | 0.319282 | H | 8.263782 | -2.07274 | 0.609605 |
| C | -2.81552 | -1.2919 | -0.06279 | H | 7.553907 | -1.27411 | -1.69165 |
| C | -3.54524 | -2.45062 | -0.40124 | H | 7.019774 | 0.40092 | -1.84199 |
| C | -2.83059 | -3.61621 | -0.76786 | H | 9.217606 | -1.27652 | 2.783481 |
| C | -1.43739 | -3.67194 | -0.80375 | H | 9.529425 | -0.04372 | 1.554737 |
| C | 4.732822 | 0.544249 | 0.435167 | H | 8.218925 | 0.185331 | 2.717429 |
| C | 5.132471 | -1.84225 | -0.29115 | H | 9.434558 | 0.271757 | -2.33587 |
| C | -4.88713 | 0.002163 | 0.353543 | H | 9.213428 | 1.185104 | -0.83563 |
| C | -5.00049 | -2.41499 | -0.36244 | H | 9.770456 | -0.49641 | -0.7829 |
| N | -5.59724 | -1.18607 | 0.047753 | H | -7.57305 | 0.462046 | 1.444044 |
| N | 5.572686 | -0.55989 | 0.114283 | H | -8.76474 | -0.83997 | 1.36387 |
| O | 5.232788 | 1.632906 | 0.755012 | H | -7.32974 | -1.27638 | -2.00917 |
| O | 5.959458 | -2.72192 | -0.57029 | H | -8.8137 | -1.00435 | -1.11773 |
| O | -5.50288 | 1.038009 | 0.640222 | H | -7.64123 | -0.86304 | 3.575785 |
| O | -5.70944 | -3.38652 | -0.66118 | H | -7.20356 | -2.33336 | 2.686385 |
| N | -0.17118 | 1.880575 | 0.860337 | H | -6.05362 | -0.99347 | 2.81193 |
| C | 7.041245 | -0.31619 | 0.179991 | H | -8.24931 | 0.999592 | -2.42683 |
| C | -7.09062 | -1.20815 | 0.119578 | H | -8.12164 | 1.408153 | -0.70654 |
| C | 7.753707 | -1.27927 | 1.165221 | H | -6.662 | 1.136445 | -1.64597 |
| C | 7.637694 | -0.27999 | -1.24303 | H | -1.08659 | 3.324841 | 2.030973 |
| C | 8.736278 | -0.56215 | 2.104385 | H | 0.658159 | 3.466735 | 1.884615 |
| C | 9.094788 | 0.196567 | -1.29636 | H | 0.01362 | 3.000415 | -1.42419 |
| C | -7.68781 | -0.62171 | 1.412293 | H | -0.2421 | 4.766141 | -3.14208 |
| C | -7.758 | -0.70115 | -1.17917 | H | -0.84354 | 7.087257 | -2.47248 |
| C | -7.11313 | -1.24008 | 2.692131 | H | -1.19099 | 7.619556 | -0.06314 |
| C | -7.69015 | 0.796256 | -1.50466 | H | -0.93639 | 5.849665 | 1.646904 |
| C | -0.25582 | 3.249425 | 1.318011 |  |  |  |  |

Table S6. Cartesian coordinates for optimized structure for **PDI**–**MeNP**

Symbolic Z-matrix:

Charge = 0 Multiplicity = 1

| Atom | X | Y | Z | Atom | X | Y | Z |
| --- | --- | --- | --- | --- | --- | --- | --- |
| C | 2.557482 | 0.64826 | 1.185767 | C | -0.08132 | 6.093037 | -0.10658 |
| C | 1.158247 | 0.473013 | 1.06477 | C | -0.04901 | 4.834553 | -0.78801 |
| C | 0.735408 | -0.66292 | 0.379042 | C | -0.04043 | 3.634433 | -0.025 |
| C | 1.502111 | -1.66027 | -0.19693 | C | -0.08984 | 7.290042 | -0.86973 |
| C | 2.893599 | -1.48442 | -0.0734 | C | -0.06747 | 7.246963 | -2.24579 |
| C | 3.38855 | -0.32949 | 0.617571 | C | -0.0355 | 6.002623 | -2.92048 |
| C | 0.863464 | -2.76441 | -0.84847 | C | -0.02648 | 4.824444 | -2.20785 |
| C | 1.744224 | -3.71095 | -1.39336 | H | 3.028704 | 1.488488 | 1.683771 |
| C | 3.139572 | -3.55501 | -1.28677 | H | 1.361526 | -4.58653 | -1.90872 |
| C | 3.725833 | -2.46311 | -0.63783 | H | 3.800382 | -4.30059 | -1.71608 |
| C | -0.6394 | -0.69465 | 0.355166 | H | -3.07422 | 1.347744 | 1.576852 |
| C | -1.33879 | -1.72597 | -0.2463 | H | -3.45816 | -4.46812 | -1.84295 |
| C | -0.62758 | -2.79877 | -0.87445 | H | -1.00396 | -4.6408 | -1.95049 |
| C | -1.13766 | 0.419976 | 1.024858 | H | 7.240334 | -0.06076 | 0.834147 |
| C | -2.54711 | 0.530453 | 1.09686 | H | -7.33898 | -2.26515 | -0.51726 |
| C | -3.31149 | -0.48419 | 0.501434 | H | 7.776409 | -3.05251 | 0.578898 |
| C | -2.74038 | -1.6146 | -0.17127 | H | 8.868188 | -1.83214 | 1.219408 |
| C | -3.50656 | -2.63013 | -0.76348 | H | 8.898961 | -0.59448 | -0.86874 |
| C | -2.8481 | -3.69317 | -1.39129 | H | 7.809354 | -1.72799 | -1.65577 |
| C | -1.44428 | -3.78437 | -1.44919 | H | 7.758582 | -3.01651 | 3.096083 |
| C | 4.859944 | -0.17272 | 0.732859 | H | 7.22138 | -1.32942 | 3.079042 |
| C | 5.195637 | -2.31675 | -0.53169 | H | 6.162006 | -2.60614 | 2.469056 |
| C | -4.79319 | -0.3952 | 0.563045 | H | 7.285197 | 1.299133 | -1.3552 |
| C | -4.98307 | -2.55307 | -0.70823 | H | 7.847498 | 0.498877 | -2.83117 |
| N | -5.53597 | -1.43209 | -0.03952 | H | 6.231748 | 0.164782 | -2.20812 |
| N | 5.673331 | -1.17836 | 0.14859 | H | -7.31435 | -0.54325 | 1.999665 |
| O | 5.357828 | 0.78596 | 1.310805 | H | -8.66258 | -1.48564 | 1.377997 |
| O | 5.962687 | -3.14342 | -1.0105 | H | -7.34695 | 0.772235 | -0.24555 |
| O | -5.35543 | 0.542178 | 1.118315 | H | -6.99699 | -0.10355 | -1.73007 |
| O | -5.69411 | -3.41268 | -1.2124 | H | -7.56543 | -2.74501 | 3.200881 |
| N | -0.01344 | 1.148417 | 1.475091 | H | -7.32551 | -3.61359 | 1.677084 |
| C | 7.154834 | -0.99566 | 0.278739 | H | -6.00641 | -2.6699 | 2.379206 |
| C | -7.03197 | -1.36096 | 0.010972 | H | -9.3622 | 0.583926 | -1.74941 |
| C | 7.809568 | -2.1051 | 1.12235 | H | -9.29133 | -1.17918 | -1.63977 |
| C | 7.842098 | -0.79687 | -1.08482 | H | -9.67333 | -0.2118 | -0.20829 |
| C | 7.203057 | -2.27065 | 2.518943 | H | -0.94449 | 2.386096 | 2.846762 |
| C | 7.268584 | 0.356653 | -1.91321 | H | 0.805334 | 2.422564 | 2.885099 |
| C | -7.56933 | -1.45332 | 1.451114 | H | -0.11151 | 4.939119 | 3.10932 |
| C | -7.55818 | -0.15207 | -0.78872 | H | -0.12795 | 7.046103 | 1.838476 |
| C | -7.08897 | -2.69027 | 2.216988 | H | -0.0159 | 2.685816 | -0.5528 |
| C | -9.0543 | -0.24938 | -1.11003 | H | -0.11435 | 8.243373 | -0.34844 |
| C | -0.05514 | 2.405058 | 2.206169 | H | -0.07423 | 8.168768 | -2.81969 |
| C | -0.06243 | 3.663482 | 1.350648 | H | -0.018 | 5.981326 | -4.00603 |
| C | -0.09436 | 4.91697 | 2.022119 | H | -0.00187 | 3.868732 | -2.72465 |
| C | -0.10358 | 6.094129 | 1.314862 |  |  |  |  |

Table S7. Cartesian coordinates for optimized structure for [**PDI**–**MeNP**]**^•−^**

Symbolic Z-matrix:

Charge = -1 Multiplicity = 2

| Atom | X | Y | Z | Atom | X | Y | Z |
| --- | --- | --- | --- | --- | --- | --- | --- |
| C | 2.551729 | 0.69099 | 1.136785 | C | -0.13947 | 6.171369 | -0.0775 |
| C | 1.171987 | 0.507946 | 1.014131 | C | -0.08316 | 4.925085 | -0.78192 |
| C | 0.741628 | -0.65126 | 0.350819 | C | -0.0578 | 3.708953 | -0.04304 |
| C | 1.51326 | -1.66173 | -0.19975 | C | -0.16487 | 7.383053 | -0.81996 |
| C | 2.913563 | -1.48365 | -0.07645 | C | -0.13581 | 7.367511 | -2.19877 |
| C | 3.405271 | -0.3157 | 0.5871 | C | -0.0799 | 6.134776 | -2.89667 |
| C | 0.867753 | -2.78059 | -0.82187 | C | -0.05427 | 4.942863 | -2.20349 |
| C | 1.759032 | -3.75507 | -1.35038 | H | 3.019314 | 1.543831 | 1.616512 |
| C | 3.141424 | -3.59995 | -1.24702 | H | 1.369338 | -4.64097 | -1.84322 |
| C | 3.745856 | -2.4852 | -0.61907 | H | 3.800865 | -4.35839 | -1.65764 |
| C | -0.63081 | -0.6889 | 0.328292 | H | -3.06642 | 1.379195 | 1.511768 |
| C | -1.32743 | -1.73965 | -0.24668 | H | -3.41411 | -4.55229 | -1.78435 |
| C | -0.60141 | -2.82044 | -0.84674 | H | -0.96666 | -4.70261 | -1.88612 |
| C | -1.14546 | 0.444652 | 0.975473 | H | 7.228308 | -0.05263 | 0.80856 |
| C | -2.53669 | 0.552554 | 1.050987 | H | -7.30055 | -2.37032 | -0.4497 |
| C | -3.31436 | -0.49919 | 0.475256 | H | 7.78149 | -3.04569 | 0.620934 |
| C | -2.7387 | -1.63839 | -0.16995 | H | 8.863097 | -1.80115 | 1.248153 |
| C | -3.49716 | -2.68233 | -0.74123 | H | 8.90301 | -0.61459 | -0.86546 |
| C | -2.81152 | -3.76051 | -1.35013 | H | 7.82403 | -1.78033 | -1.63278 |
| C | -1.42019 | -3.84042 | -1.40601 | H | 7.738892 | -2.97275 | 3.133714 |
| C | 4.854501 | -0.15982 | 0.704279 | H | 7.201176 | -1.282 | 3.087518 |
| C | 5.193817 | -2.34504 | -0.51541 | H | 6.147234 | -2.56535 | 2.48275 |
| C | -4.77457 | -0.42132 | 0.538016 | H | 7.285379 | 1.25276 | -1.426 |
| C | -4.952 | -2.62226 | -0.68771 | H | 7.854237 | 0.404839 | -2.87754 |
| N | -5.51173 | -1.48968 | -0.03043 | H | 6.237405 | 0.085492 | -2.23985 |
| N | 5.670693 | -1.18607 | 0.1427 | H | -7.32199 | -0.49364 | 1.954038 |
| O | 5.377359 | 0.813803 | 1.265086 | H | -8.63915 | -1.52434 | 1.391228 |
| O | 5.990706 | -3.17933 | -0.96839 | H | -7.33914 | 0.676846 | -0.36102 |
| O | -5.37261 | 0.528308 | 1.064688 | H | -6.98906 | -0.2883 | -1.79086 |
| O | -5.685 | -3.49369 | -1.17632 | H | -7.50252 | -2.61272 | 3.303473 |
| N | -0.01314 | 1.19325 | 1.403004 | H | -7.22948 | -3.57735 | 1.839667 |
| C | 7.144494 | -1.00089 | 0.274571 | H | -5.94496 | -2.54012 | 2.471107 |
| C | -7.00075 | -1.43316 | 0.023907 | H | -9.36147 | 0.376593 | -1.85176 |
| C | 7.806652 | -2.08689 | 1.145829 | H | -9.27472 | -1.37661 | -1.62133 |
| C | 7.848781 | -0.83346 | -1.08662 | H | -9.66232 | -0.31593 | -0.25691 |
| C | 7.188629 | -2.23234 | 2.541356 | H | -0.94629 | 2.394913 | 2.803793 |
| C | 7.273959 | 0.292352 | -1.95423 | H | 0.808786 | 2.446358 | 2.827953 |
| C | -7.54687 | -1.44549 | 1.465717 | H | -0.16475 | 4.949194 | 3.115196 |
| C | -7.54657 | -0.28113 | -0.84586 | H | -0.21084 | 7.084661 | 1.888394 |
| C | -7.02637 | -2.61097 | 2.315977 | H | -0.01549 | 2.767533 | -0.58248 |
| C | -9.04438 | -0.4096 | -1.15676 | H | -0.20774 | 8.326796 | -0.28122 |
| C | -0.05979 | 2.424719 | 2.156964 | H | -0.15564 | 8.300695 | -2.75491 |
| C | -0.08624 | 3.707555 | 1.334017 | H | -0.05722 | 6.132079 | -3.98279 |
| C | -0.14204 | 4.949563 | 2.02728 | H | -0.01142 | 3.996743 | -2.73652 |
| C | -0.16793 | 6.143803 | 1.344805 |  |  |  |  |

Table S8. Cartesian coordinates for optimized structure for **PDI**–**MePY**

Symbolic Z-matrix:

Charge = 0 Multiplicity = 1

| Atom | X | Y | Z | Atom | X | Y | Z |
| --- | --- | --- | --- | --- | --- | --- | --- |
| C | -2.17761 | -0.38214 | -1.1783 | C | 0.520945 | 4.160841 | 1.205548 |
| C | -0.76563 | -0.30908 | -1.07659 | C | 0.394333 | 3.393482 | 0.087503 |
| C | -0.15123 | -1.33165 | -0.35533 | C | -3.81223 | 6.295946 | 0.257436 |
| C | -0.73332 | -2.41354 | 0.282898 | C | -2.65968 | 6.175951 | 1.107191 |
| C | -2.13566 | -2.48046 | 0.1793 | C | -2.50459 | 6.949199 | 2.268177 |
| C | -2.82444 | -1.45911 | -0.55419 | C | -1.37441 | 6.812682 | 3.072745 |
| C | 0.087656 | -3.36257 | 0.973649 | C | -0.37507 | 5.902763 | 2.737249 |
| C | -0.61521 | -4.41516 | 1.579654 | C | -8.57888 | -2.21305 | 1.176333 |
| C | -2.01744 | -4.5004 | 1.493628 | C | 9.615382 | -0.69228 | -1.75549 |
| C | -2.78534 | -3.55515 | 0.804978 | H | -2.78747 | 0.344199 | -1.70092 |
| C | 1.209759 | -1.13611 | -0.35451 | H | -0.08448 | -5.18716 | 2.128337 |
| C | 2.07751 | -2.00323 | 0.285142 | H | -2.5392 | -5.32276 | 1.971447 |
| C | 1.562679 | -3.14673 | 0.9765 | H | 3.263042 | 1.220357 | -1.70583 |
| C | 1.507635 | 0.017075 | -1.07651 | H | 4.640531 | -4.26368 | 1.985703 |
| C | 2.879161 | 0.355678 | -1.17499 | H | 2.251399 | -4.84057 | 2.138033 |
| C | 3.808931 | -0.48641 | -0.54576 | H | -6.6677 | -1.85474 | -0.71912 |
| C | 3.440011 | -1.66266 | 0.184359 | H | 8.064075 | -1.76397 | 0.10974 |
| C | 4.370608 | -2.50246 | 0.813692 | H | -6.69188 | -4.87303 | -0.28209 |
| C | 3.905706 | -3.62697 | 1.504705 | H | -7.97707 | -3.89761 | -0.98145 |
| C | 2.537914 | -3.94975 | 1.587574 | H | -6.92935 | -3.43592 | 1.875641 |
| C | -4.30177 | -1.56012 | -0.65431 | H | -6.5707 | -1.71686 | 1.775818 |
| C | -4.26011 | -3.6597 | 0.724373 | H | -6.69976 | -4.98163 | -2.80002 |
| C | 5.252903 | -0.14371 | -0.62628 | H | -6.44781 | -3.23117 | -2.88486 |
| C | 5.811179 | -2.18182 | 0.725845 | H | -5.18859 | -4.27926 | -2.22179 |
| N | 6.169626 | -1.03743 | -0.03322 | H | 7.564023 | -1.12252 | -2.2603 |
| N | -4.92821 | -2.6567 | -0.00607 | H | 7.845873 | 0.555889 | -1.85135 |
| O | -4.96141 | -0.73352 | -1.27295 | H | 7.807095 | -0.23994 | 1.925738 |
| O | -4.87318 | -4.57321 | 1.263871 | H | 9.24852 | 0.057887 | 0.989435 |
| O | 5.631503 | 0.877317 | -1.18864 | H | 8.273974 | 2.196281 | 1.694393 |
| O | 6.661744 | -2.87135 | 1.274328 | H | 8.154736 | 2.093036 | -0.06624 |
| N | 0.273381 | 0.542725 | -1.51926 | H | 6.725774 | 1.822396 | 0.921845 |
| C | -6.42089 | -2.72786 | -0.11323 | H | 1.118712 | 2.179617 | -2.44521 |
| C | 7.647481 | -0.78652 | -0.13893 | H | -0.11384 | 1.375144 | -3.40641 |
| C | -6.88874 | -3.97918 | -0.8791 | H | -2.15931 | 2.302564 | -3.64384 |
| C | -7.08397 | -2.54186 | 1.266619 | H | -3.91985 | 3.917094 | -3.03781 |
| C | -6.27093 | -4.123 | -2.27375 | H | -4.81733 | 5.639636 | -1.50041 |
| C | 8.109312 | -0.46435 | -1.57281 | H | 1.401449 | 4.063342 | 1.834859 |
| C | 8.156558 | 0.162852 | 0.967803 | H | 1.177831 | 2.685555 | -0.15529 |
| C | 7.802674 | 1.650263 | 0.869848 | H | -4.58418 | 7.009608 | 0.532227 |
| C | 0.129923 | 1.714477 | -2.39307 | H | -3.28054 | 7.66129 | 2.535523 |
| C | -0.9142 | 2.718466 | -1.94437 | H | -1.27287 | 7.420305 | 3.966909 |
| C | -2.05065 | 2.895883 | -2.73936 | H | 0.504395 | 5.80016 | 3.36698 |
| C | -3.04537 | 3.808109 | -2.40306 | H | -8.98446 | -2.0042 | 2.171294 |
| C | -2.93199 | 4.589583 | -1.24554 | H | -8.75838 | -1.32961 | 0.552808 |
| C | -1.77425 | 4.438508 | -0.42071 | H | -9.15909 | -3.04095 | 0.757161 |
| C | -0.75517 | 3.496639 | -0.77196 | H | 9.902314 | -0.52694 | -2.79871 |
| C | -3.94138 | 5.539463 | -0.86556 | H | 10.21101 | -0.00768 | -1.14296 |
| C | -1.64055 | 5.238299 | 0.755068 | H | 9.905545 | -1.71504 | -1.48872 |
| C | -0.48743 | 5.106982 | 1.585311 |  |  |  |  |

Table S9. Cartesian coordinates for optimized structure for [**PDI**–**MePY**]**^•−^**

Symbolic Z-matrix:

Charge = -1 Multiplicity = 2

| Atom | X | Y | Z | Atom | X | Y | Z |
| --- | --- | --- | --- | --- | --- | --- | --- |
| C | 2.375171 | -0.20614 | 1.119653 | C | -1.38959 | 4.139639 | -0.97145 |
| C | 0.982234 | -0.25923 | 1.010753 | C | -0.96919 | 3.351539 | 0.058717 |
| C | 0.446965 | -1.34108 | 0.293664 | C | 2.557287 | 7.033584 | -0.39733 |
| C | 1.122426 | -2.37921 | -0.3283 | C | 1.338964 | 6.739029 | -1.10303 |
| C | 2.534177 | -2.32845 | -0.22132 | C | 0.883233 | 7.531718 | -2.17022 |
| C | 3.13168 | -1.24758 | 0.49948 | C | -0.30258 | 7.21957 | -2.83719 |
| C | 0.376294 | -3.40636 | -0.99499 | C | -1.05773 | 6.111546 | -2.45454 |
| C | 1.17399 | -4.41905 | -1.59558 | C | 8.91362 | -1.50897 | -1.16494 |
| C | 2.5661 | -4.38478 | -1.51348 | C | -9.30202 | -1.72006 | 1.833212 |
| C | 3.271051 | -3.36312 | -0.83497 | H | 2.921368 | 0.573122 | 1.636651 |
| C | -0.92416 | -1.27189 | 0.30548 | H | 0.703347 | -5.24059 | -2.12765 |
| C | -1.71458 | -2.23065 | -0.30752 | H | 3.152996 | -5.16978 | -1.98043 |
| C | -1.09106 | -3.3282 | -0.98635 | H | -3.16879 | 0.88048 | 1.699989 |
| C | -1.33246 | -0.14442 | 1.034141 | H | -4.05226 | -4.7789 | -1.94499 |
| C | -2.71014 | 0.05849 | 1.160422 | H | -1.6273 | -5.11178 | -2.12112 |
| C | -3.58212 | -0.89202 | 0.54345 | H | 6.961537 | -1.35278 | 0.721233 |
| C | -3.11161 | -2.02853 | -0.18349 | H | -7.67463 | -2.58401 | -0.08314 |
| C | -3.96324 | -2.97397 | -0.79218 | H | 7.276453 | -4.34577 | 0.226674 |
| C | -3.37945 | -4.06324 | -1.48273 | H | 8.44586 | -3.2603 | 0.980191 |
| C | -2.00053 | -4.24685 | -1.58062 | H | 7.375376 | -2.8569 | -1.89844 |
| C | 4.590104 | -1.22431 | 0.604587 | H | 6.871968 | -1.17522 | -1.76627 |
| C | 4.72725 | -3.34946 | -0.75278 | H | 7.238242 | -4.52119 | 2.738138 |
| C | -5.0296 | -0.69939 | 0.648447 | H | 6.81679 | -2.80201 | 2.860365 |
| C | -5.40587 | -2.80745 | -0.68701 | H | 5.679586 | -3.94511 | 2.135935 |
| N | -5.86436 | -1.68252 | 0.060971 | H | -7.21009 | -1.97146 | 2.298164 |
| N | 5.307637 | -2.28581 | -0.021 | H | -7.65162 | -0.31449 | 1.949 |
| O | 5.202502 | -0.33232 | 1.2094 | H | -7.56765 | -0.96707 | -1.84901 |
| O | 5.445384 | -4.21021 | -1.28105 | H | -9.04793 | -0.90849 | -0.92302 |
| O | -5.52854 | 0.278622 | 1.22301 | H | -8.37162 | 1.370805 | -1.53185 |
| O | -6.21746 | -3.58771 | -1.20529 | H | -8.25315 | 1.212528 | 0.228375 |
| N | -0.13642 | 0.503324 | 1.453259 | H | -6.79082 | 1.178044 | -0.74372 |
| C | 6.793083 | -2.2336 | 0.098531 | H | -1.11522 | 1.913738 | 2.589619 |
| C | -7.35029 | -1.57851 | 0.191466 | H | 0.366051 | 1.309898 | 3.319257 |
| C | 7.373137 | -3.45059 | 0.846699 | H | 2.22725 | 2.578533 | 3.345539 |
| C | 7.451437 | -1.9644 | -1.27105 | H | 3.563461 | 4.529843 | 2.651876 |
| C | 6.740863 | -3.69097 | 2.223156 | H | 3.902736 | 6.484394 | 1.160187 |
| C | -7.82506 | -1.34563 | 1.639333 | H | -2.30907 | 3.897053 | -1.49758 |
| C | -7.97904 | -0.65729 | -0.88071 | H | -1.55837 | 2.483515 | 0.327495 |
| C | -7.83539 | 0.861504 | -0.72103 | H | 3.140037 | 7.896902 | -0.70891 |
| C | -0.08054 | 1.633855 | 2.369262 | H | 1.468872 | 8.39569 | -2.47439 |
| C | 0.696195 | 2.842391 | 1.86445 | H | -0.63837 | 7.843157 | -3.6611 |
| C | 1.88193 | 3.193035 | 2.51798 | H | -1.97924 | 5.870536 | -2.97799 |
| C | 2.638877 | 4.296132 | 2.130815 | H | 9.305054 | -1.23992 | -2.1529 |
| C | 2.224494 | 5.1051 | 1.063592 | H | 9.011996 | -0.62847 | -0.51758 |
| C | 1.012093 | 4.777091 | 0.380392 | H | 9.562465 | -2.29419 | -0.76146 |
| C | 0.244047 | 3.636214 | 0.78075 | H | -9.58895 | -1.61759 | 2.886214 |
| C | 2.978731 | 6.252823 | 0.6364 | H | -9.96826 | -1.07352 | 1.250567 |
| C | 0.571091 | 5.60036 | -0.70267 | H | -9.49974 | -2.7568 | 1.533011 |
| C | -0.64054 | 5.289431 | -1.39239 |  |  |  |  |

Table S10. Calculated (TDDFT) values of the five lowest energy transition of **PDI**–**MeR**

| **PDI**–**MeBZ** | | | |
| --- | --- | --- | --- |
| No. | Calculated  transition energy (nm) | Oscillator  Strength  (f) | Orbital excitation contribution (%) |
| 1 | 525.1787 | 0.8637 | HOMO → LUMO (99%) |
| 2 | 469.9219 | 0.1375 | H-1 → LUMO (97%) |
| 3 | 376.7486 | 0.0045 | H-2 → LUMO (99%) |
| 4 | 365.9294 | 0.0217 | H-5 → LUMO (10%), H-4 → LUMO (82%) |
| 5 | 364.6487 | 0.001 | H-3 → LUMO (94%) |
|  | | | |
| **PDI**–**MeNP** | | | |
| No. | Calculated  transition energy (nm) | Oscillator  Strength  (f) | Orbital excitation contribution (%) |
| 1 | 531.6418 | 0.5988 | H-1 → LUMO (12%), HOMO → LUMO (87%) |
| 2 | 507.3418 | 0.2711 | H-1 → LUMO (88%), HOMO → LUMO (12%) |
| 3 | 463.8565 | 0.1354 | H-2 → LUMO (97%) |
| 4 | 400.5304 | 0.0014 | H-3 → LUMO (100%) |
| 5 | 364.3165 | 0.0227 | H-5 → LUMO (16%), H-4 → LUMO (83%) |
|  | | | |
| **PDI**–**MePY** | | | |
| No. | Calculated  transition energy (nm) | Oscillator  Strength  (f) | Orbital excitation contribution (%) |
| 1 | 619.0852 | 0.0038 | HOMO → LUMO (100%) |
| 2 | 524.823 | 0.842 | H-1 → LUMO (99%) |
| 3 | 467.4768 | 0.1504 | H-2 → LUMO (96%) |
| 4 | 423.8921 | 0.0001 | H-3 → LUMO (100%) |
| 5 | 366.2212 | 0.0028 | HOMO → L+2 (95%) |

Table S11. Calculated (TDDFT) values of the five lowest energy transition of [**PDI**–**MeR**]**^•−^**

| [**PDI**–**MeBZ**]**^•−^** | | | |
| --- | --- | --- | --- |
| No. | Calculated  transition energy (nm) | Oscillator  Strength  (f) | Orbital excitation contribution (%) |
| 1 | 850.5467  (D_0_ → D_1_**)** | 0.0404 | 168α (SOMO(α)) → 169α (LUMO(α)) (66%),  167β (HOMO(β)) → 168β (SUMO(β)) (34%) |
| 2 | 709.2917  (D_0_ → D_2_**)** | 0.0385 | 168α (SOMO(α)) → 170α (L+1(α)) (94%) |
| 3 | 644.2411  (D_0_ → D_3_**)** | 0.904 | 168α (SOMO(α)) → 169α (LUMO(α)) (32%),  167β (HOMO(β)) → 168β (SUMO(β)) (63%) |
| 4 | 514.7135 | 0.0834 | 167β (H-1(β)) → 168β (SUMO(β)) (87%) |
| 5 | 489.8044 | 0.0324 | 168α (SOMO(α)) → 171α (L+2(α)) (81%) |
|  | | | |
| [**PDI**–**MeNP**]**^•−^** | | | |
| No. | Calculated  transition energy (nm) | Oscillator  Strength  (f) | Orbital excitation contribution (%) |
| 1 | 850.7802  (D_0_ → D_1_**)** | 0.0394 | 181α (SOMO(α)) → 182α (LUMO(α)) (65%),  180β (HOMO(β)) → 181β (SUMO(β)) (34%) |
| 2 | 710.7555  (D_0_ → D_2_**)** | 0.0382 | 181α (SOMO(α)) → 183α (L+1(α)) (95%) |
| 3 | 644.509  (D_0_ → D_3_**)** | 0.8977 | 181α (SOMO(α)) → 182α (LUMO(α)) (32%),  180β (HOMO(β)) → 181β (SUMO(β)) (63%) |
| 4 | 572.1203 | 0.0003 | 181α (SOMO(α)) → 184α (L+2(α)) (100%) |
| 5 | 514.1799 | 0.0845 | 179β (H-1(β)) → 181β (SUMO(β)) (87%) |
|  | | | |
| [**PDI**–**MePY**]**^•−^** | | | |
| No. | Calculated  transition energy (nm) | Oscillator  Strength  (f) | Orbital excitation contribution (%) |
| 1 | 851.3059  (D_0_ → D_1_**)** | 0.036 | 200α (SOMO(α)) → 202α (L+1(α)) (65%),  199β (HOMO(β)) → 200β (SUMO(β)) (35%) |
| 2 | 803.1625  (D_0_ → D_2_**)** | 0 | 200α (SOMO(α)) → 201α (LUMO(α)) (100%) |
| 3 | 709.4135  (D_0_ → D_3_**)** | 0.0397 | 200α (SOMO(α)) → 203α (L+2(α)) (95%) |
| 4 | 645.8183  (D_0_ → D_4_**)** | 0.8716 | 200α (SOMO(α)) → 202α (L+1(α)) (33%),  199β (HOMO(β)) → 200β (SUMO(β)) (62%) |
| 5 | 596.0779 | 0 | 198α (H-1(α)) → 201α (LUMO(α)) (43%),  198β (H-1(β)) → 202β (L+1(β)) (46%) |

Table S12. Excited states calculated by TDDFT calculations of **PDI**–**MeBZ**

**PDI**–**MeBZ (B3LYP/6-31+G(d))**

Excited state symmetry could not be determined.

Excited State	1: Singlet-?Sym	2.3608 eV 525.18 nm f=0.8637 <S**2>=0.000

157 ->168	-0.01661

157 ->170	0.02460

158 ->169	0.02045

160 ->169	-0.01343

162 ->168	-0.02518

162 ->170	0.01958

162 ->171	-0.02490

163 ->168	-0.02997

163 ->170	0.03922

166 ->168	-0.01869

166 ->169	-0.02569

166 ->172	-0.04574

166 ->181	0.01055

167 ->168	0.70313

167 ->170	-0.01445

167 ->171	0.01049

This state for optimization and/or second-order correction.

Total Energy, E(TD-HF/TD-DFT) = -2048.74000507

Copying the excited state density for this state as the 1-particle RhoCI density.

Excited state symmetry could not be determined.

Excited State	2: Singlet-?Sym	2.6384 eV 469.93 nm f=0.1375 <S**2>=0.000

149 ->168	-0.01230

150 ->168	0.01098

154 ->168	0.01293

157 ->169	0.01046

158 ->168	-0.03790

158 ->170	-0.01073

158 ->171	-0.02495

160 ->171	0.01839

161 ->171	-0.01337

162 ->181	-0.01005

163 ->169	0.03913

166 ->168	0.69565

166 ->170	0.01172

166 ->171	0.01736

167 ->168	0.01871

167 ->169	0.04863

167 ->172	0.08778

167 ->173	0.01173

167 ->181	-0.01137

Excited state symmetry could not be determined.

Excited State	3: Singlet-?Sym	3.2909 eV 376.75 nm f=0.0045 <S**2>=0.000

137 ->168	-0.01277

158 ->168	-0.01726

160 ->168	-0.01881

161 ->168	-0.01372

162 ->168	0.02898

163 ->169	0.01202

165 ->168	0.70303

165 ->169	-0.01104

165 ->170	0.02777

167 ->169	0.02113

167 ->171	0.01545

167 ->172	0.03112

Excited state symmetry could not be determined.

Excited State	4: Singlet-?Sym	3.3882 eV 365.93 nm f=0.0217 <S**2>=0.000

149 ->169	-0.01116

155 ->168	-0.02493

156 ->168	-0.01769

157 ->168	0.04001

158 ->169	-0.01816

160 ->168	-0.02829

161 ->168	-0.02195

161 ->169	0.04838

162 ->168	0.22829

163 ->168	0.64128

164 ->168	0.16151

165 ->168	-0.01392

166 ->172	-0.01160

167 ->168	0.03601

Excited state symmetry could not be determined.

Excited State	5: Singlet-?Sym	3.4001 eV 364.64 nm f=0.0010 <S**2>=0.000

161 ->169	-0.01147

162 ->168	-0.05054

163 ->168	-0.15173

164 ->168	0.68710

164 ->169	-0.01034

164 ->170	0.02685

167 ->169	-0.02302

Table S13. Excited states calculated by TDDFT calculations of **PDI**–**MeNP**

**PDI**–**MeNP (B3LYP/6-31+G(d))**

Excited state symmetry could not be determined.

Excited State	1: Singlet-?Sym	2.3321 eV 531.65 nm f=0.5988 <S**2>=0.000

168 -> 181	0.01517

168 -> 183	-0.02025

171 -> 182	0.01658

174 -> 182	0.01327

175 -> 181	-0.01803

175 -> 183	0.01665

175 -> 185	-0.02104

176 -> 181	-0.02488

176 -> 183	0.03340

177 -> 181	-0.01155

178 -> 182	0.02066

178 -> 186	-0.03814

179 -> 181	-0.24637

179 -> 183	-0.01321

180 -> 181	0.65996

This state for optimization and/or second-order correction.

Total Energy, E(TD-HF/TD-DFT) = -2202.39812399

Copying the excited state density for this state as the 1-particle RhoCI density.

Excited state symmetry could not be determined.

Excited State	2: Singlet-?Sym	2.4438 eV 507.35 nm f=0.2711 <S**2>=0.000

167 -> 181	0.01412

168 -> 183	-0.01392

171 -> 182	0.01051

175 -> 181	-0.01802

175 -> 183	0.01192

175 -> 185	-0.01444

176 -> 181	-0.01488

176 -> 183	0.02261

178 -> 182	0.01463

178 -> 186	-0.02515

179 -> 181	0.66164

179 -> 183	0.01702

180 -> 181	0.24435

180 -> 183	-0.01223

Excited state symmetry could not be determined.

Excited State	3: Singlet-?Sym	2.6729 eV 463.85 nm f=0.1354 <S**2>=0.000

158 -> 181	0.01485

166 -> 181	0.01327

166 -> 183	0.01016

171 -> 181	0.04007

171 -> 183	0.01051

171 -> 185	0.02433

174 -> 185	0.02616

176 -> 182	-0.04085

177 -> 184	0.01036

178 -> 181	0.69497

178 -> 183	0.01027

178 -> 185	0.01779

179 -> 186	0.02051

180 -> 182	-0.05042

180 -> 186	0.08941

180 -> 187	-0.01614

Excited state symmetry could not be determined.

Excited State	4: Singlet-?Sym	3.0955 eV 400.53 nm f=0.0014 <S**2>=0.000

163 -> 181	0.01545

175 -> 181	-0.01078

177 -> 181	0.70620

177 -> 183	0.02202

179 -> 181	-0.01322

Excited state symmetry could not be determined.

Excited State	5: Singlet-?Sym	3.4032 eV 364.31 nm f=0.0227 <S**2>=0.000

158 -> 182	-0.01241

166 -> 182	-0.01128

168 -> 181	-0.04762

169 -> 181	0.01099

171 -> 182	-0.02510

174 -> 182	0.03672

175 -> 181	0.28017

176 -> 181	0.64307

178 -> 186	-0.01243

180 -> 181	0.03558

Table S14. Excited states calculated by TDDFT calculations of **PDI**–**MePY**

**PDI**–**MePY (B3LYP/6-31+G(d))**

Excited state symmetry could not be determined.

Excited State	1: Singlet-?Sym	2.0027 eV 619.09 nm f=0.0038 <S**2>=0.000

188 -> 200	0.01430

195 -> 200	-0.01214

198 -> 200	0.02868

199 -> 200	0.70559

199 -> 202	0.01048

199 -> 203	-0.02117

This state for optimization and/or second-order correction.

Total Energy, E(TD-HF/TD-DFT) = -2432.28660581

Copying the excited state density for this state as the 1-particle RhoCI density.

Excited state symmetry could not be determined.

Excited State	2: Singlet-?Sym	2.3624 eV 524.82 nm f=0.8420 <S**2>=0.000

186 -> 200	0.01482

186 -> 203	0.02188

187 -> 203	0.01272

189 -> 202	0.01946

192 -> 202	0.01375

193 -> 200	-0.02518

193 -> 203	-0.02083

193 -> 205	-0.02474

194 -> 200	-0.02858

194 -> 203	-0.03853

197 -> 202	-0.02532

197 -> 206	-0.04571

198 -> 200	0.70287

198 -> 203	0.01362

199 -> 200	-0.02836

Excited state symmetry could not be determined.

Excited State	3: Singlet-?Sym	2.6522 eV 467.48 nm f=0.1504 <S**2>=0.000

177 -> 200	-0.01473

183 -> 200	0.01250

189 -> 200	-0.03715

189 -> 203	0.01043

189 -> 205	-0.02397

192 -> 205	-0.02161

194 -> 202	0.03895

196 -> 200	0.01806

197 -> 200	0.69441

197 -> 203	-0.01092

197 -> 205	0.01711

198 -> 202	0.04966

198 -> 206	0.08905

199 -> 201	-0.03510

199 -> 206	-0.01148

Excited state symmetry could not be determined.

Excited State	4: Singlet-?Sym	2.9249 eV 423.89 nm f=0.0001 <S**2>=0.000

184 -> 200	0.02318

195 -> 200	-0.01005

196 -> 200	0.70570

196 -> 202	0.01364

196 -> 203	-0.02097

197 -> 200	-0.01999

Excited state symmetry could not be determined.

Excited State	5: Singlet-?Sym	3.3855 eV 366.22 nm f=0.0028 <S**2>=0.000

188 -> 202	0.01321

189 -> 200	0.02041

192 -> 200	0.02450

193 -> 200	-0.04699

194 -> 200	-0.11357

195 -> 200	0.04206

195 -> 202	-0.01059

197 -> 200	0.01080

197 -> 202	-0.01545

198 -> 200	-0.01025

198 -> 202	-0.05822

198 -> 203	0.01110

199 -> 201	-0.03809

199 -> 202	0.68791

199 -> 203	-0.02048

199 -> 206	-0.02896

199 -> 208	0.02025

Table S15. Excited states calculated by TDDFT calculations of [**PDI**–**MeBZ**]**^•−^**

[**PDI**–**MeBZ**]**^•−^ (B3LYP/6-31+G(d))**

Excited state symmetry could not be determined.

Excited State	1: 2.006-?Sym	1.4577 eV 850.57 nm f=0.0404 <S**2>=0.756

155A ->170A	-0.02199

158A ->169A	-0.01153

158A ->175A	0.01213

166A ->175A	-0.01064

167A ->171A	0.02109

167A ->178A	-0.01010

167A ->179A	-0.01203

168A ->169A	0.81041

168A ->187A	0.01444

157B ->168B	0.01059

157B ->170B	0.02819

158B ->176B	-0.01474

162B ->168B	-0.01812

163B ->169B	0.01228

165B ->170B	-0.02283

166B ->174B	-0.01679

167B ->168B	0.58370

167B ->172B	0.01028

This state for optimization and/or second-order correction.

Total Energy, E(TD-HF/TD-DFT) = -2048.90824140

Copying the excited state density for this state as the 1-particle RhoCI density.

Excited state symmetry could not be determined.

Excited State	2: 2.020-?Sym	1.7480 eV 709.29 nm f=0.0385 <S**2>=0.770

164A ->169A	0.01721

165A ->169A	-0.02012

167A ->174A	0.03340

167A ->175A	-0.04640

168A ->169A	0.05111

168A ->170A	0.97201

168A ->171A	0.02853

168A ->205A	0.01008

153B ->168B	-0.01710

154B ->168B	-0.01138

155B ->169B	0.01213

156B ->168B	0.01150

156B ->169B	-0.01156

157B ->169B	0.02987

158B ->168B	0.05330

158B ->181B	-0.01364

162B ->169B	-0.01224

162B ->189B	0.01471

163B ->168B	-0.01322

163B ->172B	0.02199

164B ->169B	0.01934

165B ->169B	0.01636

166B ->168B	0.18329

166B ->181B	0.01079

167B ->168B	-0.06310

167B ->174B	0.03756

167B ->176B	-0.03699

Excited state symmetry could not be determined.

Excited State	3: 2.061-?Sym	1.9245 eV 644.25 nm f=0.9040 <S**2>=0.812

151A ->175A	0.01490

153A ->175A	0.01487

154A ->174A	0.02001

155A ->170A	-0.03929

155A ->171A	0.01808

157A ->170A	-0.01622

158A ->169A	-0.03259

158A ->174A	0.01029

158A ->187A	-0.01847

159A ->169A	-0.02749

159A ->187A	-0.01712

160A ->169A	-0.01251

160A ->170A	0.02803

162A ->170A	-0.01978

163A ->170A	0.01086

164A ->170A	0.07504

165A ->170A	-0.07109

165A ->171A	0.01084

165A ->205A	-0.01180

166A ->174A	-0.01146

166A ->175A	0.01251

167A ->170A	-0.01974

167A ->171A	-0.02807

167A ->178A	0.02373

167A ->179A	0.03036

168A ->169A	-0.56598

168A ->170A	0.08011

168A ->172A	0.02778

168A ->174A	-0.05653

168A ->175A	-0.02127

168A ->187A	-0.01142

133B ->189B	-0.01002

152B ->181B	-0.01075

153B ->176B	0.01722

154B ->174B	-0.01966

154B ->189B	-0.01135

157B ->172B	0.02970

158B ->169B	-0.01199

159B ->172B	0.01181

161B ->169B	0.01204

161B ->172B	-0.01286

162B ->168B	0.02735

162B ->169B	0.01520

162B ->172B	-0.03865

163B ->169B	0.08734

163B ->174B	-0.01584

163B ->189B	-0.02205

164B ->168B	-0.02524

164B ->172B	0.03162

166B ->168B	0.01407

166B ->169B	0.04146

166B ->171B	0.01659

166B ->174B	0.08752

166B ->189B	-0.01448

167B ->168B	0.79089

167B ->172B	-0.03471

167B ->181B	0.02096

Excited state symmetry could not be determined.

Excited State	4: 2.030-?Sym	2.4088 eV 514.70 nm f=0.0834 <S**2>=0.780

149A ->169A	-0.01014

154A ->171A	0.01507

154A ->179A	0.01128

155A ->174A	0.01021

155A ->187A	-0.01254

158A ->170A	0.01708

158A ->171A	0.01504

159A ->169A	0.01174

159A ->171A	0.01306

160A ->169A	-0.02553

162A ->169A	0.02318

164A ->169A	-0.03643

165A ->169A	0.02039

166A ->178A	0.02409

166A ->179A	0.03272

167A ->169A	-0.03565

167A ->172A	0.01529

167A ->174A	-0.07751

168A ->170A	-0.16022

168A ->171A	-0.27072

168A ->172A	0.03323

168A ->173A	-0.01155

168A ->178A	-0.01360

168A ->179A	-0.02094

168A ->201A	-0.01099

168A ->205A	0.01889

154B ->170B	0.02556

154B ->172B	0.01010

158B ->168B	-0.02598

162B ->169B	-0.02043

162B ->174B	0.01156

163B ->168B	0.03285

163B ->172B	-0.02603

165B ->169B	-0.02900

166B ->168B	0.93422

166B ->170B	0.02335

166B ->172B	-0.02521

166B ->181B	0.02015

167B ->171B	-0.01155

167B ->174B	-0.04988

167B ->189B	0.01158

Excited state symmetry could not be determined.

Excited State	5: 2.174-?Sym	2.5313 eV 489.80 nm f=0.0324 <S**2>=0.931

149A ->174A	0.01362

150A ->174A	-0.01149

155A ->169A	-0.01924

155A ->175A	0.01294

158A ->170A	0.01745

158A ->179A	0.01061

159A ->169A	0.01628

159A ->170A	0.03440

159A ->171A	0.01342

160A ->169A	-0.02753

160A ->170A	0.01618

162A ->169A	0.02860

164A ->169A	-0.03128

166A ->171A	0.04443

167A ->169A	0.12917

167A ->174A	0.03902

167A ->175A	0.03658

167A ->187A	-0.01550

168A ->170A	-0.07271

168A ->171A	0.89889

168A ->172A	-0.09673

168A ->173A	0.03651

168A ->174A	-0.01107

168A ->178A	0.02214

168A ->179A	0.03491

150B ->174B	-0.01922

151B ->174B	-0.01339

152B ->174B	0.02092

153B ->168B	0.01673

153B ->170B	0.02394

154B ->168B	0.01122

154B ->172B	-0.02693

157B ->169B	0.01742

157B ->176B	0.02000

159B ->169B	0.01071

161B ->168B	-0.01820

161B ->169B	-0.01270

162B ->168B	-0.02210

162B ->169B	-0.03305

163B ->168B	-0.11599

163B ->170B	-0.02104

163B ->172B	-0.01657

164B ->169B	0.02283

165B ->169B	-0.02344

165B ->176B	-0.01431

166B ->168B	0.25838

166B ->170B	0.01517

166B ->172B	0.02648

166B ->181B	-0.01785

167B ->169B	-0.21738

167B ->174B	-0.06843

167B ->176B	0.02195

167B ->189B	-0.01233

Table S16. Excited states calculated by TDDFT calculations of [**PDI**–**MeNP**]**^•−^**

[**PDI**–**MeNP**]**^•−^ (B3LYP/6-31+G(d))**

Excited state symmetry could not be determined.

Excited State	1: 2.006-?Sym	1.4573 eV 850.76 nm f=0.0394 <S**2>=0.756

168A -> 183A	-0.02301

171A -> 182A	-0.01068

171A -> 188A	0.01534

178A -> 188A	-0.01067

180A -> 186A	0.02117

180A -> 193A	-0.01018

181A -> 182A	0.80898

181A -> 201A	0.01538

168B -> 184B	0.02917

169B -> 184B	-0.01150

171B -> 189B	-0.01359

174B -> 181B	0.02316

175B -> 182B	0.01271

176B -> 184B	-0.02314

179B -> 187B	0.01684

180B -> 181B	0.58565

180B -> 186B	-0.01033

This state for optimization and/or second-order correction.

Total Energy, E(TD-HF/TD-DFT) = -2202.56585418

Copying the excited state density for this state as the 1-particle RhoCI density.

Excited state symmetry could not be determined.

Excited State	2: 2.020-?Sym	1.7444 eV 710.75 nm f=0.0382 <S**2>=0.770

168A -> 182A	-0.01044

176A -> 182A	0.02709

180A -> 187A	0.03316

180A -> 188A	-0.04630

181A -> 182A	-0.03517

181A -> 183A	0.97419

181A -> 186A	0.02894

165B -> 181B	0.01683

166B -> 181B	-0.01133

168B -> 182B	0.03239

169B -> 182B	-0.01329

171B -> 181B	0.05485

171B -> 196B	0.01104

174B -> 182B	0.01655

174B -> 204B	0.01670

175B -> 181B	-0.01545

175B -> 186B	-0.02186

176B -> 182B	0.02171

179B -> 181B	0.18141

179B -> 196B	-0.01035

180B -> 181B	0.04299

180B -> 187B	-0.03848

180B -> 188B	0.01768

180B -> 189B	-0.03246

Excited state symmetry could not be determined.

Excited State	3: 2.061-?Sym	1.9237 eV 644.51 nm f=0.8977 <S**2>=0.812

159A -> 188A	-0.01295

165A -> 188A	-0.01595

166A -> 187A	0.02007

168A -> 183A	-0.04170

168A -> 186A	0.01852

171A -> 182A	-0.02833

171A -> 187A	0.01042

171A -> 201A	-0.01676

172A -> 182A	-0.03529

172A -> 201A	-0.02207

173A -> 183A	0.02970

173A -> 186A	0.01057

176A -> 183A	0.10600

178A -> 187A	-0.01123

178A -> 188A	0.01259

180A -> 183A	-0.01837

180A -> 186A	-0.02849

180A -> 191A	-0.02171

180A -> 192A	-0.01186

180A -> 193A	0.02596

180A -> 194A	-0.01470

181A -> 182A	-0.56930

181A -> 183A	-0.05513

181A -> 184A	-0.01077

181A -> 185A	0.01847

181A -> 187A	-0.05860

181A -> 188A	-0.02236

181A -> 201A	-0.01173

164B -> 186B	-0.01376

164B -> 196B	0.01236

165B -> 189B	-0.01523

166B -> 187B	0.01963

168B -> 186B	-0.02800

169B -> 186B	0.01158

174B -> 181B	-0.03548

174B -> 182B	0.02512

174B -> 186B	-0.05224

175B -> 181B	0.01091

175B -> 182B	0.08649

175B -> 186B	0.01497

175B -> 187B	0.01684

175B -> 204B	0.01997

176B -> 181B	-0.01319

177B -> 186B	0.01153

179B -> 182B	0.04119

179B -> 187B	-0.08884

179B -> 204B	0.01285

180B -> 181B	0.79079

180B -> 186B	0.03539

180B -> 195B	-0.01150

180B -> 196B	-0.01831

Excited state symmetry could not be determined.

Excited State	4: 2.012-?Sym	2.1671 eV 572.13 nm f=0.0003 <S**2>=0.762

171A -> 184A	0.01372

178A -> 184A	-0.02909

181A -> 184A	0.99881

181A -> 185A	0.01089

181A -> 190A	0.02162

181A -> 192A	-0.01236

Excited state symmetry could not be determined.

Excited State	5: 2.030-?Sym	2.4113 eV 514.19 nm f=0.0845 <S**2>=0.780

163A -> 182A	-0.01232

166A -> 186A	0.01494

168A -> 187A	0.01039

168A -> 201A	-0.01327

171A -> 183A	0.01589

171A -> 186A	0.01329

172A -> 183A	0.01396

172A -> 186A	0.01676

173A -> 182A	-0.03720

173A -> 187A	0.01247

173A -> 201A	-0.01660

176A -> 182A	-0.04125

178A -> 191A	-0.02220

178A -> 192A	-0.01207

178A -> 193A	0.02743

178A -> 194A	-0.01596

180A -> 182A	-0.03679

180A -> 187A	-0.07856

181A -> 183A	-0.15810

181A -> 186A	-0.27282

181A -> 191A	0.01135

181A -> 193A	-0.01728

181A -> 194A	0.01438

181A -> 219A	-0.01153

181A -> 220A	0.01213

166B -> 184B	0.02567

166B -> 186B	-0.01014

171B -> 181B	-0.02556

174B -> 182B	0.02793

174B -> 187B	0.01589

175B -> 181B	0.03238

175B -> 186B	0.02570

176B -> 182B	-0.02384

179B -> 181B	0.93455

179B -> 184B	0.02301

179B -> 186B	0.02589

179B -> 195B	-0.01134

179B -> 196B	-0.01766

180B -> 187B	0.05074

180B -> 204B	-0.01039

Table S17. Excited states calculated by TDDFT calculations of [**PDI**–**MePY**]**^•−^**

[**PDI**–**MePY**]**^•−^ (B3LYP/6-31+G(d))**

Excited state symmetry could not be determined.

Excited State	1: 2.006-?Sym	1.4564 eV 851.33 nm f=0.0360 <S**2>=0.756

186A -> 203A	0.02288

189A -> 202A	0.01043

189A -> 208A	0.01264

197A -> 208A	0.01051

199A -> 205A	0.01484

199A -> 206A	0.01397

200A -> 202A	0.80640

188B -> 203B	-0.03046

190B -> 210B	0.01249

193B -> 200B	0.02132

194B -> 200B	-0.01074

194B -> 202B	-0.01150

195B -> 203B	0.02276

197B -> 207B	-0.01615

198B -> 200B	0.02085

199B -> 200B	0.58883

199B -> 206B	-0.01017

This state for optimization and/or second-order correction.

Total Energy, E(TD-HF/TD-DFT) = -2432.44147006

Copying the excited state density for this state as the 1-particle RhoCI density.

Excited state symmetry could not be determined.

Excited State	2: 2.013-?Sym	1.5437 eV 803.18 nm f=0.0000 <S**2>=0.763

189A -> 201A	-0.01234

197A -> 201A	-0.02877

198A -> 201A	-0.01068

200A -> 201A	0.99840

200A -> 202A	-0.01484

200A -> 203A	0.01539

200A -> 205A	0.01011

200A -> 206A	-0.01764

200A -> 209A	0.02324

200A -> 227A	0.01069

Excited state symmetry could not be determined.

Excited State	3: 2.020-?Sym	1.7477 eV 709.41 nm f=0.0397 <S**2>=0.770

186A -> 202A	0.01024

195A -> 202A	-0.02658

198A -> 208A	-0.01153

199A -> 207A	-0.03200

199A -> 208A	-0.04477

200A -> 201A	-0.01740

200A -> 202A	-0.04131

200A -> 203A	0.97284

200A -> 205A	-0.02259

200A -> 206A	-0.01750

200A -> 242A	0.01087

182B -> 200B	0.01684

184B -> 200B	0.01126

188B -> 202B	-0.03322

190B -> 200B	0.05433

190B -> 216B	-0.01364

193B -> 202B	-0.01788

193B -> 206B	-0.01287

193B -> 225B	-0.01332

193B -> 226B	-0.01022

194B -> 206B	-0.01917

195B -> 202B	-0.02121

197B -> 200B	-0.17957

197B -> 216B	-0.01184

198B -> 200B	-0.04457

199B -> 200B	0.04788

199B -> 207B	-0.03796

199B -> 209B	-0.02225

199B -> 210B	0.02992

Excited state symmetry could not be determined.

Excited State	4: 2.060-?Sym	1.9198 eV 645.82 nm f=0.8716 <S**2>=0.811

182A -> 208A	-0.01638

184A -> 207A	0.01919

186A -> 203A	0.04028

186A -> 205A	0.01385

186A -> 206A	0.01190

187A -> 203A	0.01342

189A -> 202A	0.02863

189A -> 207A	-0.01015

189A -> 221A	0.01109

189A -> 222A	-0.01149

190A -> 202A	0.01242

191A -> 202A	0.03127

191A -> 221A	0.01262

191A -> 222A	-0.01360

192A -> 203A	0.02999

193A -> 203A	0.01723

195A -> 203A	-0.10345

195A -> 242A	-0.01314

197A -> 207A	-0.01087

197A -> 208A	-0.01263

199A -> 203A	0.01898

199A -> 205A	-0.02003

199A -> 206A	-0.01890

199A -> 212A	0.02271

199A -> 213A	0.02375

199A -> 215A	-0.01368

200A -> 201A	-0.01385

200A -> 202A	-0.57222

200A -> 203A	-0.06261

200A -> 204A	-0.01097

200A -> 207A	-0.05800

200A -> 208A	0.02263

181B -> 206B	0.01312

181B -> 216B	0.01500

182B -> 209B	-0.01004

182B -> 210B	0.01383

184B -> 207B	-0.01915

188B -> 206B	-0.02898

190B -> 202B	0.01348

192B -> 202B	0.01369

193B -> 200B	-0.03253

193B -> 202B	-0.04100

193B -> 206B	-0.04894

193B -> 216B	0.01099

194B -> 200B	0.01988

194B -> 202B	-0.07811

194B -> 206B	0.02453

194B -> 207B	0.01458

194B -> 225B	-0.01370

194B -> 226B	-0.01200

195B -> 200B	-0.01126

197B -> 202B	0.03991

197B -> 207B	0.08719

197B -> 225B	0.01097

198B -> 200B	0.01604

198B -> 207B	0.01684

199B -> 200B	0.78806

199B -> 206B	0.03469

199B -> 216B	0.02220

Excited state symmetry could not be determined.

Excited State	5: 3.470-?Sym	2.0800 eV 596.08 nm f=0.0000 <S**2>=2.761

169A -> 239A	0.01188

169A -> 283A	-0.01126

170A -> 239A	-0.01437

170A -> 277A	-0.01104

170A -> 283A	0.01358

170A -> 285A	-0.01085

183A -> 206A	0.01044

183A -> 223A	-0.01057

183A -> 227A	-0.05779

183A -> 228A	-0.01920

183A -> 229A	0.01034

183A -> 231A	0.01004

184A -> 209A	0.01124

184A -> 227A	-0.01397

185A -> 209A	0.09638

185A -> 210A	-0.02137

185A -> 242A	0.01008

185A -> 243A	-0.01375

185A -> 248A	-0.01022

189A -> 205A	-0.02585

189A -> 206A	0.02955

190A -> 205A	0.06672

190A -> 206A	-0.07654

190A -> 227A	0.01815

196A -> 204A	0.12271

197A -> 201A	0.05045

198A -> 201A	-0.65713

199A -> 201A	0.19402

200A -> 201A	-0.01023

169B -> 239B	-0.01375

169B -> 277B	-0.01045

169B -> 282B	0.01028

169B -> 283B	-0.01093

170B -> 239B	0.01189

183B -> 205B	0.01298

183B -> 222B	0.01185

183B -> 225B	-0.01382

183B -> 227B	0.05757

183B -> 228B	0.01954

183B -> 229B	-0.01220

183B -> 231B	-0.01022

184B -> 208B	-0.01461

185B -> 208B	-0.09680

185B -> 209B	0.01181

185B -> 210B	0.01556

185B -> 242B	-0.01496

189B -> 205B	0.10743

189B -> 206B	-0.01353

189B -> 227B	0.01855

196B -> 204B	-0.12276

197B -> 201B	-0.09530

198B -> 201B	0.67761

199B -> 201B	-0.06565

 Figure S33. Excited state hole (blue) and electron (lime) distributions with *D* index values of (A) [**PDI**–**MeBZ**]**^•−^**, (B) [**PDI**–**MeNP**]**^•−^**, and (C) [**PDI–MePY**]**^•−^** for the D_0_ → D_1_ and D_0_ → D_2_ transitions.

**(C)**

**(B)**

**(A)**

**(A)**

**(B)**

Figure S34. Current strength susceptibilities and pathways for [**PDI**–**MeR**]**^•−^** (B3LYP/6-31G(d)). The bond current strength were calculated by numerical integration of the current flow passing through cut planes (4.4 × 10.0 bohr) perpendicularly to the selected bonds. The selected bonds were integrated counterclockwise with the magnetic field oriented along +Z: negative and positive values correspond to clockwise (diatropic) and counterclockwise (paratropic) currents, respectively. (A) Selected bonds (a – r), (B) Representative cut plane perpendicularly to the bond a (top-down and side views).

Table S18. The induced current strengths (nA / T) of selected bonds (a – r) in [**PDI**–**MeBZ**]**^•−^** at the B3LYP/6-31G(d) level.

| Bond | a | b | c | d | e | f |
| --- | --- | --- | --- | --- | --- | --- |
| Induced current | 1.3 | -0.3 | -8.5 | -9.0 | -6.1 | -5.6 |
| Bond | g | h | i | j | k | l |
| Induced current | -8.4 | 8.4 | 8.6 | 9.0 | -8.7 | -8.4 |
| Bond | m | n | o | p | q | r |
| Induced current | 8.4 | 5.7 | 6.1 | 8.9 | 8.4 | 0.4 |

Table S19. The induced current strengths (nA / T) of selected bonds (a – r) in [**PDI**–**MeNP**]**^•−^** at the B3LYP/6-31G(d) level.

| Bond | a | b | c | d | e | f |
| --- | --- | --- | --- | --- | --- | --- |
| Induced current | -1.3 | -0.4 | 8.4 | -8.9 | -6.1 | -5.7 |
| Bond | g | h | i | j | k | l |
| Induced current | -8.5 | -8.4 | 8.6 | -9.0 | -8.6 | -8.4 |
| Bond | m | n | o | p | q | r |
| Induced current | 8.4 | 5.7 | 6.1 | 8.9 | 8.4 | 0.4 |

Table S20. The induced current strengths (nA / T) of selected bonds (a – r) in [**PDI**–**MePY**]**^•−^** at the B3LYP/6-31G(d) level.

| Bond | a | b | c | d | e | f |
| --- | --- | --- | --- | --- | --- | --- |
| Induced current | -1.2 | -0.4 | 7.8 | -8.7 | -5.9 | -5.6 |
| Bond | g | h | i | j | k | l |
| Induced current | -8.4 | -8.3 | 8.5 | -9.0 | -8.7 | -8.5 |
| Bond | m | n | o | p | q | r |
| Induced current | 8.5 | 5.8 | 6.2 | 9.0 | 8.6 | 0.2 |

**References**

[1]	R. El-Berjawi, P. Hudhomme, “Synthesis of a perylenediimide-fullerene C_60_ dyad: A simple use of a nitro leaving group for a Suzuki-Miyaura coupling reaction”, *Dyes and Pigments* 159 (2018): 551-556, https://doi.org/10.1016/j.dyepig.2018.07.037

[2]	R. K. Gupta, S. K. Pathak, B. Pradhan, et al., “Self-assembly of luminescent *N*-annulated perylene tetraesters into fluid columnar phases”, *Soft Matter* 11 (2015): 3629-3636, https://doi.org/10.1039/C5SM00463B

[3]	H. Borchert 2014, in *Solar Cells Based on Colloidal Nanocrystals*. Switzerland: Springer Cham.

[4]	K. J. Lee, N. Elgrishi, B. Kandemir, J. L. Dempsey, “Electrochemical and spectroscopic methods for evaluating molecular electrocatalysts”, *Nature Reviews Chemistry* 1 (2017): 0039, https://doi.org/10.1038/s41570-017-0039

[5]	X. Li, K. Perera, J. He, A. Gumyusenge, J. Mei, “Solution-processable electrochromic materials and devices: roadblocks and strategies towards large-scale applications”, *Journal of Materials Chemistry C* 7 (2019): 12761-12789, https://doi.org/10.1039/C9TC02861G

[6]	Y. Zhuang, M. Zhu, Q. Zhang, et al., “Electrochromic/Electrofluorochromic Devices based on Terpyridine‐Attached Viologen Derivatives”, *Advanced Materials Technologies* 9 (2024): 2302080, https://doi.org/10.1002/admt.202302080

[7]	H. Schlegel, G. Scuseria, M. Robb, et al., “Gaussian 16, revision C. 01”, *Gaussian Inc.: Wallingford, CT, USA* (2016):

[8]	Z. Liu, T. Lu, Q. Chen, “An sp-hybridized all-carboatomic ring, cyclo [18] carbon: Electronic structure, electronic spectrum, and optical nonlinearity”, *Carbon* 165 (2020): 461-467, https://doi.org/10.1016/j.carbon.2020.05.023

[9]	T. Lu, F. Chen, “Multiwfn: A multifunctional wavefunction analyzer”, *Journal of computational chemistry* 33 (2012): 580-592, https://doi.org/10.1002/jcc.22885

[10]	W. Humphrey, A. Dalke, K. Schulten, “VMD: visual molecular dynamics”, *Journal of molecular graphics* 14 (1996): 33-38, https://doi.org/10.1016/0263-7855(96)00018-5

[11]	D. Geuenich, K. Hess, F. Köhler, R. Herges, “Anisotropy of the induced current density (ACID), a general method to quantify and visualize electronic delocalization”, *Chemical Reviews* 105 (2005): 3758-3772, https://doi.org/10.1021/cr0300901

[12]	J. Jusélius, D. Sundholm, J. Gauss, “Calculation of current densities using gauge-including atomic orbitals”, *The Journal of chemical physics* 121 (2004): 3952-3963, https://doi.org/10.1063/1.1773136

[13]	Povray, Persistence of vision raytracer, https://www.povray.org/, accessed: 4, September.

[14]	U. Ayachit 2015, in *The paraview guide: a parallel visualization application*. Clifton Park, NY, USA: Kitware, Inc. 1930934300.
