# Supplementary material for: Aromaticity‐Engineered Open‐Shell Radical Anions for Air‐Stable Doublet Emission in N‐Annulated Perylene Diimide
Source: Adv Sci (Weinh). 2026 Jun 15:e75858. Online ahead of print. doi: 10.1002/advs.75858 (PMC13335971; doi:10.1002/advs.75858)
Supplement: Supplementary file 1 — Supporting File 1: advs75858‐sup‐0001‐SuppMat.docx. [file ADVS-9999-e75858-s003.docx]

Supporting Information

Aromaticity-Engineered Open-Shell Radical Anions for Air-Stable Doublet Emission in *N*-Annulated Perylene Diimide

Jeongyoon Kim, Soyoon Lee, Min-Ji Kim, Mina Ahn* and Kyung-Ryang Wee*

| Contents | | Page |
| --- | --- | --- |
| General Methods | | S3–S4 |
| Synthetic procedures and compound data | | S5–S7 |
| Figure S1–S13. ^1^H-NMR and ^13^C{^1^H}-NMR spectra of **PDI**–**NO_2_**, **PDIN**–**H**,  **PDI**–**MeR**, [**PDI**–**MeR**]**^•−^** | | S8–S14 |
| Figure S14. GC-MS data of **PDI**–**MeR** | | S15 |
| Figure S15. ICP–OES spectra of **PDI**–**MeR** with characteristic cobalt emission lines | | S16 |
| Figure S16. Cyclic voltammogram for the oxidation and reduction of **Cobaltocene** | | S17 |
| Table S1. Photophysical parameters of synthesized **PDI**–**MeR** and [**PDI–MeR**]^•−^ | | S18 |
| Table S2. CV data for the observed reduction wave of **PDI**–**MeR** in DCM | | S18 |
| Table S3. CV data for the observed reduction wave of **PDI**–**MeR** in DMF | | S18 |
| Figure S17. UV-vis absorption/ fluorescence spectra of **PDI**–**MeR** in DMF | | S19 |
| Figure S18. UV-vis absorption/ fluorescence spectra of **PDI**–**MeR** in solvents | | S20 |
| Figure S19. Cyclic voltammogram for the oxidation and reduction of **PDI**–**MeR** | | S21 |
| Figure S20. Cyclic voltammogram for the reduction of **PDI**–**MeR** over 200 cycles | | S22 |
| Figure S21. CW EPR spectra of [**PDI**–**MeR**]**^•−^** in DMF | | S23 |
| Figure S22. CV comparison of **PDI**–**MeR** and [**PDI**–**MeR**]**^•−^** in DMF | | S24 |
| Figure S23. UV-vis absorption spectral change of **PDI**–**MeR** to [**PDI**–**MeR**]**^•−^** | | S25 |
| Figure S24. Doublet emission stability of chemically generated [**PDI**–**MeR**]**^•−^** | | S26 |
| Calculation Details | S27 | |
| Figure S25. Spin density maps of [**PDI**–**MeR**]**^•−^** radical anion states | S28 | |
| Figure S26. AICDplots of **PDI**–**MeR** and [**PDI**–**MeR**]**^•−^ (**R = BZ, NP) | S29 | |
| Figure S27-S32. Energy levels and electronic transitions | S30–S35 | |
| Table S4. Cartesian coordinates for optimized structure for **PDI**–**MeBZ** | S36 | |
| Table S5. Cartesian coordinates for optimized structure for [**PDI**–**MeBZ**]**^•−^** | S37 | |
| Table S6. Cartesian coordinates for optimized structure for **PDI**–**MeNP** | S38 | |
| Table S7. Cartesian coordinates for optimized structure for [**PDI**–**MeNP**]**^•−^** | S39 | |
| Table S8. Cartesian coordinates for optimized structure for **PDI**–**MePY** | S40 | |
| Table S9. Cartesian coordinates for optimized structure for [**PDI**–**MePY**]**^•−^** | S41 | |
| Table S10. TDDFT values of the five lowest energy transition of **PDI**–**MeR** | S42 | |
| Table S11. TDDFT values of the five lowest energy transition of [**PDI**–**MeR**]**^•−^** | S43 | |
| Table S12. Excited states calculated by TDDFT Calculations of **PDI**–**MeBZ** | S44–S45 | |
| Table S13. Excited states calculated by TDDFT Calculations of **PDI**–**MeNP** | S46–S47 | |
| Table S14. Excited states calculated by TDDFT Calculations of **PDI**–**MePY** | S48–S49 | |
| Table S15. Excited states calculated by TDDFT Calculations of [**PDI**–**MeBZ**]**^•−^** | S50–S54 | |
| Table S16. Excited states calculated by TDDFT Calculations of [**PDI**–**MeNP**]**^•−^** | S55–S58 | |
| Table S17. Excited states calculated by TDDFT Calculations of [**PDI**–**MePY**]**^•−^** | S59–S63 | |
| Figure S33. Excited state electron and hole distributions of [**PDI**–**MeR**]**^•−^** | S64 | |
| Figure S34. Current strength susceptibilities and pathways for [**PDI–MeR**]**^•−^** | S65 | |
| Table S18–S20. The induced current strengths of selected bonds in [**PDI–MeR**]**^•−^** | S65–S66 | |
| References | S67 | |

**General Methods**

Based on standard Schlenk techniques, all of the synthesis experimental procedures were performed under a dry argon condition. Reagents and solvents were purchased from commercial sources and used as received without further purification, unless otherwise stated. All steps of synthesis were monitored using thin layer chromatography (TLC) on silica gel-precoated glass plates. Flash column chromatography was performed with silica gel 60 G (230–400 mesh). The synthesized compounds were characterized by ^1^H-NMR or ^13^C{^1^H}-NMR, and elemental analysis. 1H and proton decoupled 13C spectra were measured using a Bruker500 spectrometer operating at 500 MHz and 125 MHz, respectively, and all proton and carbon chemical shifts were measured relative to the internal residual chloroform (99.5% CDCl_3_) or *N,N*-Dimethylformamide (99.5% DMF-d_7_) in the locking solvent. The GC-MS analysis was performed using a highly sensitive Gas Chromatograph/Mass Selective Detector spectrometer (Agilent, 7890B-5977B GC/MSD). The Radical analyses were performed using an electron spin resonance (ESR) spectrometer (Bruker, EMXplus-9.5/2.7). The presence of Co from cobaltocene was confirmed using an inductively coupled plasma–optical emission spectrometer (ICP-OES) (PerkinElmer Avio 500 & Avio 550). The **PDI**–**NO_2_** and **PDIN**–**H** were prepared based on the previously published method. ^[1, 2]^

**Spectroscopic measurements**: The UV-vis absorption spectra were recorded using a Scinco Mega-2100 spectrophotometer in dual beam mode, and the fluorescence emission measurements were carried out using Shimadzu fluorometer (RF-6000) with a wavelength resolution of ~1 nm. Fluorescence lifetimes were measured using a time-resolved photoluminescence spectrometer (Edinburgh Instruments, FLS-1000) equipped with a time-correlated single photon counting (TCSPC) module. A pulsed diode laser was used as the excitation source, and the repetition rate was adjusted to avoid pulse pile-up effects. The excitation wavelength was set to 520 nm for **PDI**–**MeR** and 610 nm for [**PDI**–**MeR**]**^•−^**. The instrument response function (IRF) was measured separately and used for iterative reconvolution fitting of the decay curves. The emission quantum yields (Φ_PL_) were determined using an integrating sphere module equipped in the FLS-1000 system, enabling absolute quantum yield measurements under identical experimental conditions.

**Cyclic Voltammetry (CV)**: A CH Instruments 701D potentiate was used for electrochemical measurements, and cyclic voltammetry (CV) was performed in an electrolytic solution containing the electroactive compounds (1 mM) and tetra-*n*-butylammonium perchlorate (0.1 M TBAP) in deoxygenated DCM and DMF. A three-electrode configuration, glassy carbon, platinum wire, and SCE, were used as working, counter, and reference electrodes, respectively.^[3, 4]^ The scanning rate was 50 mV s^−1^.

**Spectroelectrochemical measurements**: SEC Measurements were carried out using a custom-made, optically transparent, thin-layer electrochemical cell (light pass length = 1 mm) equipped with a platinum mesh working electrode and a platinum coil counter electrode, and a SCE was used as the reference electrode.^[4, 5]^ Three electrodes were placed into the **PDI**–**MeR** solution with 0.1 M TBAP. Potentials were applied using a commercial electrochemical analyzer (CH Instruments 701D potentiostat). The electrochemical analyzer controlled the potential of the working electrode, and a Scinco Nano-MD UV-vis spectrophotometer obtained the absorption spectra of the redox species. SEC absorption spectra was recorded at ~10 sec intervals for 1200 sec to monitor the potential induced spectral evolution.

**Device Fabrication**: Electrochromic and electrofluorochromic devices^[6]^ were fabricated in a sandwich configuration using Fluorine-doped Tin Oxide (FTO)-coated glass substrates (300 × 300 × 2.2 mm, 6 ~ 9 Ω/sq). The device architecture was FTO-coated glass / DMF solution (1 mM **PDI**–**MeR**, 0.1 M TBAP) / FTO-coated glass. All devices were fabricated and measured in air. An external potential of -1.6 V was applied using CH Instruments 701D potentiostat to evaluate the colorimetric and electrofluorochromic performance.

**Calculation Summary**: Geometry optimizations, TD-DFT calculations, electron-hole and spin density analyses, anisotropy of the induced current density (AICD) calculations, and gauge-including magnetically induced current (GIMIC) calculations were performed. More details are provided in the Calculation Details section.**Synthetic procedures and compound data**

*Synthesis of* ***PDI****–****NO_2_*** : A mixture of 2,9-di(pentan-3-yl)anthra[2,1,9-def:6,5,10-d'e'f']diisoquinoline-1,3,8,10(2H,9H)-tetraone (0.5 g, 0.38 mmol), Ammonium Cerium(IV) Nitrate (1 g, 0.76 mmol) HNO_3_ (0.4 mL, 3.5 mmol), H_2_SO_4_ (0.5 mL, 3.94 mmol) in Dichloromethane (50 mL) was refluxed under argon at RT for 15 h. deionized water (50 mL) was poured, and organic layer was separated using a separating funnel. The water layer was washed using DCM (×3) for the extracted remained organic residue. After combined all of the organic solvents, the organic layer was dried over anhydrous MgSO_4_, and then filtered off. The solvent was removed under reduced pressure, and the residue was purified by silica gel column chromatography using DCM/petroleum ether mixture eluent.

*5-nitro-2,9-di(pentan-3-yl)anthra[2,1,9-def:6,5,10-d'e'f']diisoquinoline-1,3,8,10(2H,9H)-tetraone* ***(PDI****–****NO_2_)***. maroon powder (0.374 g, Yield: 69%). ^1^H-NMR (500 MHz, CDCl_3_, ppm): δ= 8.80 (d, J = 8.0 Hz, 1H), 8.74–8.70 (m, 4H), 8.60 (d, J = 8.5 Hz, 1H), 8.26 (d, J = 8.5 Hz, 1H), 5.08–5.02 (m, 2H), 2.29–2.20 (m, 4H), 1.98–1.89 (m, 4H), 0.92 (t, 12H, J = 7.5 Hz). ^13^C{^1^H}-NMR (125 MHz, CDCl_3_, ppm): δ= 147.73, 135.52, 132.97, 129.54, 129.41, 129.16, 127.97, 127.56, 126.72, 126.50, 124.51, 124.05, 58.24, 57.95, 24.99, 24.91, 11.31, 11.28.

*Synthesis of* ***PDIN****–****H*** : A mixture of 5-nitro-2,9-di(pentan-3-yl)anthra[2,1,9-def:6,5,10-d'e'f']diisoquinoline-1,3,8,10(2H,9H)-tetraone (PDI–NO_2_, 0.2 g, 0.35 mmol), Triphenylphosphane (0.23 g, 0.87 mmol) in *o*-Dichlorobenzene (3 mL) was refluxed under argon at 190 ℃ for 3 h. After completing the reaction, remove the solvent. and the residue was purified by silica gel column chromatography using DCM/Acetone mixture eluent.

*2,8-di(pentan-3-yl)-1H-pyrido[3',4',5':4,5]naphtho[2,1,8-cde]pyrido[3',4',5':4,5]naphtho[8,1,2-ghi]isoindole-1,3,7,9(2H,5H,8H)-tetraon* ***(PDIN****–****H)****.* (DCM/Acetone = 20:1). Dark red powder (0.15 g, Yield: 80%). ^1^H-NMR (500 MHz, CDCl_3_, ppm): δ= 9.86 (s, 1H), 9.06 (s, 2H), 9.00 (d, *J* = 8.0 Hz, 2H), 8.88 (d, *J* = 8.0 Hz, 2H), 5.25–5.19 (m, 2H), 2.39–2.32 (m, 4H), 2.04–1.99 (m, 4H), 0.98 (t, *J* = 7.5 Hz, 12H).
^13^C{^1^H}-NMR (125 MHz, CDCl_3_, ppm): δ= 133.68, 133.29, 125.05, 124.17, 122.17, 57.81, 25.25, 11.46.

*Synthesis of* ***PDI****–****MeBZ*** : A mixture of 2,8-di(pentan-3-yl)-1H-pyrido[3',4',5':4,5]naphtho[2,1,8-cde]pyrido[3',4',5':4,5]naphtho[8,1,2-ghi]isoindole-1,3,7,9(2H,5H,8H)-tetraon (PDIN–H, 0.1 g, 0.183 mmol), Benzyl bromide (0.044 mL, 0.368 mmol), K_2_CO_3_ (0.102 g, 0.736 mmol) in Acetone (15 mL) was refluxed under argon at 65 °C for 4 h.
After cooling to RT, deionized water (50 mL) was poured, and organic layer was separated using a separating funnel. The water layer was washed using DCM (×3) for the extracted remained organic residue. After combined all of the organic solvents, the organic layer was dried over anhydrous MgSO_4_, and then filtered off. The solvent was removed under reduced pressure, and the residue was purified by silica gel column chromatography using DCM/*n*-Hexane mixture eluent.

*5-benzyl-2,8-di(pentan-3-yl)-1H-pyrido[3',4',5':4,5]naphtho[2,1,8-cde]pyrido[3',4',5':4,5]naphtho[8,1,2-ghi]isoindole-1,3,7,9(2H,5H,8H)-tetraone* ***(PDI****–****MeBZ)****.* (DCM/*n*-Hexane = 4:1). Red powder (0.06 g, Yield: 53%). ^1^H-NMR (500 MHz, DMF-d_7_, ppm): δ= 9.15 (d, *J* = 8.0 Hz, 2H), 9.06 (s, 2H), 8.73 (d, *J* = 8.0 Hz, 2H), 7.57 (d, *J* = 7.0 Hz, 2H), 7.44−7.41 (m, 2H), 7.37−7.34 (m, 1H), 6.45 (s, 2H), 5.21−5.15 (m, 2H), 2.42−2.33 (m, 4H), 2.08−1.99 (m, 4H), 1.00 (t, *J* = 7.5 Hz, 12H). ^13^C{^1^H}-NMR (125 MHz, CDCl_3_, ppm): δ= 136.17, 135.11, 132.91, 129.35, 128.68, 127.10, 124.89, 124.06, 121.99, 119.93, 57.81, 50.44, 25.24, 11.48. GC-MS (*m/z*) calcd. for C_41_H_35_N_3_O_4_: 633.26; found: 633.4 [M]^+^.

*Synthesis of* ***PDI****–****MeNP*** : A mixture of *2,8-di(pentan-3-yl)-1H-pyrido[3',4',5':4,5]naphtho[2,1,8-cde]pyrido[3',4',5':4,5]naphtho[8,1,2-ghi]isoindole-1,3,7,9(2H,5H,8H)-tetraon* (PDIN–H, 0.1g, 0.183 mmol), 2-(Bromomethyl)naphthalene (0.081 g, 0.368 mmol),
K_2_CO_3_ (0.102 g, 0.736 mmol) in Acetone (15 mL) was refluxed under argon at 65 °C for 4 h. After cooling to RT, deionized water (50 mL) was poured, and organic layer was separated using a separating funnel. The water layer was washed using DCM (×3) for the extracted remained organic residue. After combined all of the organic solvents, the organic layer was dried over anhydrous MgSO_4_, and then filtered off. The solvent was removed under reduced pressure, and the residue was purified by silica gel column chromatography using DCM/*n*-Hexane mixture eluent.

*5-(naphthalen-2-ylmethyl)-2,8-di(pentan-3-yl)-1H-pyrido[3',4',5':4,5]naphtho[2,1,8-cde]pyrido[3',4',5':4,5]naphtho[8,1,2-ghi]isoindole-1,3,7,9(2H,5H,8H)-tetraone*. ***(PDI****–****MeNP)*** (DCM/*n*-Hexane = 4:1). Red powder (0.064 g, Yield: 51%). ^1^H-NMR (500 MHz, DMF-d_7_, ppm): δ= 9.22 (d, *J* = 8.0 Hz, 2H), 9.14 (s, 2H), 8.77 (d, *J* = 8.0 Hz, 2H), 8.13 (s, 1H), 7.96 (d, *J* = 8.5 Hz, 1H), 7.93−7.88 (m, 2H), 7.65 (dd, *J* = 8.5, 1.5 Hz, 1H), 7.53−7.51 (m, 2H), 6.66 (s, 2H), 5.20−5.14 (m, 2H), 2.39−2.33 (m, 4H) 2.06−1.98 (m, 4H), 0.98 (t, *J* = 7.5 Hz, 12H). ^13^C{^1^H}-NMR (125 MHz, CDCl_3_, ppm): δ= 135.19, 133.67, 133.31, 133.14, 132.95, 129.40, 127.96, 127.80, 126.77, 126.59, 126.00, 124.93, 124.46, 124.09, 122.04, 120.00, 57.81, 50.60, 31.60, 25.23, 22.66, 14.12, 11.47. GC-MS (*m/z*) calcd. for C_45_H_37_N_3_O_4_: 683.28; found: 683.4 [M]^+^.

*Synthesis of* ***PDI****–****MePY***: A mixture of *2,8-di(pentan-3-yl)-1H-pyrido[3',4',5':4,5]naphtho[2,1,8-cde]pyrido[3',4',5':4,5]naphtho[8,1,2-ghi]isoindole-1,3,7,9(2H,5H,8H)-tetraon* (PDIN–H, 0.1g, 0.183 mmol), 1-(Bromomethyl)pyrene (0.081 g, 0.276 mmol), K_2_CO_3_ (0.076g, 0.552 mmol) in Acetone (15 mL) was refluxed under argon at 65°C for 4 h. After cooling to RT, deionized water (50 mL) was poured, and organic layer was separated using a separating funnel. The water layer was washed using DCM (×3) for the extracted remained organic residue. After combined all of the organic solvents, the organic layer was dried over anhydrous MgSO_4_, and then filtered off. The solvent was removed under reduced pressure, and the residue was purified by silica gel column chromatography using DCM/*n*-Hexane mixture eluent.

*2,8-di(pentan-3-yl)-5-(pyren-1-ylmethyl)-1H-pyrido[3',4',5':4,5]naphtho[2,1,8-cde]pyrido[3',4',5':4,5]naphtho[8,1,2-ghi]isoindole-1,3,7,9(2H,5H,8H)-tetraone* ***(PDI****–****MePY)*** (DCM/*n*-Hexane = 4:1). Orange powder (0.074 g, Yield: 53%). ^1^H-NMR (500 MHz, DMF-d_7_, ppm): δ= 9.26 (d, *J* = 8.0 Hz, 2H), 9.06 (s, 2H), 8.80–8.75 (m, 3H), 8.36–8.33 (m, 3H), 8.28 (d, *J* = 8.0 Hz, 1H), 8.23–8.18 (m, 2H), 8.12 (t, *J* = 7.5 Hz, 1H), 7.94 (d, *J* = 7.5 Hz, 1H), 7.28 (s, 2H), 5.14 (s, 2H), 2.33–2.30 (m, 4H), 2.01–1.95 (m, 4H), 0.94 (t, *J* = 7.5Hz, 12H). ^13^C{^1^H}-NMR (125 MHz, CDCl_3_, ppm): δ= 135.42, 132.92, 131.78, 131.18, 130.46, 129.04, 128.57, 128.40, 127.96, 127.31, 126.27, 125.88, 125.68 125.06, 124.89, 124.50, 124.05, 122.03, 121.27, 120.05, 57.76, 48.85, 25.19, 11.46. GC-MS (*m/z*) calcd. for C_51_H_39_N_3_O_4_: 757.31; found: 757.4 [M]^+^.


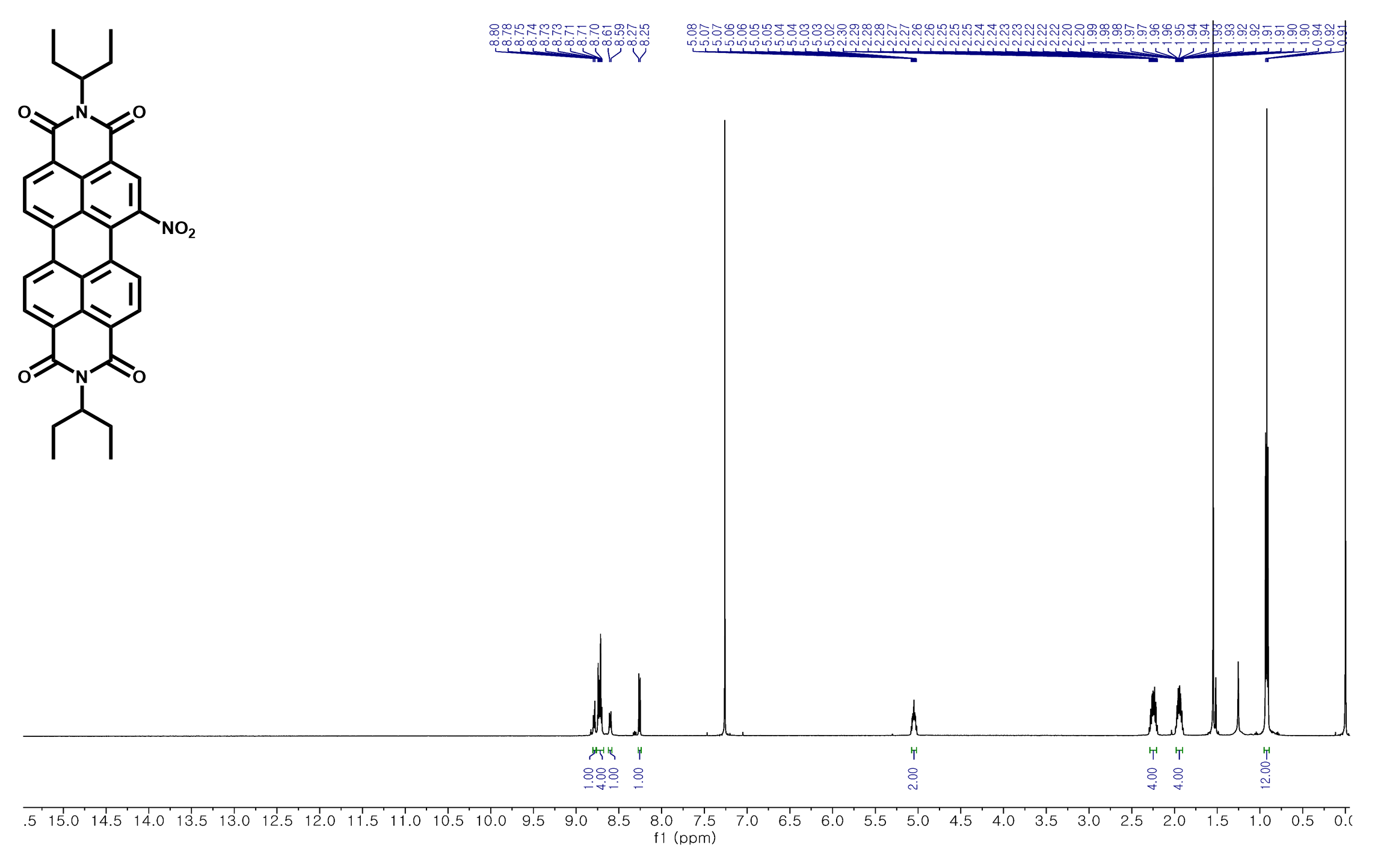


Figure S1. ^1^H-NMR spectrum of **PDI**–**NO_2_** in CDCl_3_ (500 MHz, 293 K).


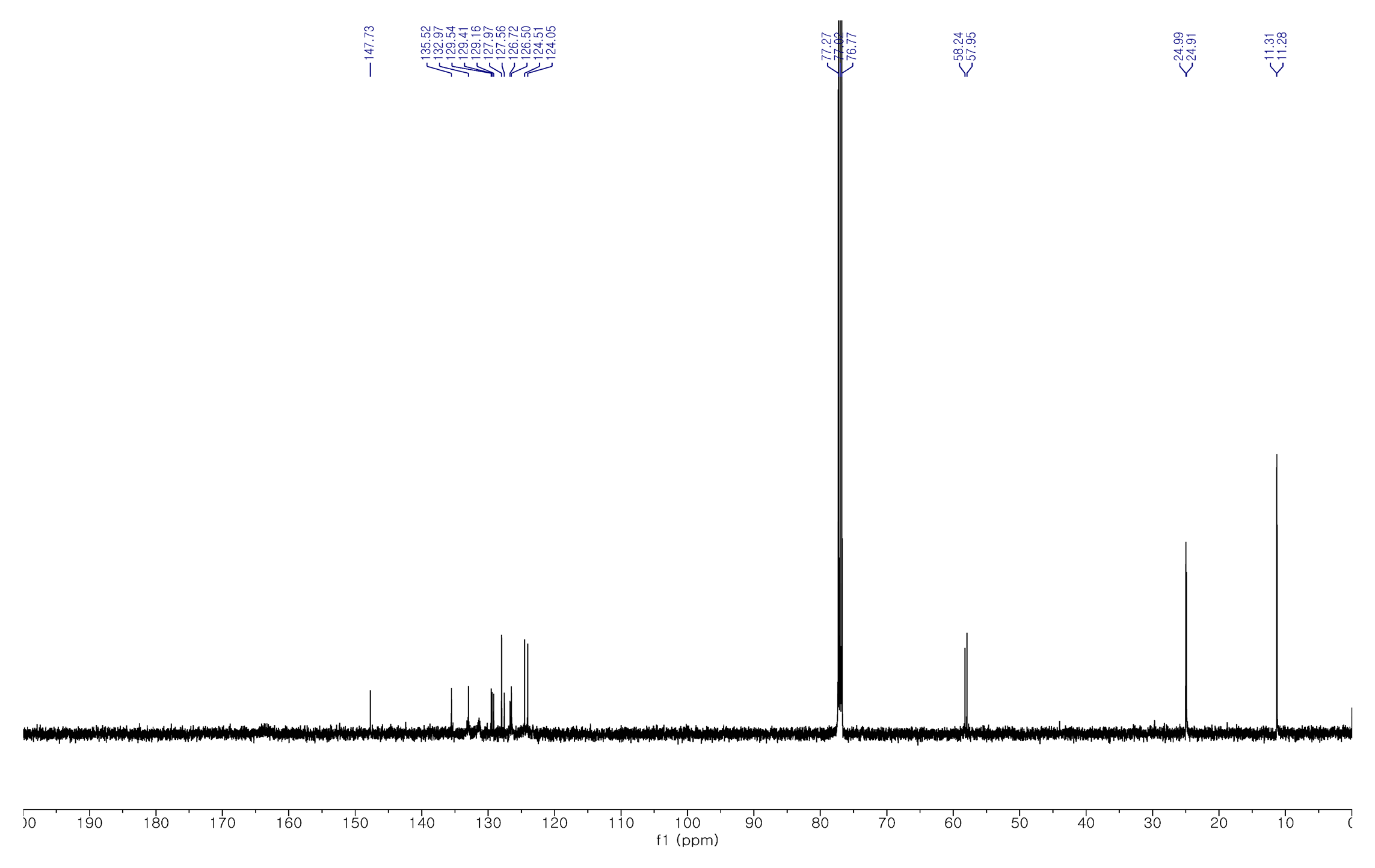


Figure S2. ^13^C{^1^H}-NMR spectrum of **PDI**–**NO_2_** in CDCl_3_ (125 MHz, 293 K).


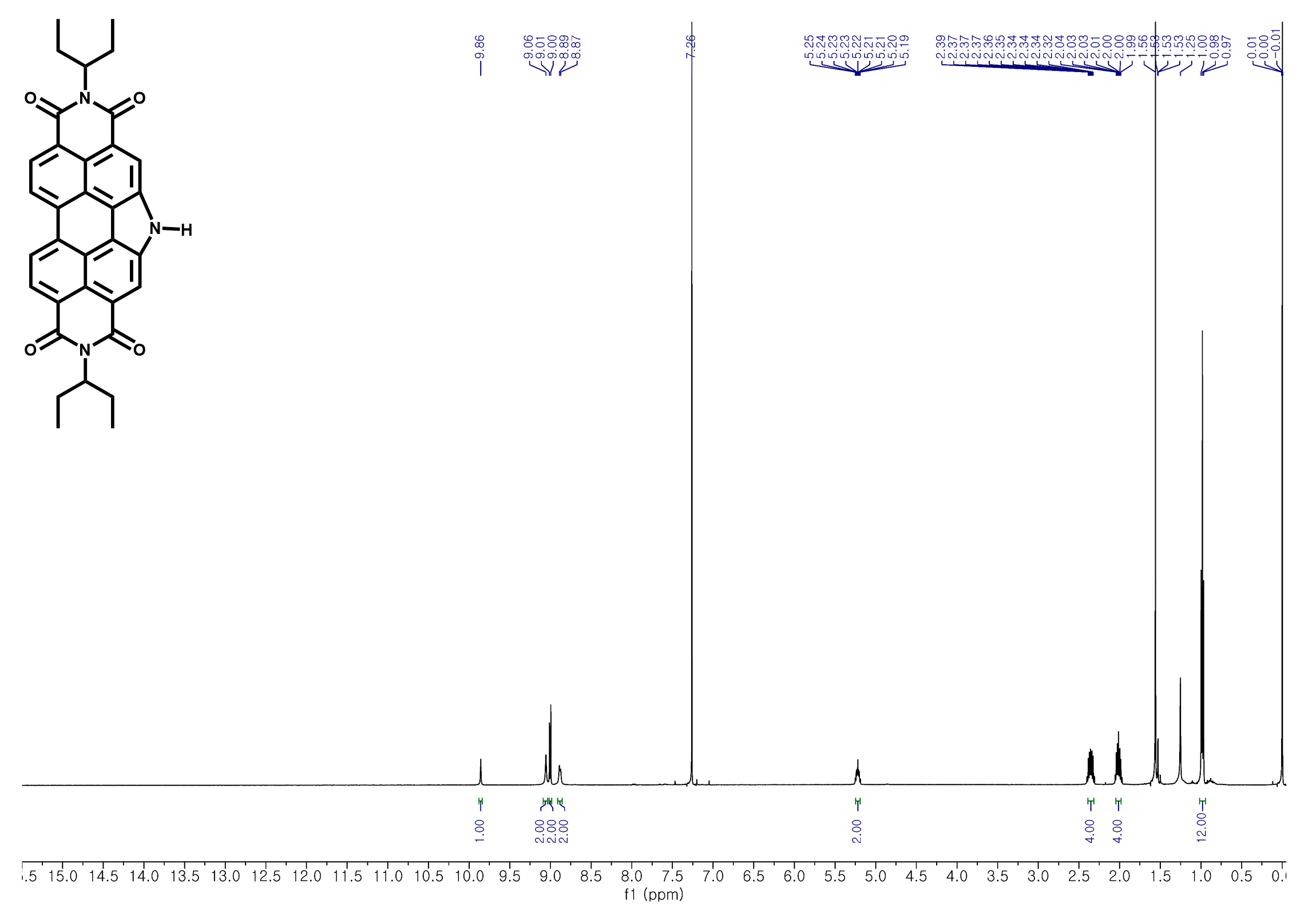


Figure S3. ^1^H-NMR spectrum of **PDIN**–**H** in CDCl_3_ (500 MHz, 293 K).


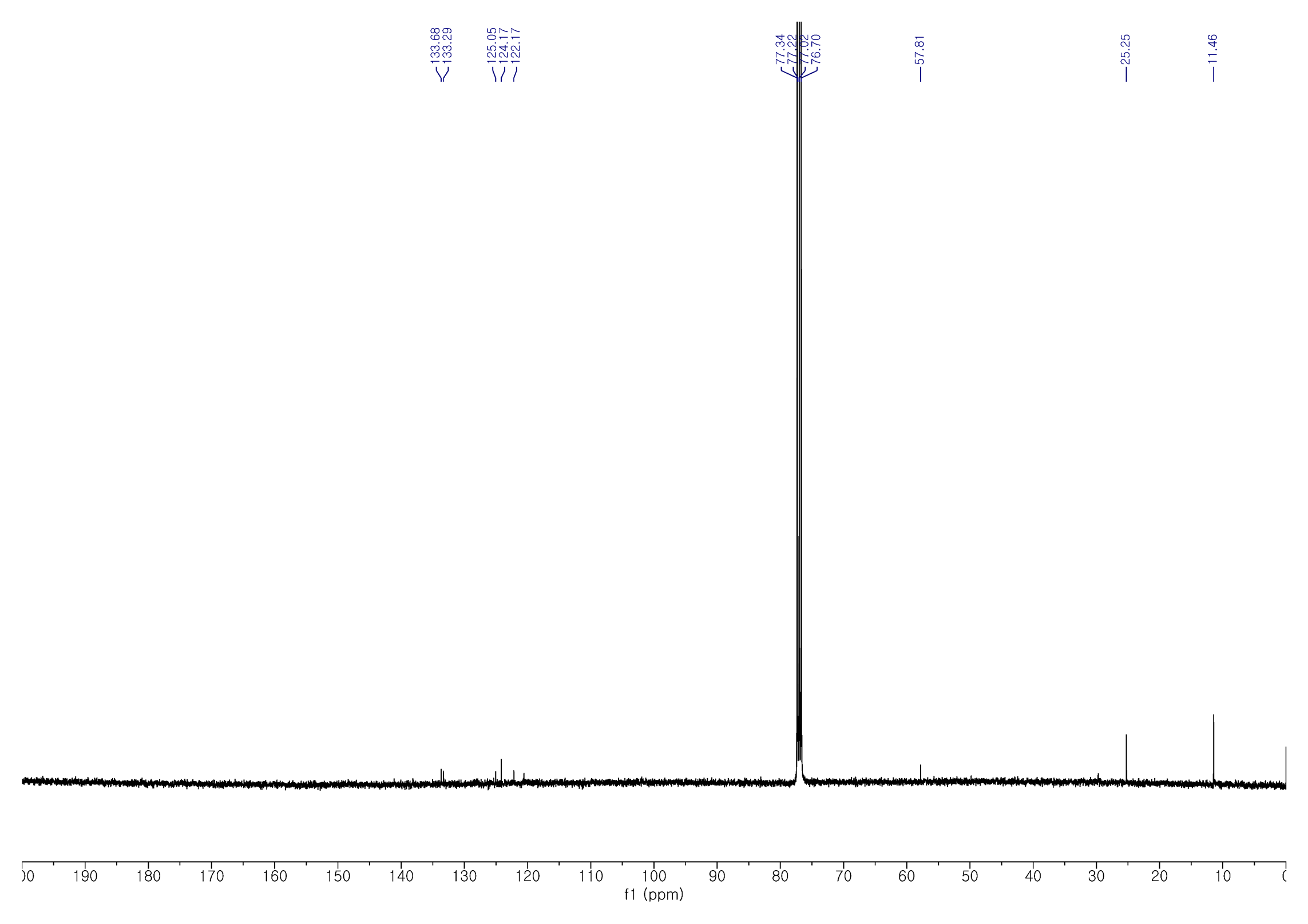


Figure S4. ^13^C{^1^H}-NMR spectrum of **PDIN**–**H** in CDCl_3_ (125 MHz, 293 K).


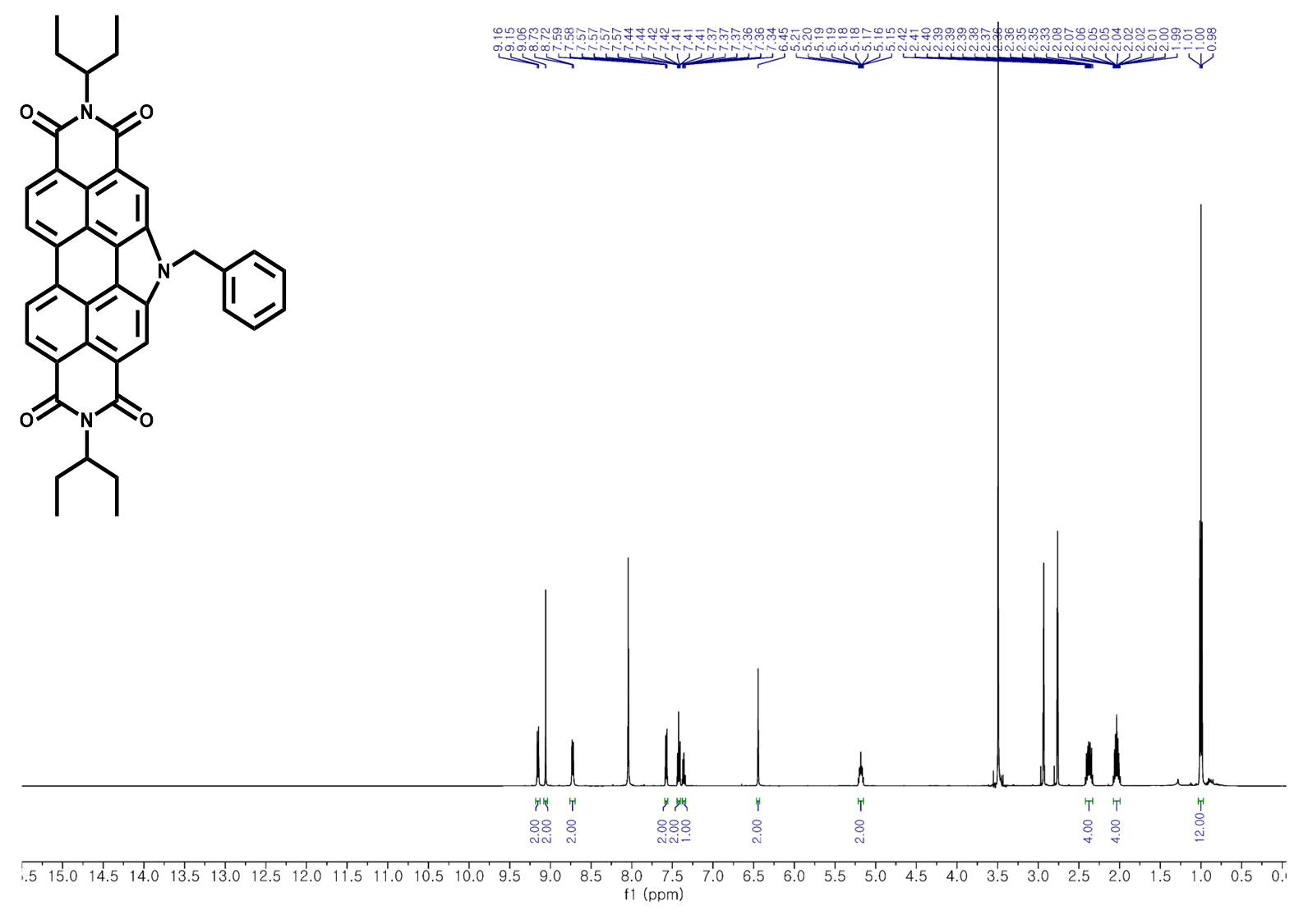


Figure S5. ^1^H-NMR spectrum of **PDI**–**MeBZ** in DMF-d_7_ (500 MHz, 293 K).


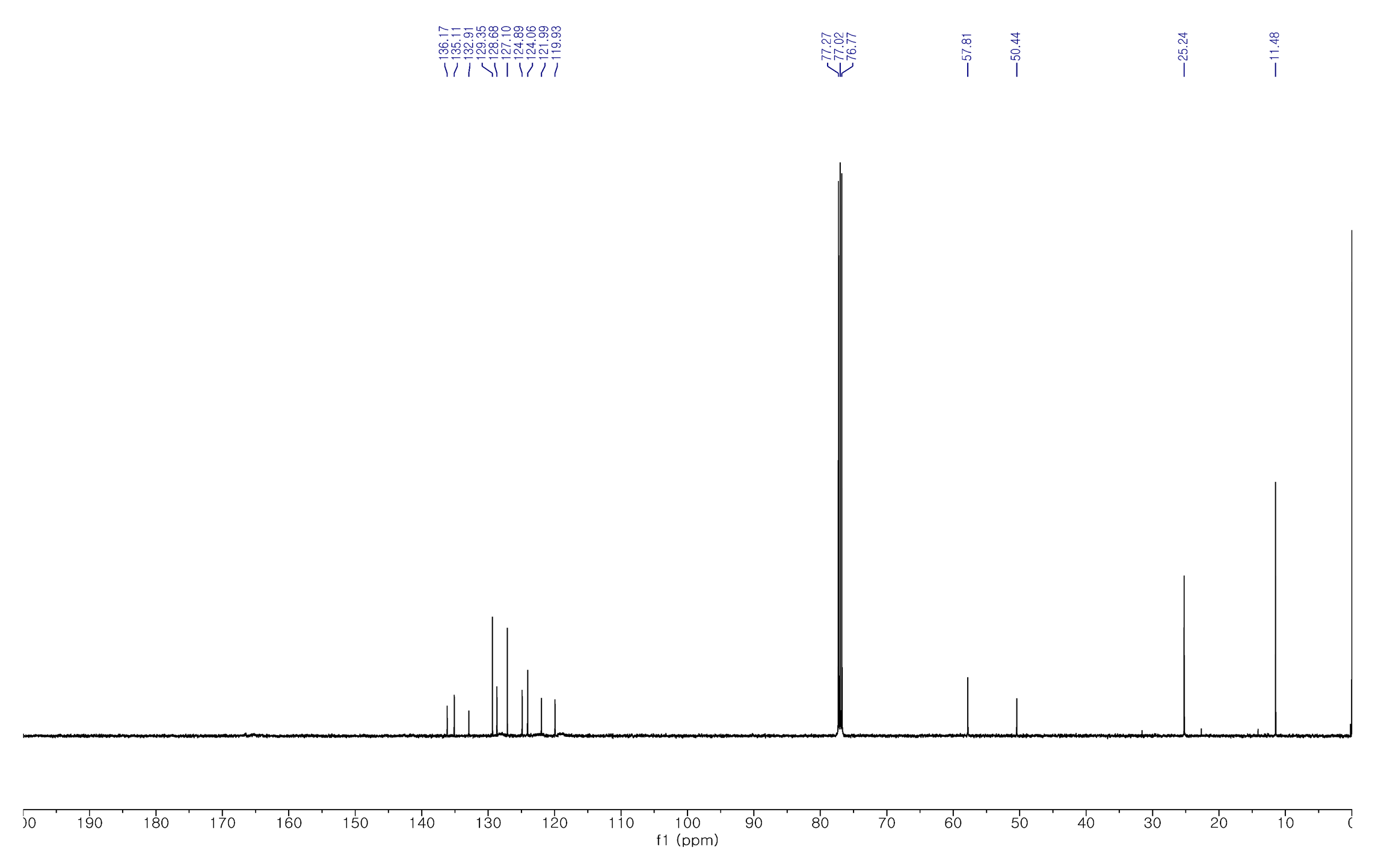


Figure S6. ^13^C{^1^H}-NMR spectrum of **PDI**–**MeBZ** in CDCl_3_ (125 MHz, 293 K).


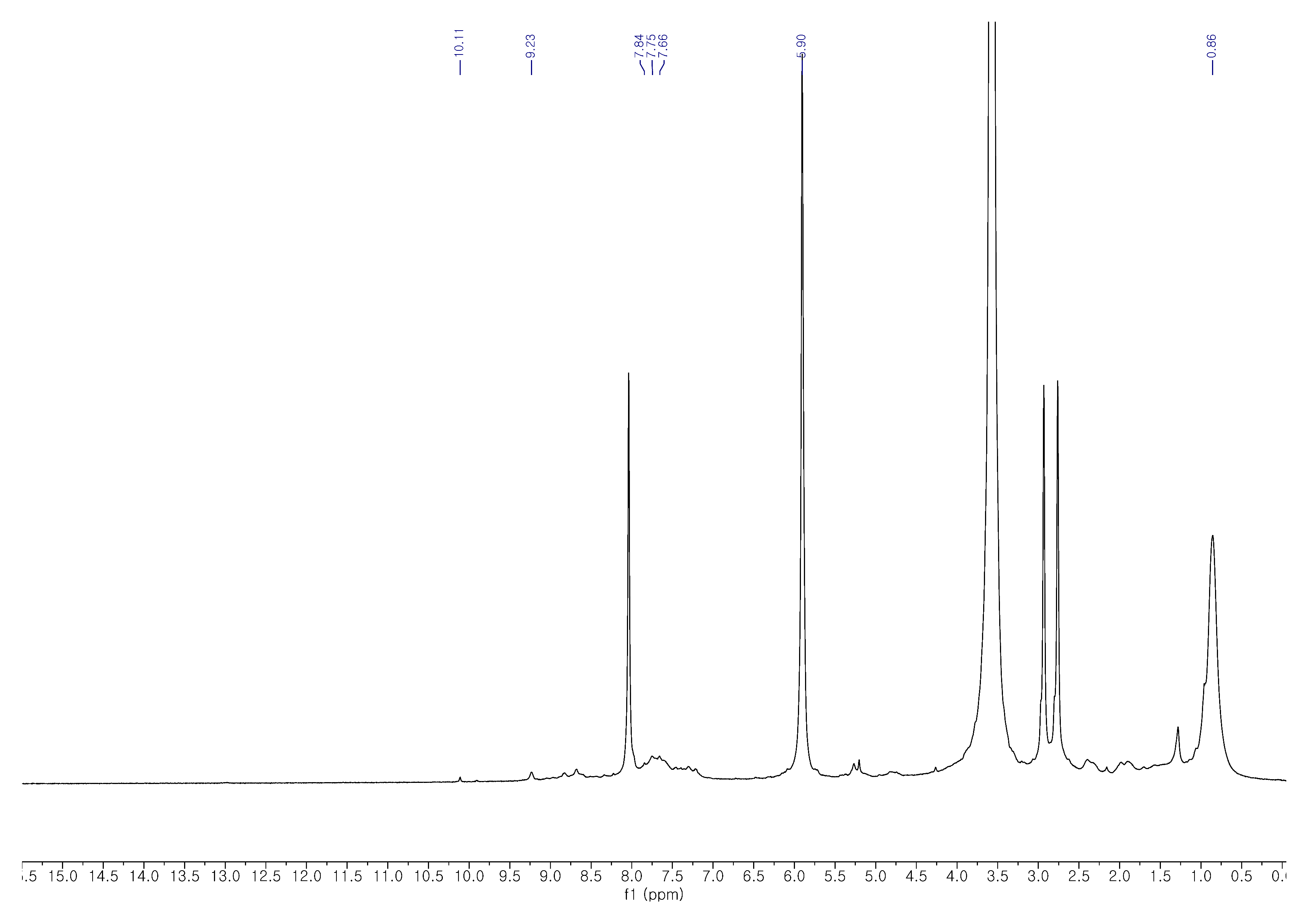
 Figure S7. ^1^H-NMR spectrum of [**PDI**–**MeBZ**]**^•−^** in DMF-d_7_ (500 MHz, 293 K).


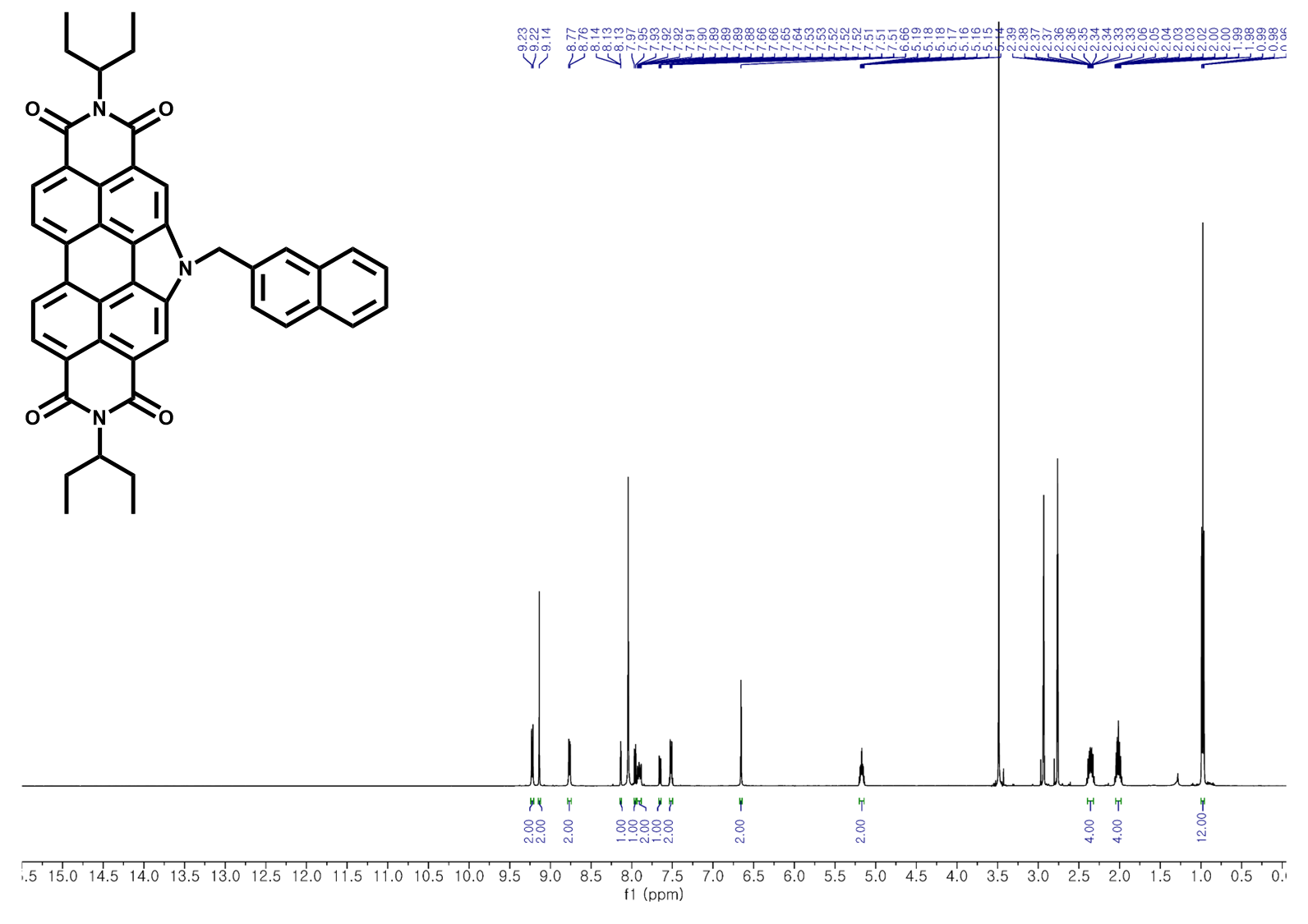


Figure S8. ^1^H-NMR spectrum of **PDI**–**MeNP** in DMF-d_7_ (500 MHz, 293 K).


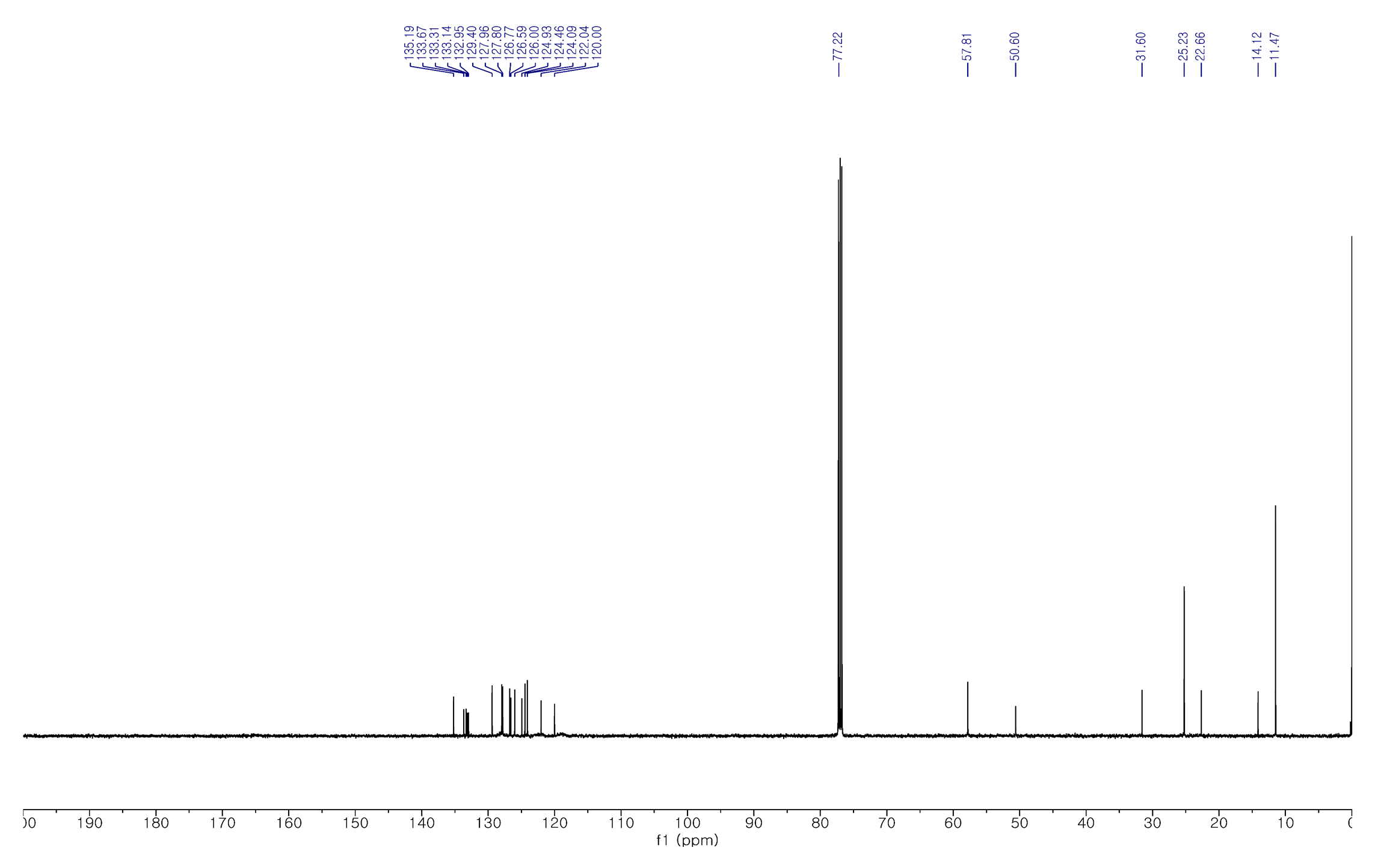


Figure S9. ^13^C{^1^H}-NMR spectrum of **PDI**–**MeNP** in CDCl_3_ (125 MHz, 293 K).


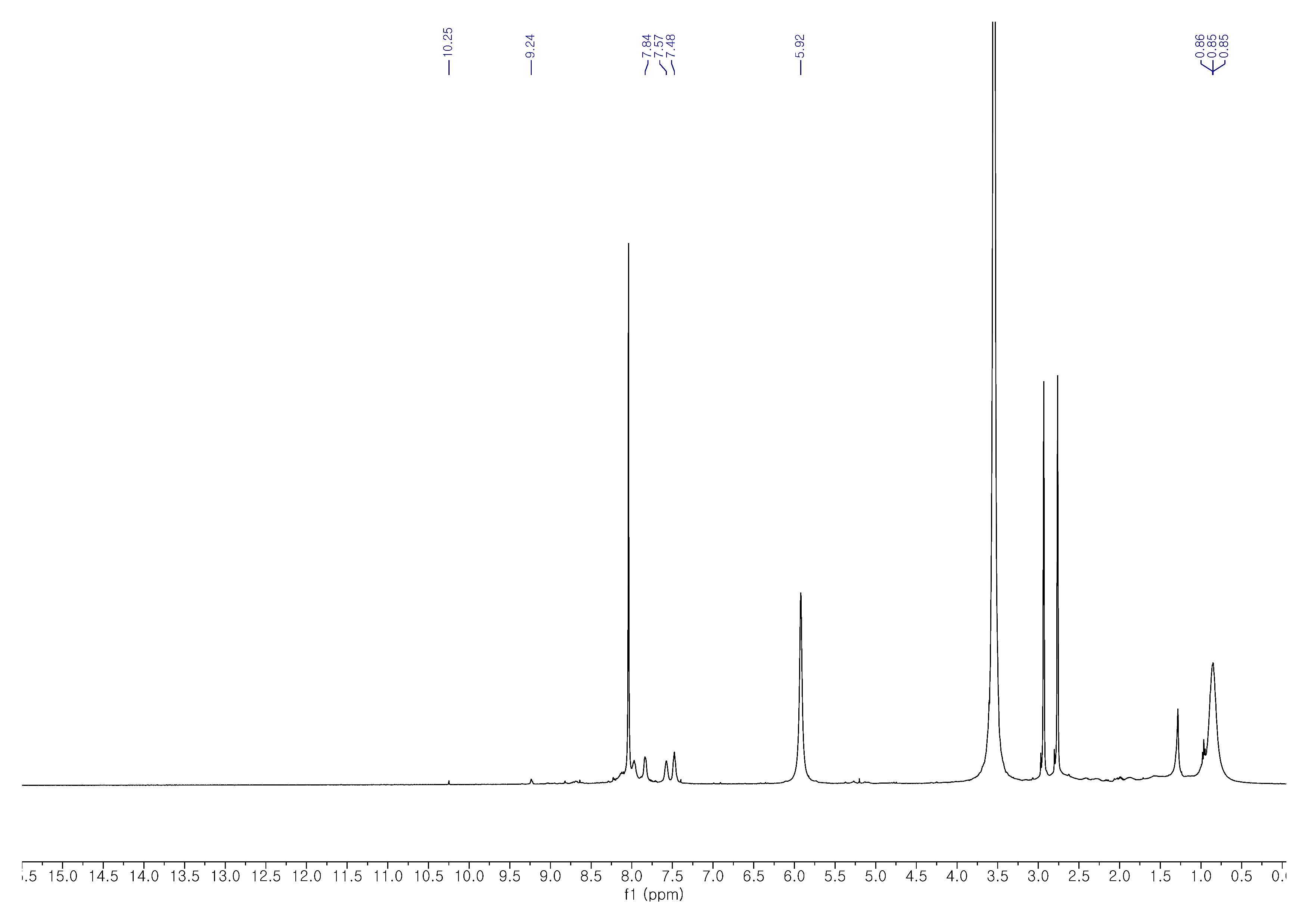
 Figure S10. ^1^H-NMR spectrum of [**PDI**–**MeNP**]**^•−^** in DMF-d_7_ (500 MHz, 293 K).


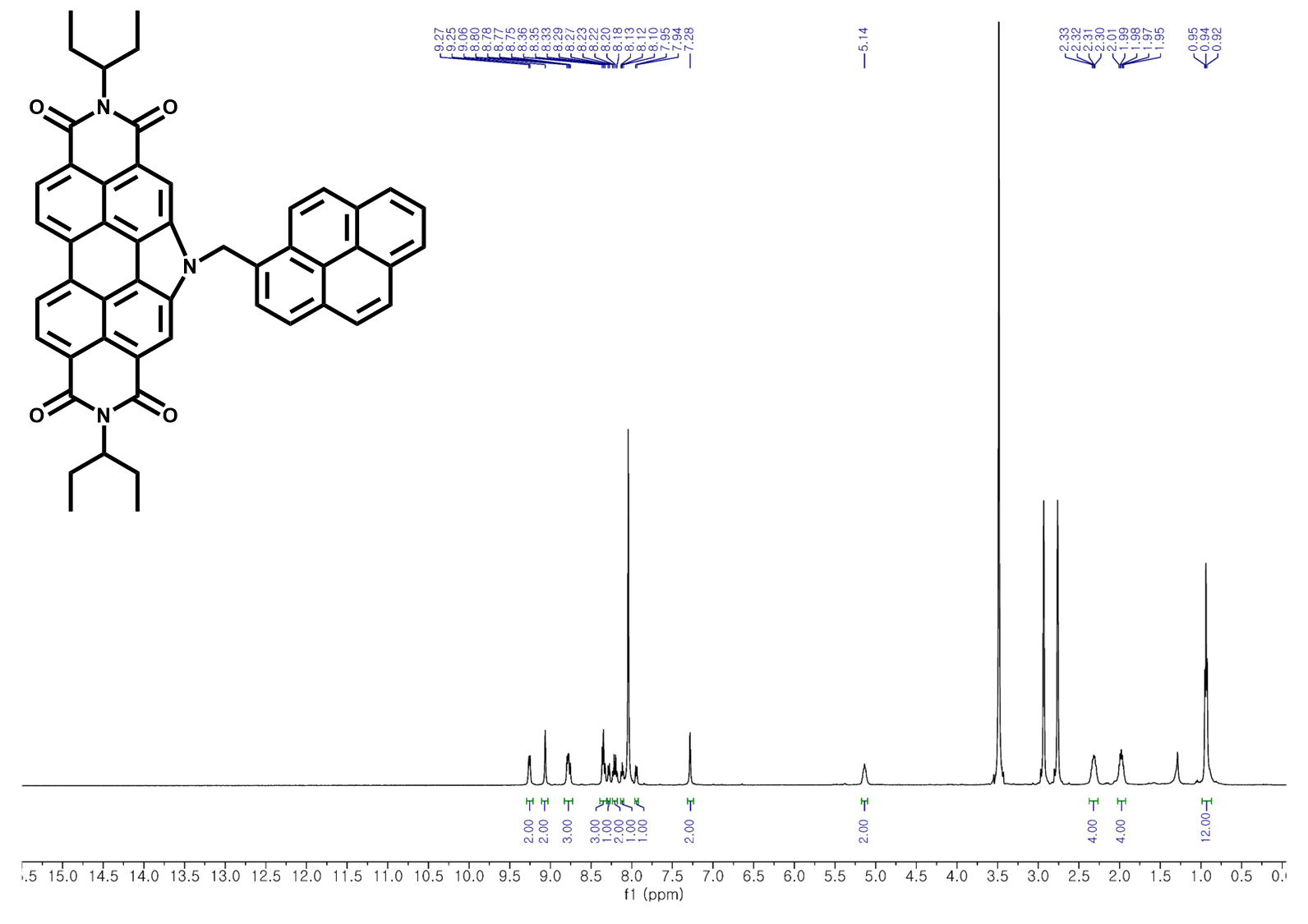


Figure S11. ^1^H-NMR spectrum of **PDI**–**MePY** in DMF-d_7_ (500 MHz, 293 K).


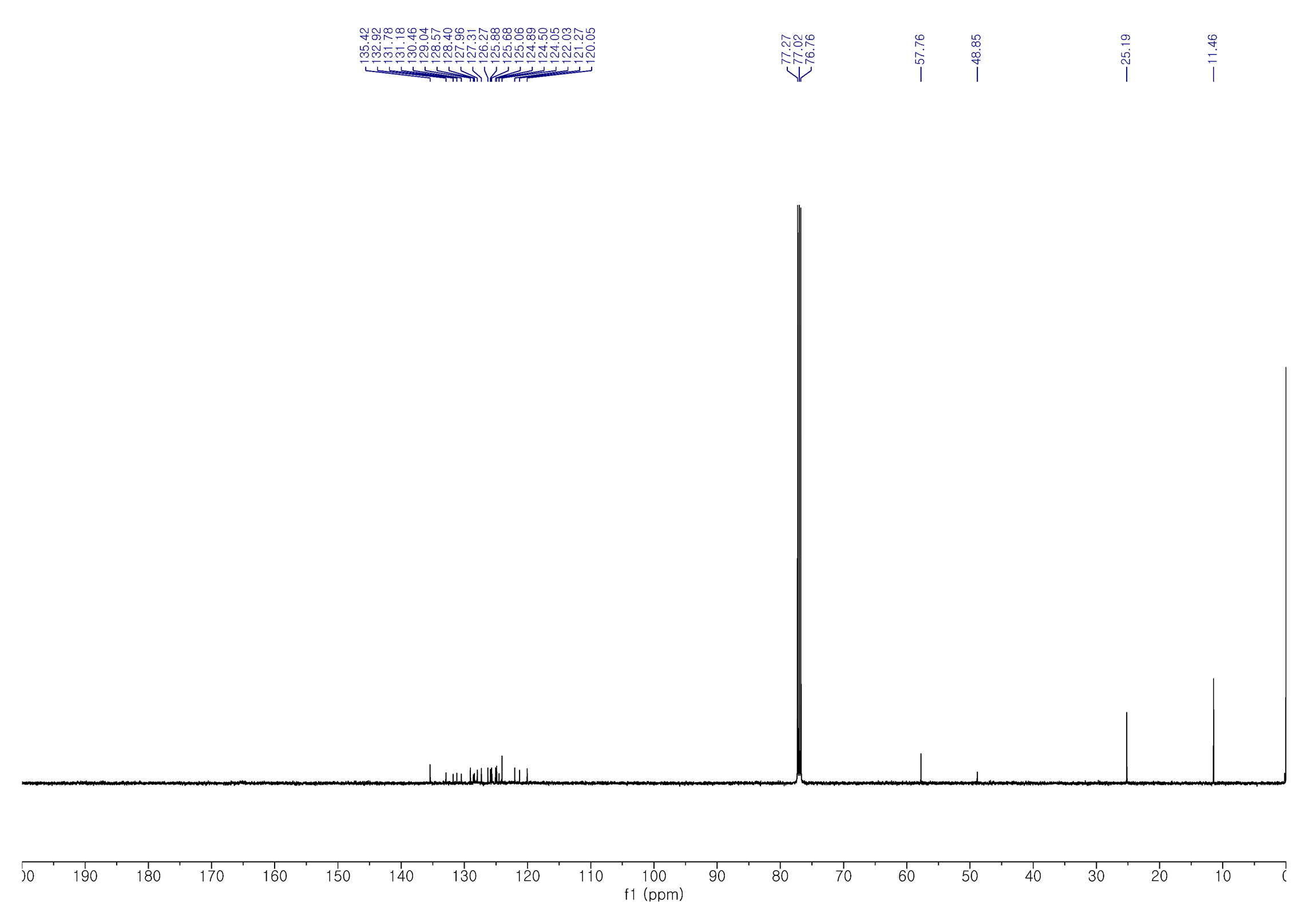


Figure S12. ^13^C{^1^H}-NMR spectrum of **PDI**–**MePY** in CDCl_3_ (125 MHz, 293 K).


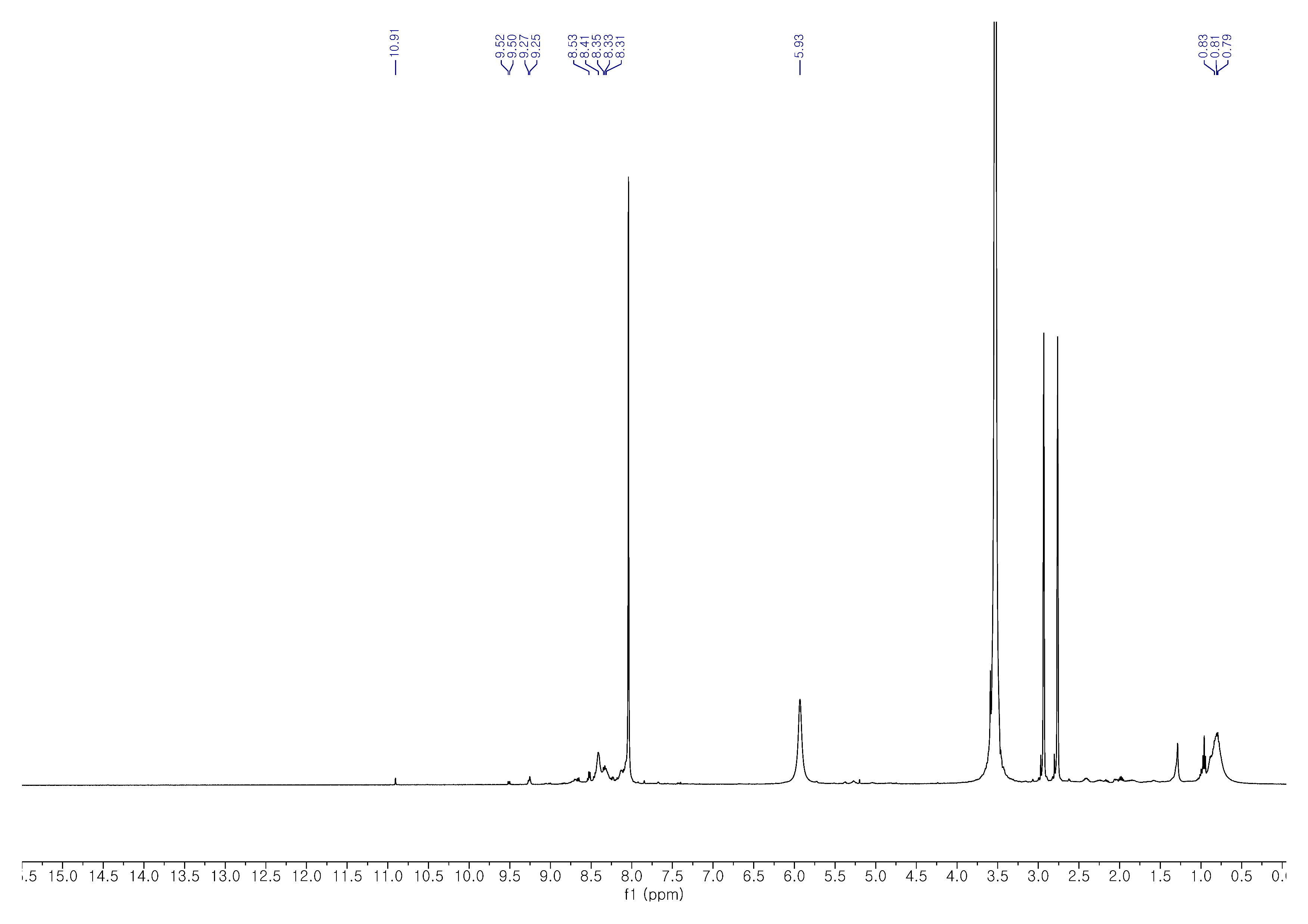
 Figure S13. ^1^H-NMR spectrum of [**PDI**–**MePY**]**^•−^** in DMF-d_7_ (500 MHz, 293 K).


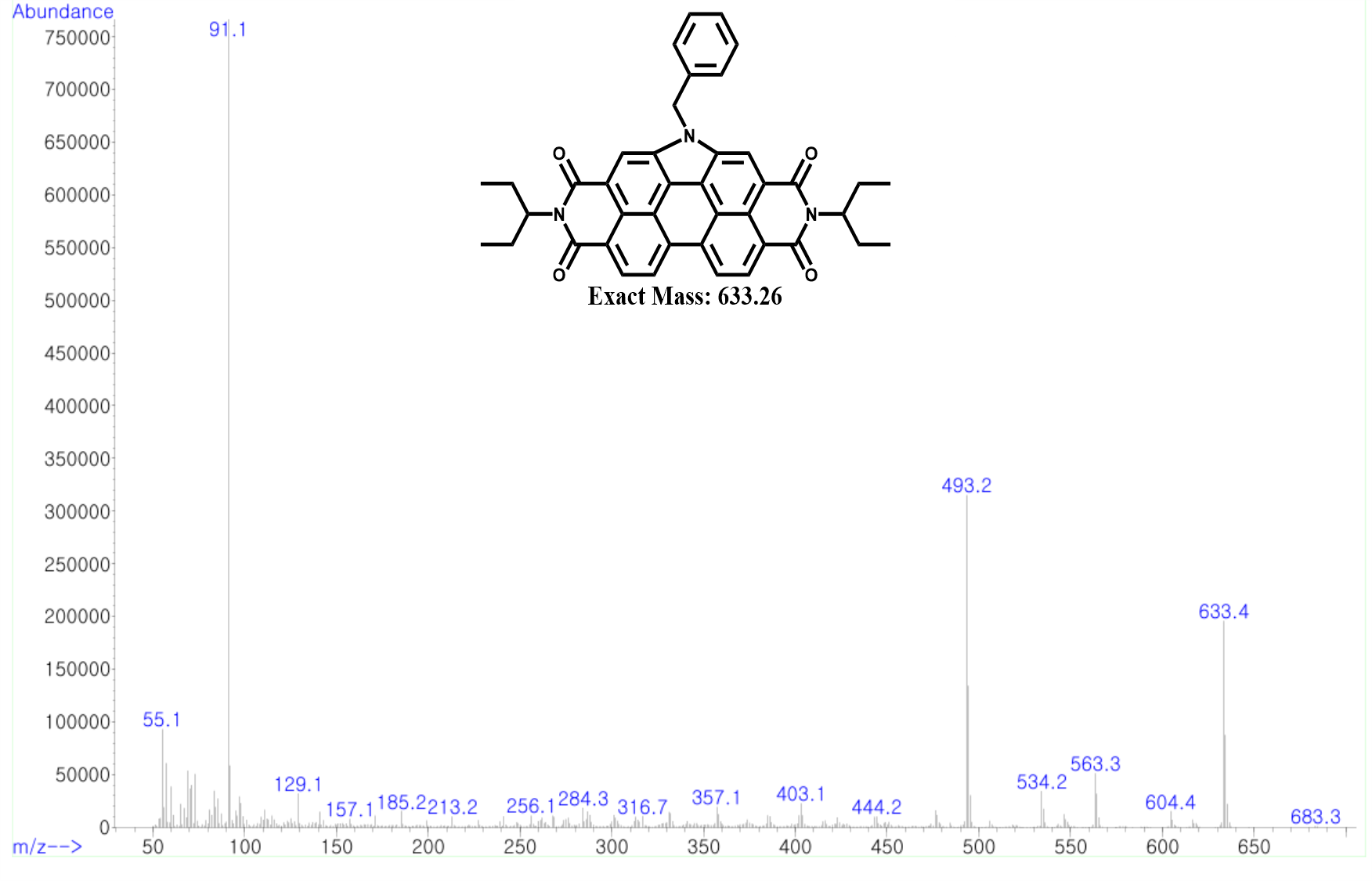


**(A)**


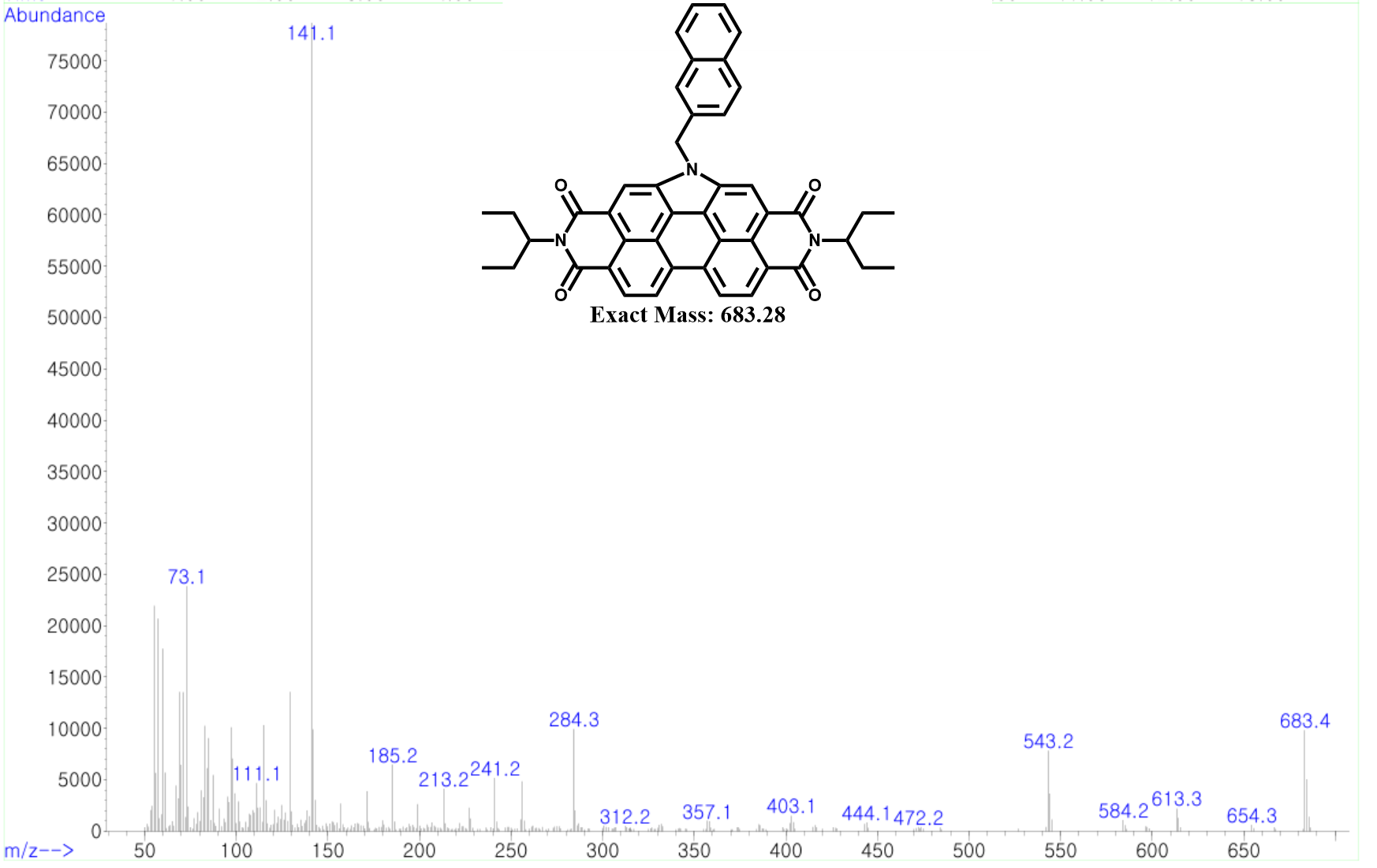


**(B)**


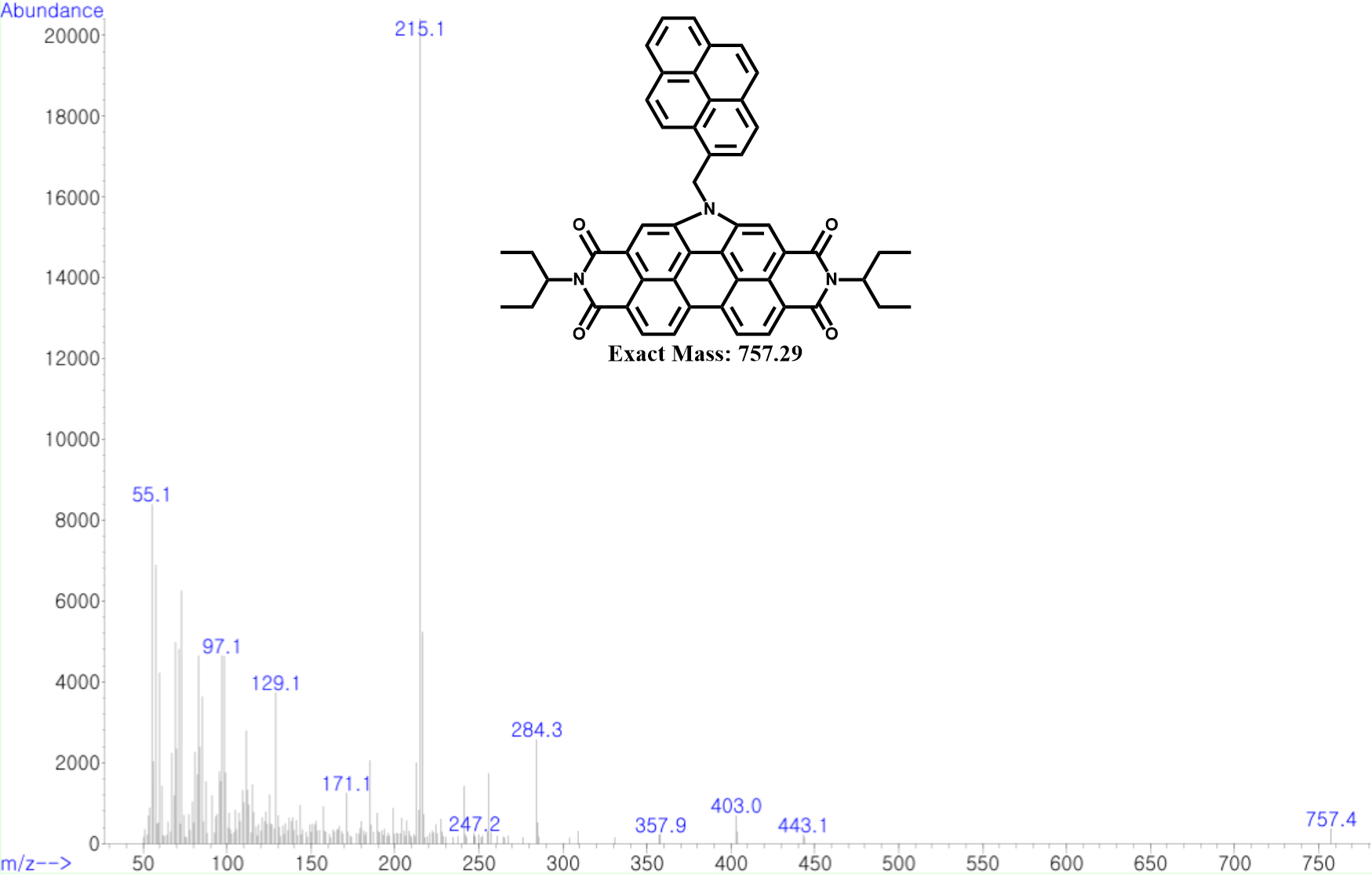


**(C)**

Figure S14. GC-MS data of (A) **PDI**–**MeBZ**, (B) **PDI**–**MeNP**, and (C) **PDI**–**MePY**.









**(B)**

**(A)**

**(C)**

Figure S15. ICP–OES spectra of (A) [**PDI**–**MeBZ**]**^•−^**, (B) [**PDI**–**MeNP**]**^•−^**, and (C) [**PDI**–**MePY**]**^•−^**, showing the characteristic emission line of Co^3+^ at 228.616 nm.


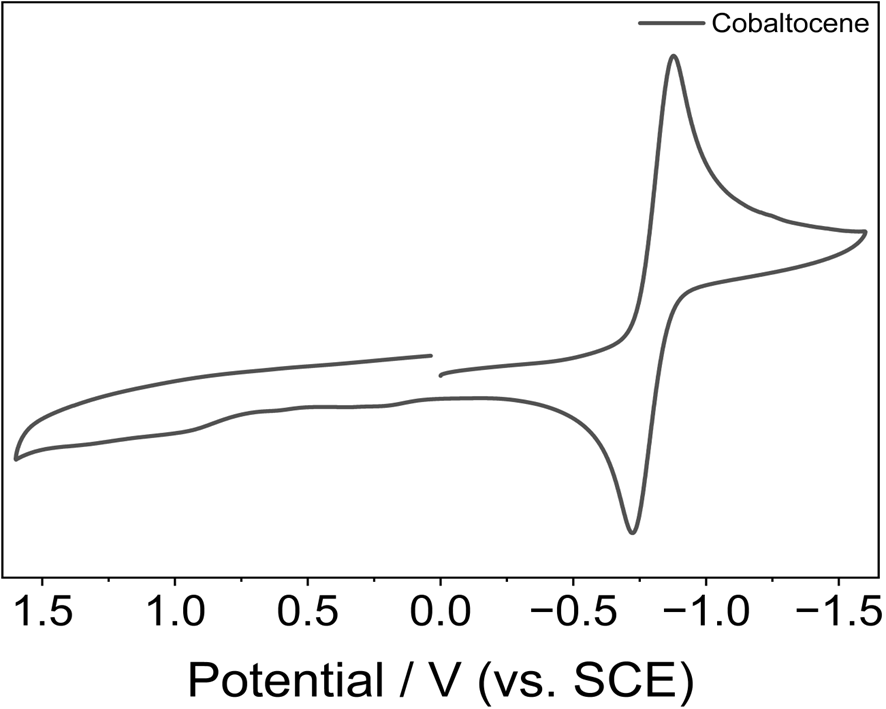


Figure S16. Cyclic voltammogram for the oxidation and reduction of **Cobaltocene** (1 mM) containing 0.1 M TBAP as a supporting electrolyte at room temperature under an argon atmosphere (scan rate= 50 mV s^−1^).

Table S1. Photophysical parameters of the synthesized **PDI**–**MeR** and their chemically generated radical anions [**PDI**–**MeR**]^•−^

| Compound | λ_abs_ /  nm ^a,b)^ | ε /  10^3^ M^–1^ cm^–1 a,b)^ | λ_em_ /  nm ^a,c)^ | Stokes shift /  nm ^a,b,c)^ | *Φ*_PL_ /  % ^a,d)^ | *τ*_PL_/  ns ^a,e)^ | k_r_ /  10^7^ s^–1 a,f)^ | k_nr_ /  10^7^ s^–1 a,g)^ |
| --- | --- | --- | --- | --- | --- | --- | --- | --- |
| **PDI**–**MeBZ** | 525 | 38.08 | 544 | 19 | 65.04 | 3.85 | 16.89 | 9.08 |
| **PDI**–**MeNP** | 526 | 37.46 | 544 | 20 | 61.00 | 3.64 | 16.76 | 10.71 |
| **PDI**–**MePY** | 526 | 34.19 | - | - | 0 | 3.34 | 0 | 29.94 |
| [**PDI**–**MeBZ**]^•−^ | 687,780,  839,928 | 37.71,10.62,  7.96,13.08 | 624 | - | 19.99 | 4.46 | 4.482 | 17.94 |
| [**PDI**–**MeNP**]^•−^ | 688,780,  839,928 | 42.78,12.21,  9.28,15.58 | 624 | - | 23.00 | 4.64 | 4.96 | 16.60 |
| [**PDI**–**MePY**]^•−^ | 688,780,  839,928 | 37.41,10.03,  7.65,13.50 | 625 | - | 29.33 | 6.33 | 4.63 | 11.16 |

^a)^Sample in 0.1mM *N,N*-dimethylformamide solution.

^b)^Peaks of UV-vis absorption.

^c)^Photoluminescence peak wavelength, λ_ex_ = 520 nm for PDI–MeR and 610 nm for [PDI–MeR]^•−^

^d)^Photoluminescence quantum efficiency, λ_ex_ = 520 nm for PDI–MeR and 609 nm for [PDI–MeR]^•−^

^e)^Photoluminescence lifetime, λ_ex_ = 525 nm for PDI–MeR and 610 nm for [PDI–MeR]^•−^

^f)^Radiative decay rate.

^g)^Nonradiative decay rate.

Table S2. Cyclic voltammetry data for the observed reduction waves of **PDI**–**MeR** measured in DCM with 0.1 M TBAP *vs. SCE*

|  | 1e^–^ waves (0/–1) | | | | 1e^–^ waves (–1/–2) | | | |
| --- | --- | --- | --- | --- | --- | --- | --- | --- |
| Compound | E_onset_ ^red1^ /  eV ^a)^ | E_pc_ ^red1^ /  eV ^b)^ | E_pa_ ^red1^ /  eV ^b)^ | E_1/2_ ^red1^ /  eV ^b)^ | E_onset_ ^red2^ /  eV ^a)^ | E_pc_ ^red2^ /  eV ^b)^ | E_pa_ ^red2^ /  eV ^b)^ | E_1/2_ ^red2^ /  eV ^b)^ |
| **PDI**–**MeBZ** | -0.6611 | -0.8317 | -0.6830 | -0.7574 | -0.9439 | -1.0788 | -0.9375 | -1.0082 |
| **PDI**–**MeNP** | -0.6365 | -0.8408 | -0.6502 | -0.7455 | -0.9530 | -1.0962 | -0.9129 | -1.0046 |
| **PDI**–**MePY** | -0.6247 | -0.8207 | -0.6420 | -0.7314 | -0.9366 | -1.0725 | -0.9028 | -0.9877 |

^a)^Onset potentials of the first and second reversible reduction waves.
^b)^E_1/2_ = (E_pa_ + E_pc_) / 2, in which E_pa_ (E_pc_) are anodic (cathodic) peak potentials in cyclic voltammetry.

Table S3. Cyclic voltammetry data for the observed reduction waves of **PDI**–**MeR** and [**PDI**–**MeR**]^•−^ measured in DMF with 0.1 M TBAP *vs. SCE*

|  | 1e^–^ waves (0/–1) | | | | 1e^–^ waves (–1/–2) | | | |
| --- | --- | --- | --- | --- | --- | --- | --- | --- |
| Compound | E_onset_ ^red1^ /  eV ^a)^ | E_pc_ ^red1^ /  eV ^b)^ | E_pa_ ^red1^ /  eV ^b)^ | E_1/2_ ^red1^ /  eV ^b)^ | E_onset_ ^red2^ /  eV ^a)^ | E_pc_ ^red2^ /  eV ^b)^ | E_pa_ ^red2^ /  eV ^b)^ | E_1/2_ ^red2^ /  eV ^b)^ |
| **PDI**–**MeBZ** | -0.8830 | -0.9569 | -0.8344 | -0.8956 | - | - | - | - |
| **PDI**–**MeNP** | -0.8755 | -0.9675 | -0.7946 | -0.8811 | - | - | - | - |
| **PDI**–**MePY** | -0.8777 | -0.9608 | -0.7985 | -0.8830 | - | - | - | - |
| [**PDI**–**MeBZ**]^•−^ | -0.6766 | -0.6942 | -0.6401 | -0.6672 | -0.9072 | -0.9723 | -0.8445 | -0.9084 |
| [**PDI**–**MeNP**]^•−^ | -0.6611 | -0.6858 | -0.6268 | -0.6563 | -0.8828 | -0.9361 | -0.8344 | -0.8853 |
| [**PDI**–**MePY**]^•−^ | -0.7979 | -0.6880 | -0.9176 | -0.8028 | -1.1604 | -0.9530 | -1.3564 | -1.1547 |

^a)^Onset potentials of the first and second reversible reduction waves.
^b)^E_1/2_ = (E_pa_ + E_pc_) / 2, in which E_pa_ (E_pc_) are anodic (cathodic) peak potentials in cyclic voltammetry.





**(A)**





**(B)**





**(C)**

Figure S17. UV-vis absorption and fluorescence spectra in DMF solution of (A) **PDI**–**MeBZ**, (B) **PDI**–**MeNP**, and (C) **PDI**–**MePY**.







**(B)**

**(A)**







**(C)**







Figure S18. UV–vis absorption and fluorescence spectra in different solvents of (A) **PDI**–**MeBZ**, (B) **PDI**–**MeNP**, and (C) **PDI**–**MePY**.







**(B)**

**(A)**

Figure S19. Cyclic voltammogram for the oxidation and reduction of **PDI**–**MeBZ**, **PDI**–**MeNP**, and **PDI**–**MePY** in (A) DCM and (B) DMF solution (1 mM) containing 0.1 M TBAP as a supporting electrolyte at room temperature under an argon atmosphere (scan rate= 50 mV s^−1^).


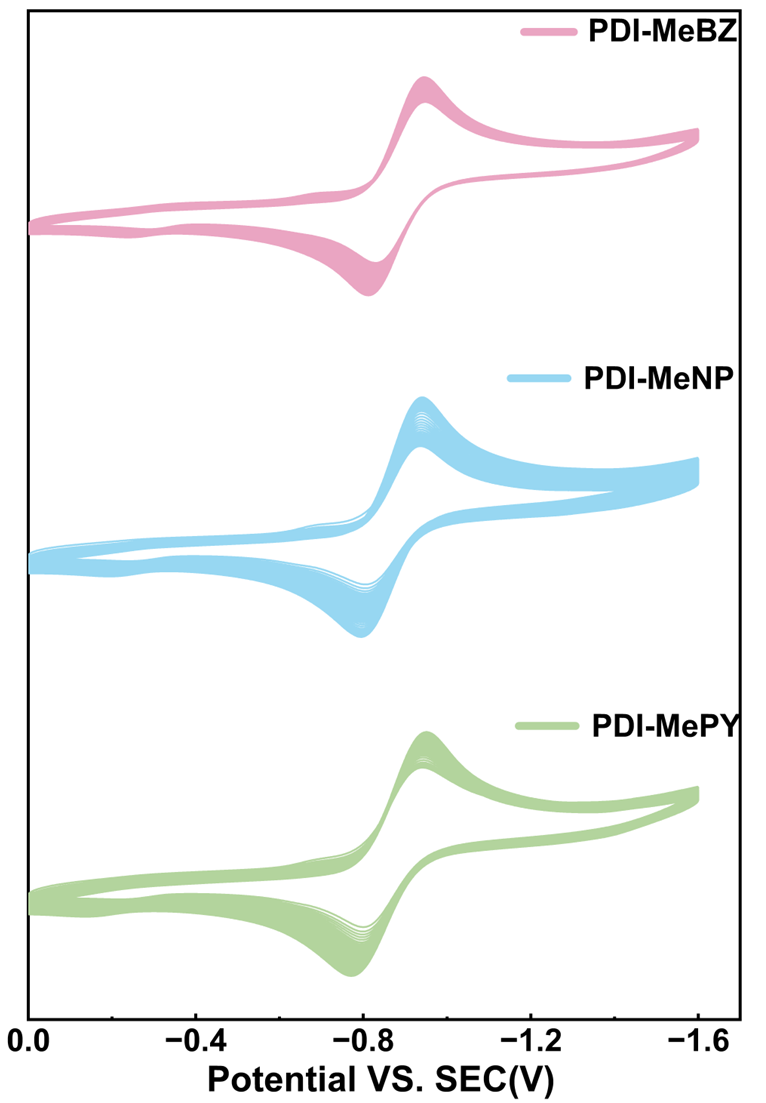


Figure S20. Cyclic voltammograms for the reduction of **PDI**–**MeBZ**, **PDI**–**MeNP**, and **PDI**–**MePY** in DMF solution (1 mM) containing 0.1 M TBAP as a supporting electrolyte at room temperature under an argon atmosphere (scan rate = 50 mV s⁻¹), recorded over 200 consecutive cycles.


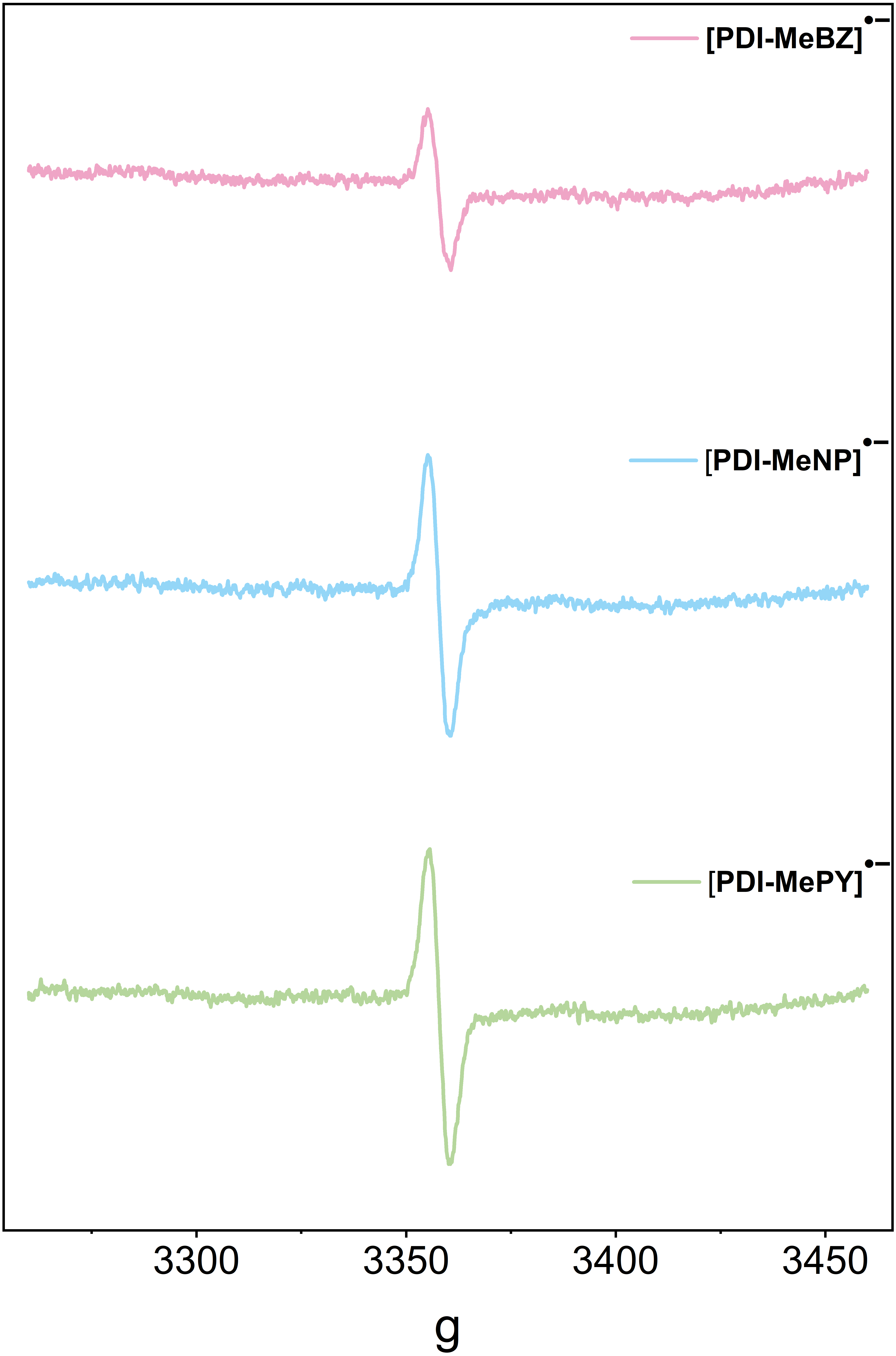


Figure S21. Continuous-wave (CW) EPR spectra of [**PDI**–**MeBZ**]**^•−^**, [**PDI**–**MeNP**]**^•−^**, and
[**PDI**–**MePY**]**^•−^** (2 mM in DMF at room temperature) measured immediately after synthesis, confirming the formation and persistence of radical anions.


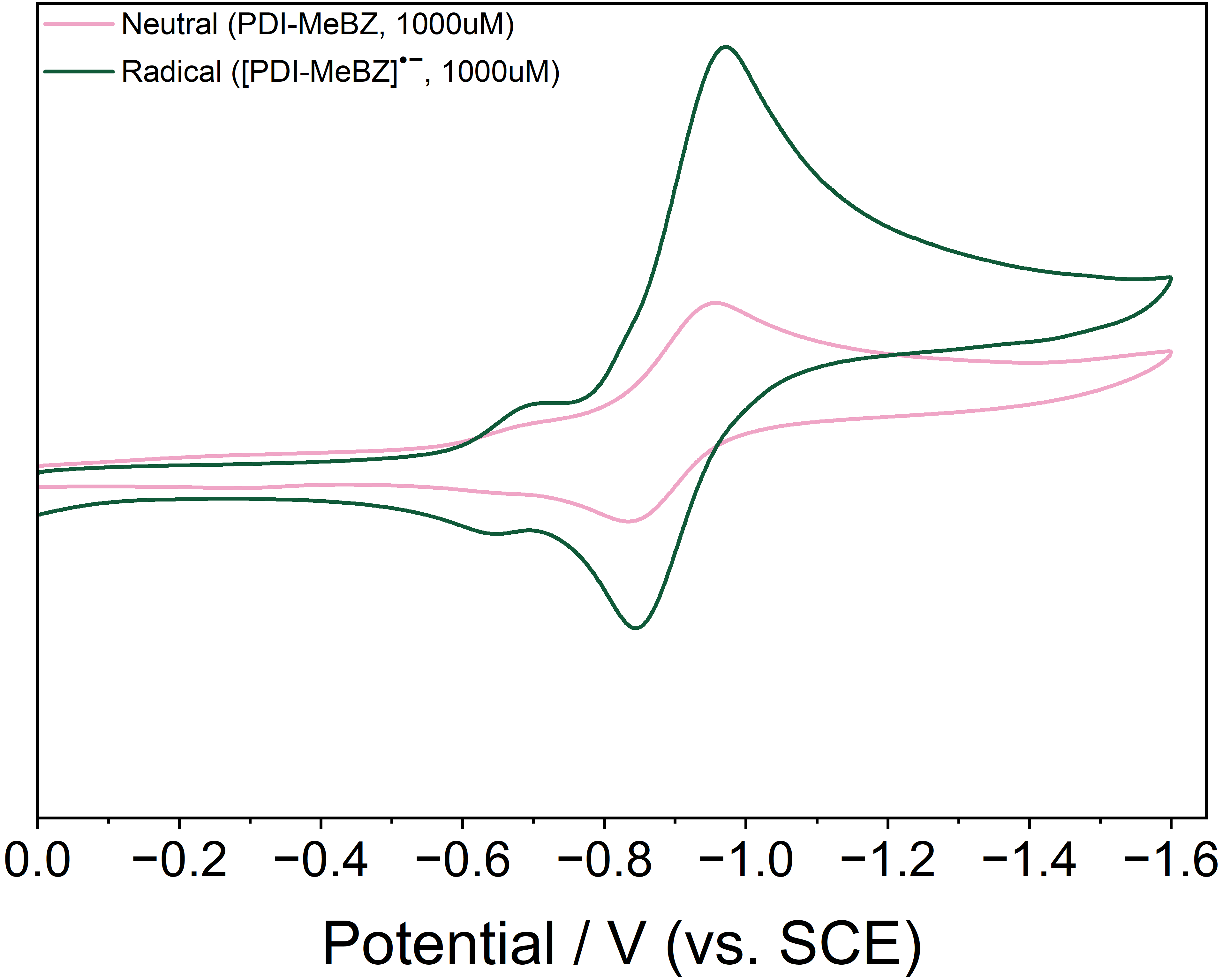


**(A)**

**(B)**


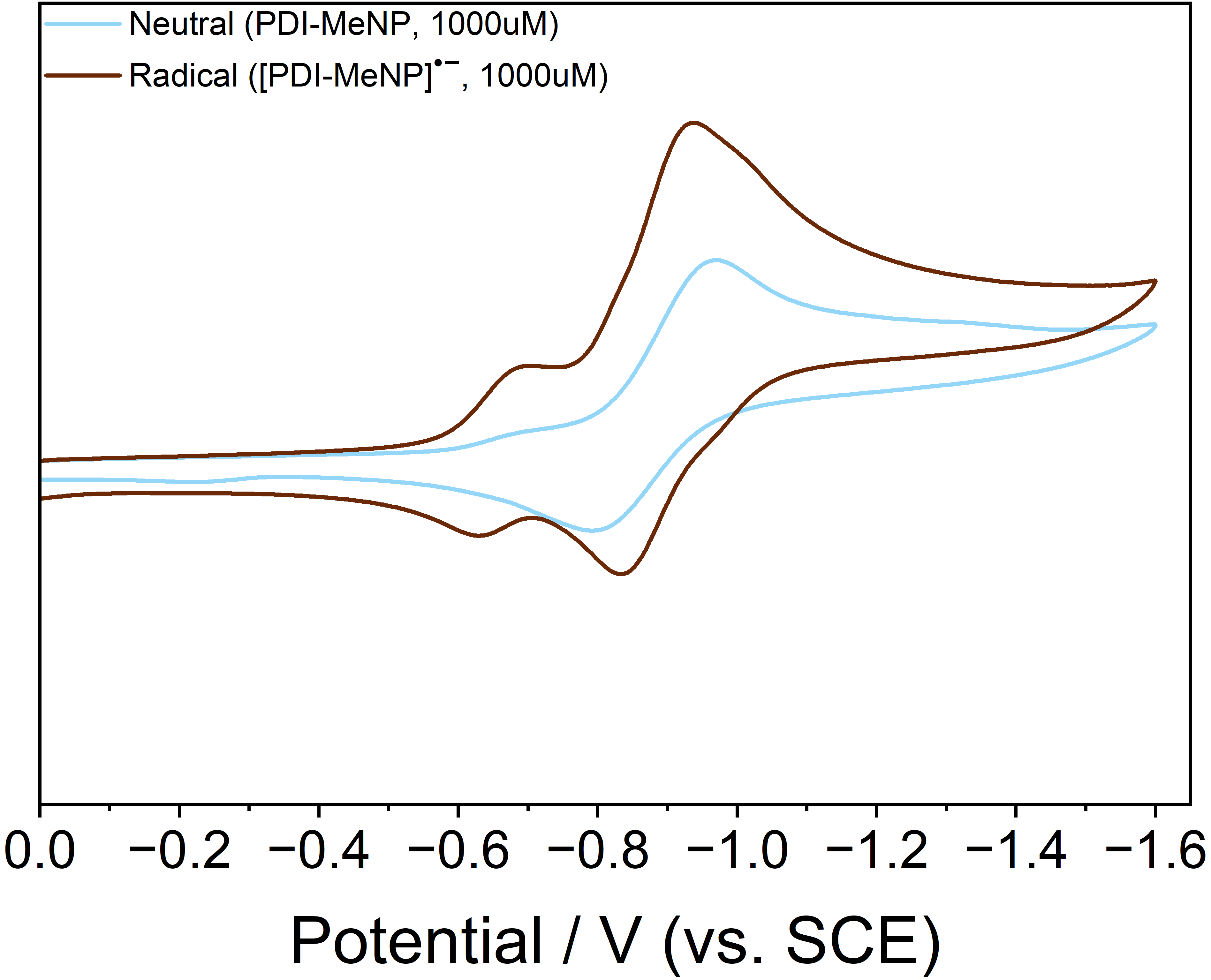


**(C)**


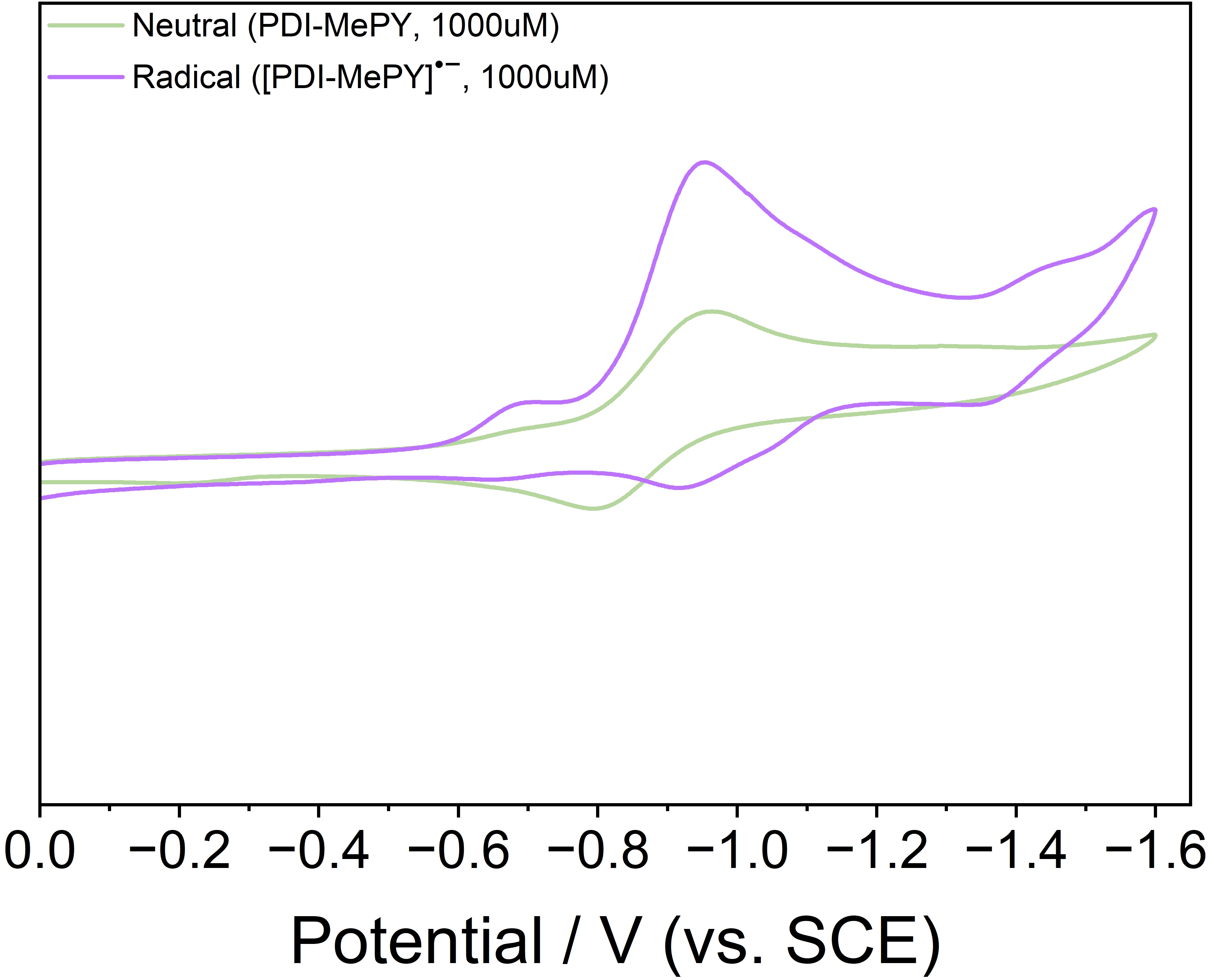


Figure S22. Comparison of cyclic voltammograms for the reduction of the neutral and chemically generated radical anion states of (A) **PDI**–**MeBZ**, [**PDI**–**MeBZ**]**^•−^**, (B) **PDI**–**MeNP**, [**PDI**–**MeNP**]**^•−^**, and (C) **PDI**–**MePY**, [**PDI**–**MePY**]**^•−^** in DMF solution (1 mM) containing 0.1 M TBAP as a supporting electrolyte at room temperature under an argon atmosphere (scan rate = 50 mV s^−1^).


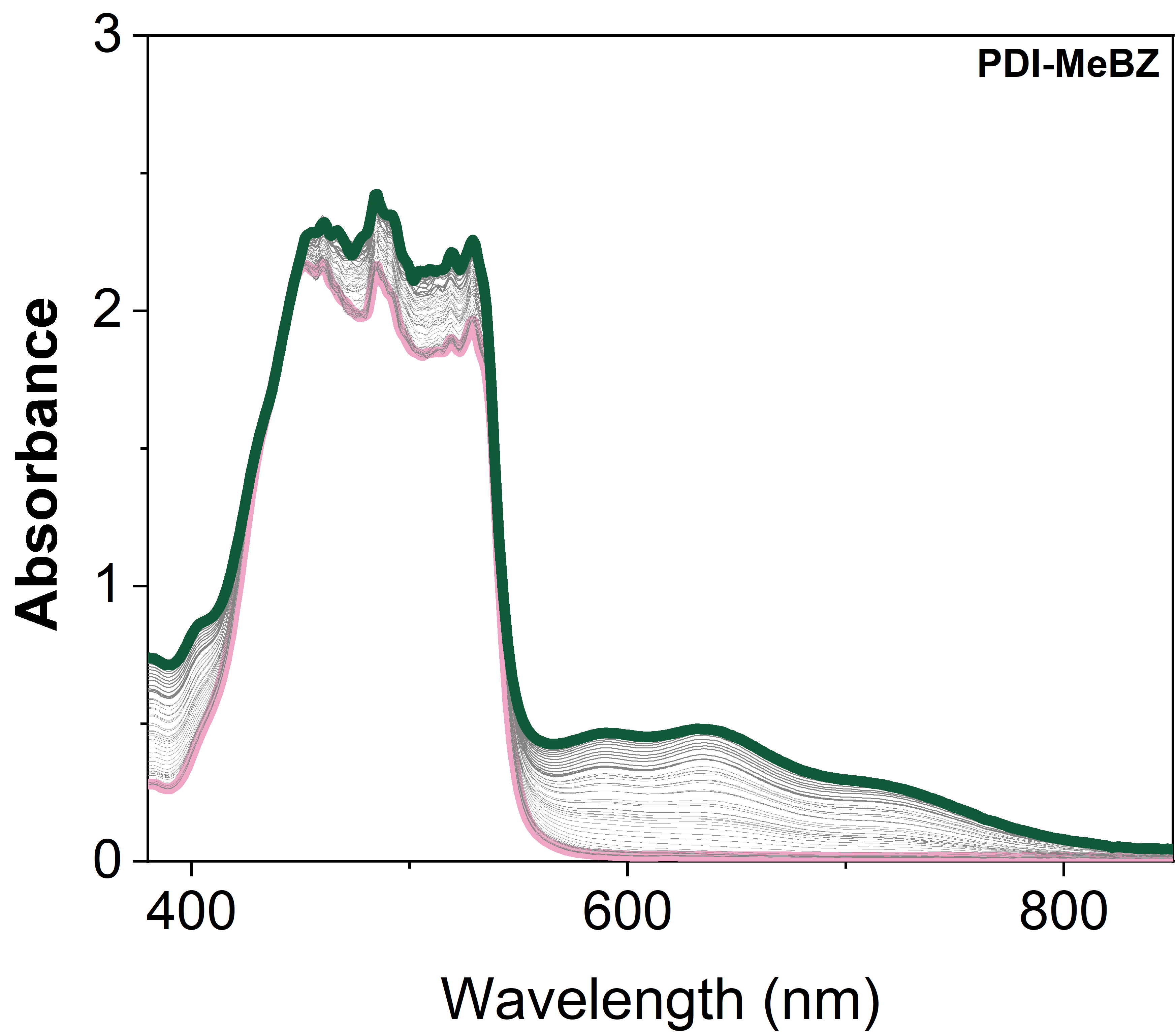


**(A)**


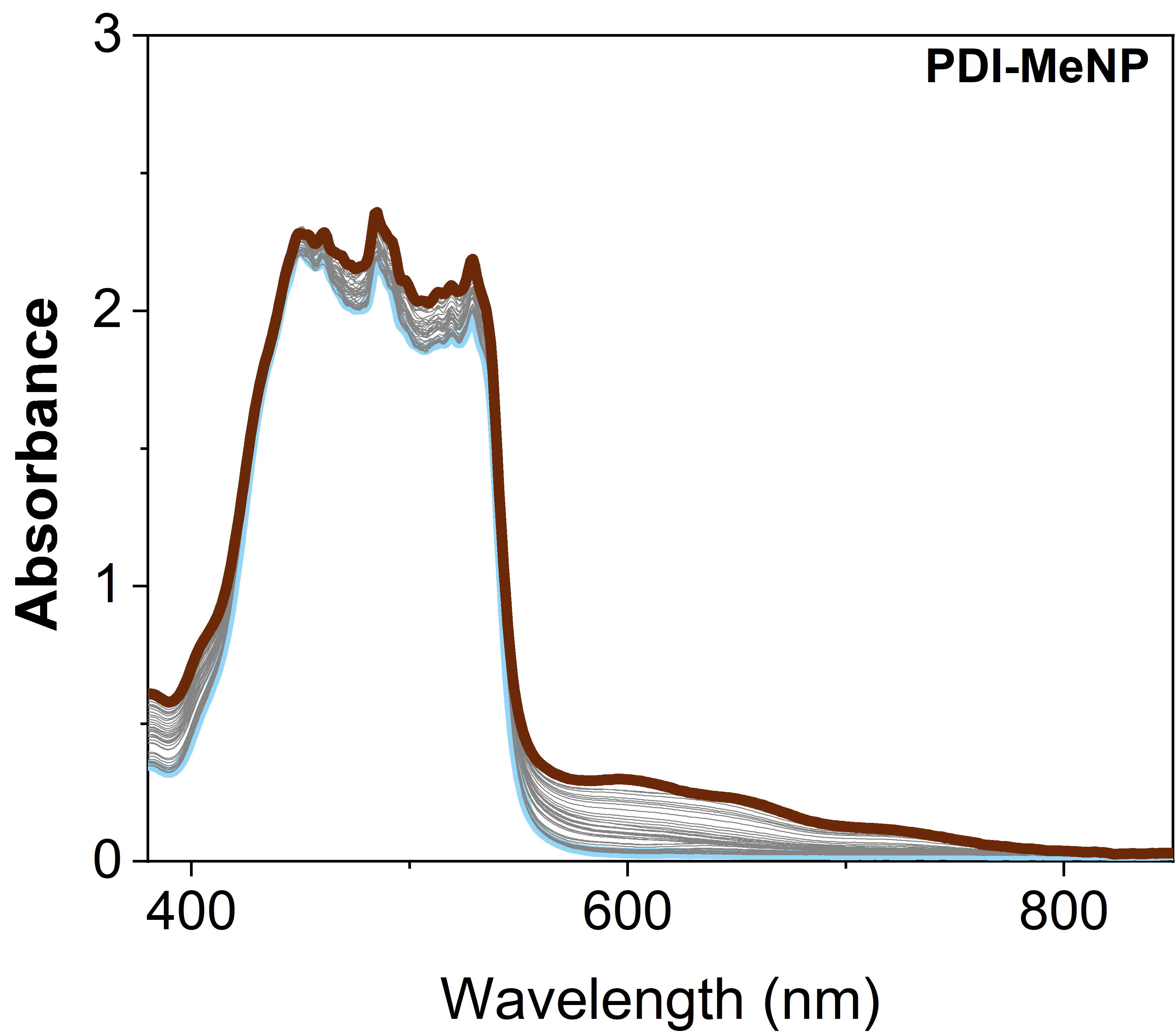


**(B)**


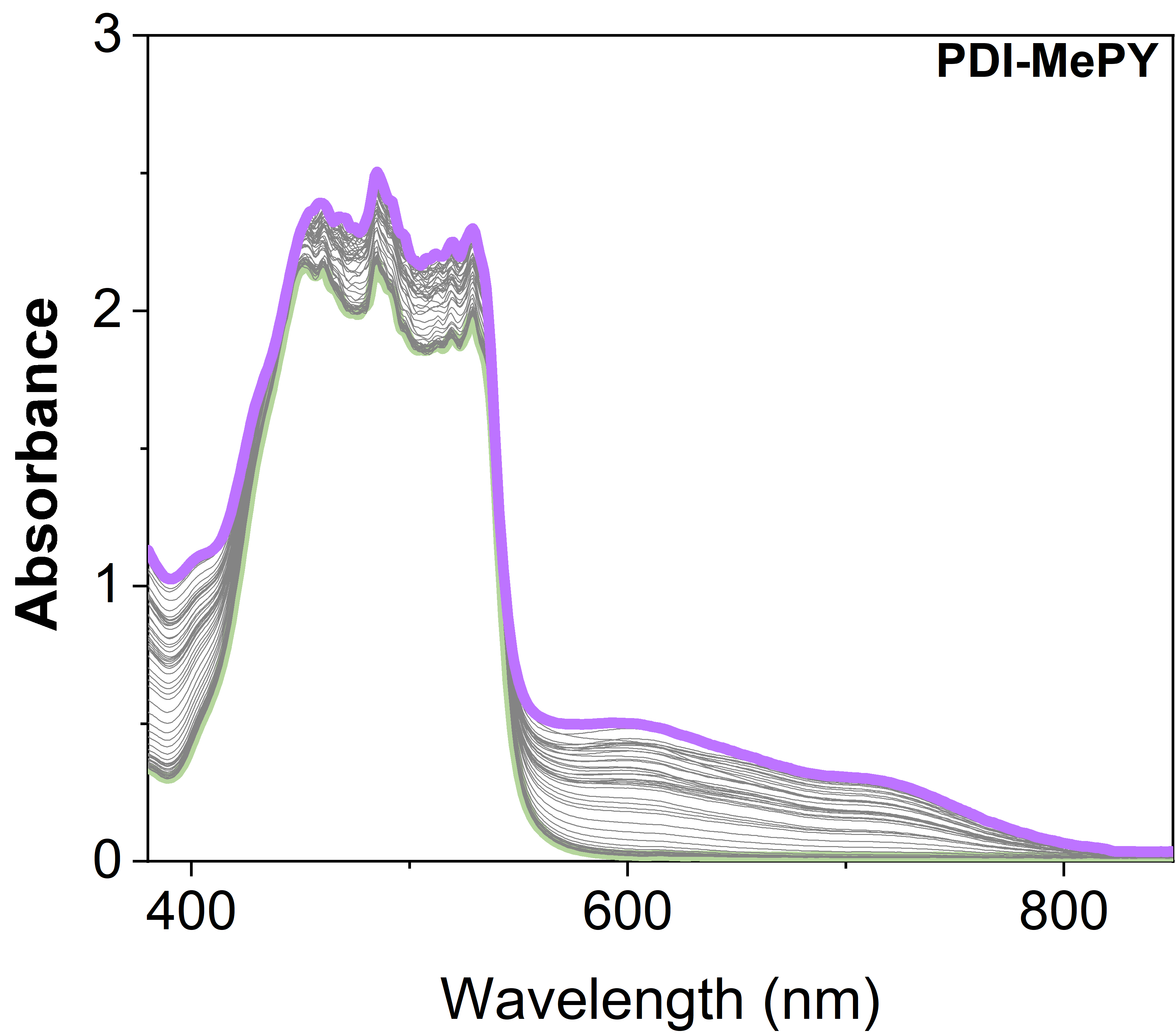


**(C)**

Figure S23. UV-vis absorption spectral change during the electrochemical reduction from the neutral species to the corresponding radical anion species (1 mM, 0.1 M TBAP in THF): (A) **PDI**–**MeBZ** to [**PDI**–**MeBZ**]**^•−^**, (B) **PDI**–**MeNP** to [**PDI**–**MeNP**]**^•−^**, and (C) **PDI**–**MePY** to [**PDI**–**MePY**]**^•−^**.


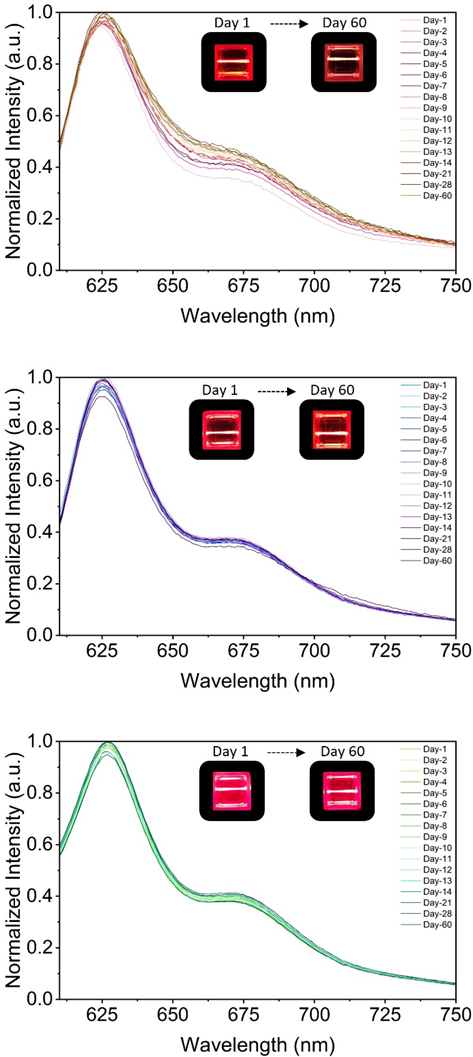


**(C)**

**(B)**

**(A)**

Figure S24. Doublet emission stability of chemically generated [**PDI**–**MeR**]**^•−^** radical anions and corresponding photographs of emissive solution in a quartz cell under 610 nm laser excitation monitored from Day 1 to Day 60: (A) [**PDI**–**MeBZ**]**^•−^**, (B) [**PDI**–**MeNP**]**^•−^**, and (C) [**PDI**–**MePY**]**^•−^**.

**Calculation Details**

Quantum chemical calculation were carried out using Gaussian 16 software package.^[7]^ The ground-state geometry of **PDI**–**MeR** and [**PDI**–**MeR**]**^•−^** has been optimized at the density function theory (DFT) level. Full geometry optimizations in their ground singlet and doublet state were performed under the B3LYP function with the 6-31G(d,p) basis set for **PDI**–**MeR**, and under the B3LYP function with the 6-31+G(d,p) basis set for [**PDI**–**MeR**]**^•−^**. All of the Cartesian coordinates for optimized structure of **PDI**–**MeR** (Table S4, S6, and S8) and [**PDI**–**MeR**]**^•−^** (Table S5, S7, and S9) are also summarized. The frontier molecular orbitals (Figure S27-S32) were generated at an isovalue of 0.02 a.u.

On basis of the geometry optimization at ground state, time-dependent density functional theory (TDDFT) at B3LYP/6-31+G(d) was used to calculate 50 vertical excitation and oscillator strengths, and the simulated absorption spectra and transition assignments were obtained by the GaussSum program. Afterwards, analyzing wavefunction by Multiwfn, electron-hole distribution^[8]^ could be obtained. The spin density maps obtained from ca. 1770000 grid points for [**PDI**–**MeR**]**^•−^** used Multiwfn (version 3.8)^[9]^ and VMD (version 1.9.3)^[10]^ for analysis and visualization.

To conduct an in‐depth analysis of the aromaticity characteristics, anisotropy of the induced current density (AICD)^[11]^ and gauge-including magnetically induced current (GIMIC)^[12]^ calculations were performed using the CSGT method (CSGT/B3LYP/6-31G(d)), based on the optimized ground-state geometries. In both calculations, the external magnetic field was applied along the z-axis, perpendicular to the molecular plane. AICDplots, rendered by POV-Ray (version 3.7) software^[13]^, represent the density of delocalized electrons. The current density vectors (arrows on the AICDisosurface) indicate a diatropic or paratropic ring current depending on the clockwise or counterclockwise direction, respectively. The magnetically induced current pathways were visualized using Paraview^[14]^.


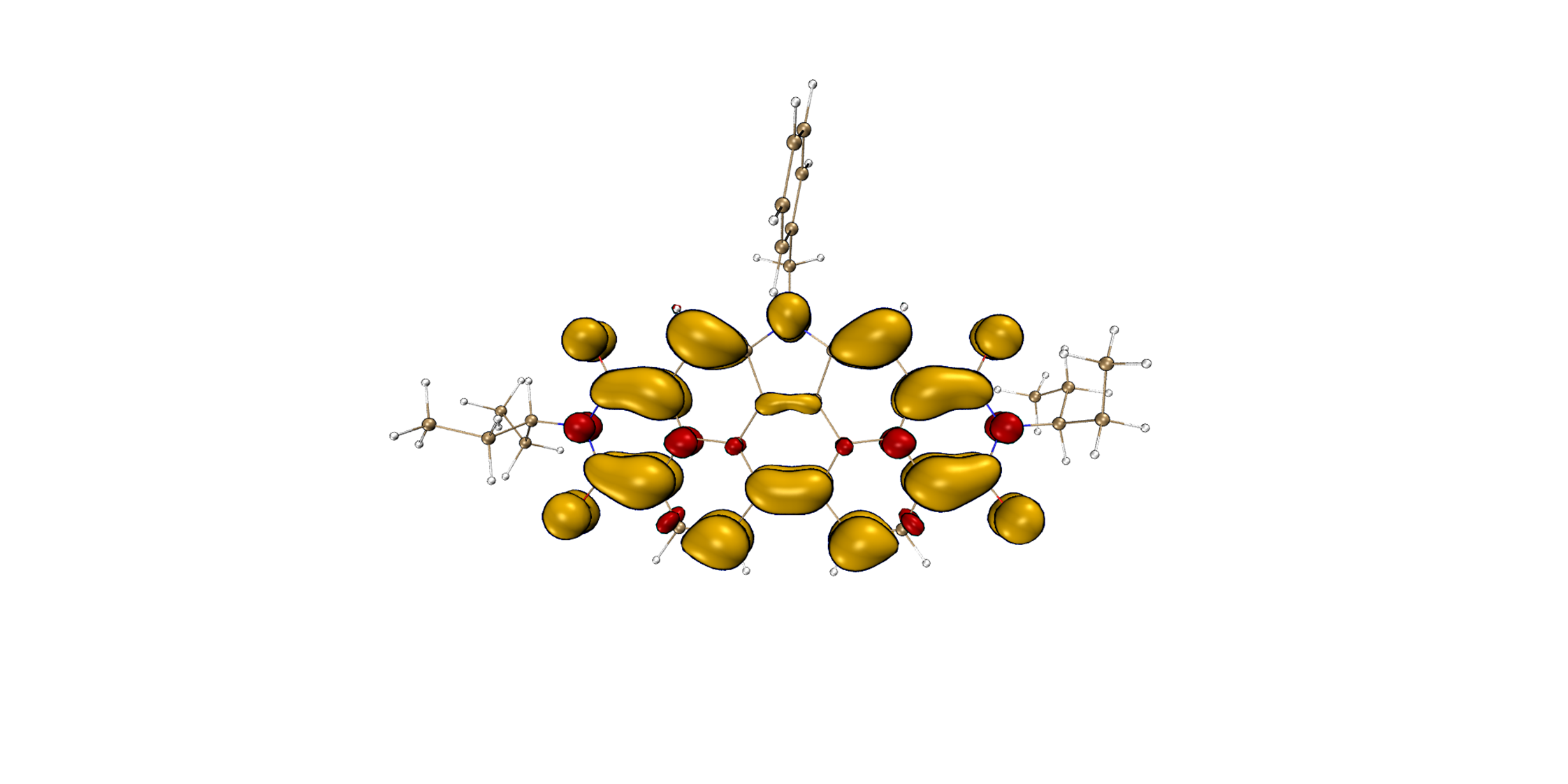


**(A)**


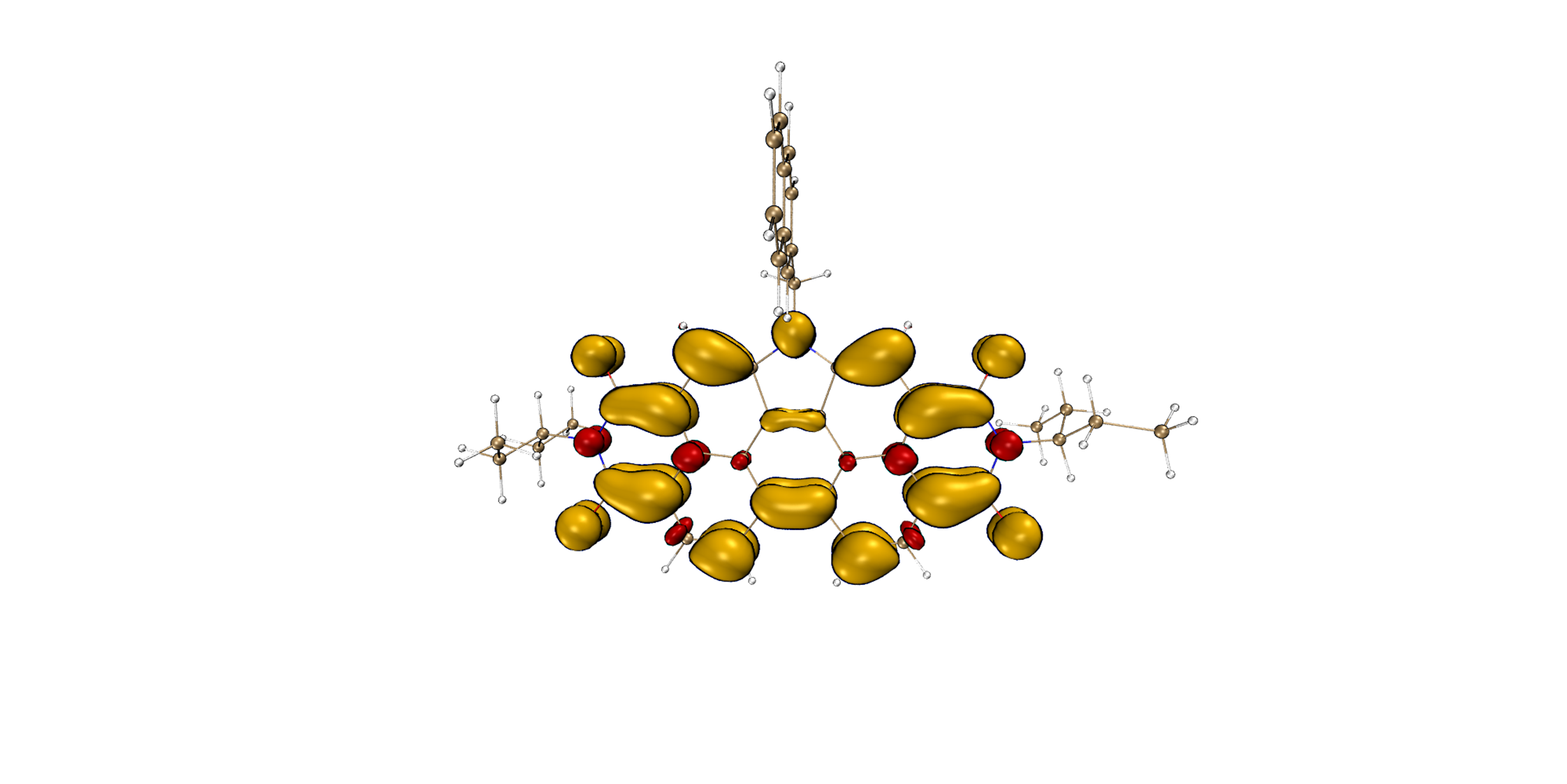

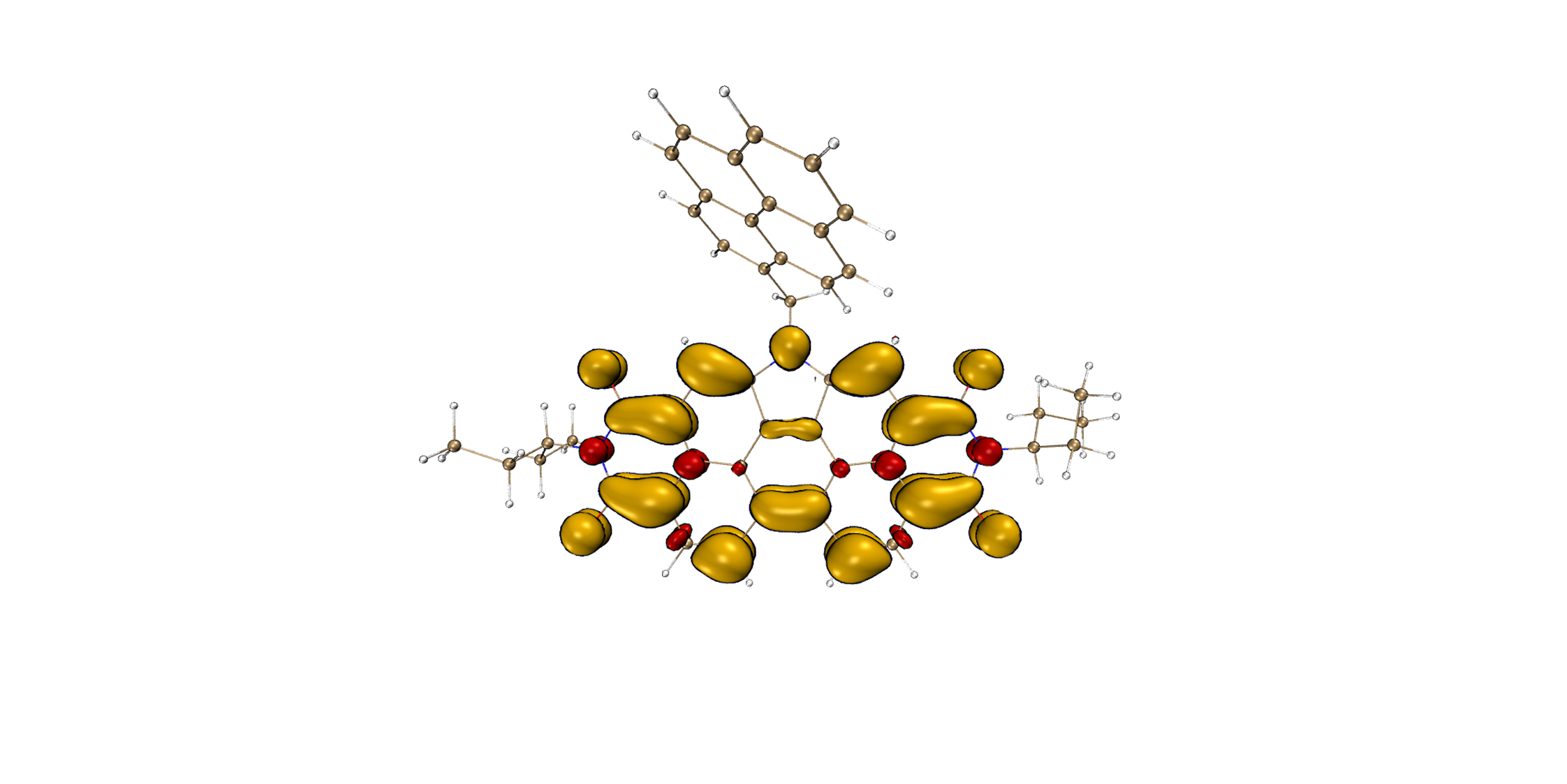
Figure S25. Spin density maps of radical anion states calculated at B3LYP/6-31+G(d,p) level (isovalue = 0.001 a.u.). (A) [**PDI**–**MeBZ**]**^•−^**, (B) [**PDI**–**MeNP**]**^•−^**, and (C) [**PDI**–**MePY**]**^•−^**.

**(B)**

**(C)**


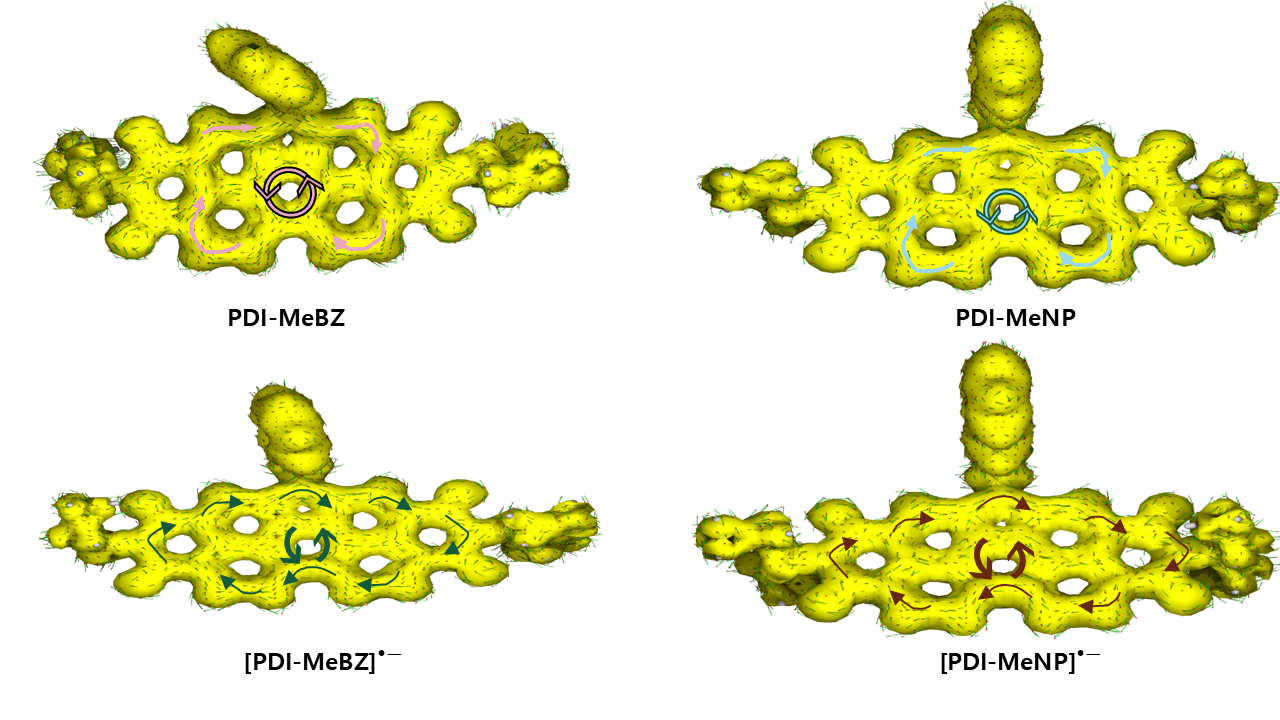


**(C)**

**(D)**

**(B)**

**(A)**

Figure S26. AICDplots of neutral and radical anion states calculated at B3LYP/6-31G(d,p) and B3LYP/6-31G(d) level (isovalue = 0.05). (A) **PDI**–**MeBZ**, (B) [**PDI**–**MeBZ**]**^•−^**, (C) **PDI**–**MeNP**, and (D) [**PDI**–**MeNP**]**^•−^**.


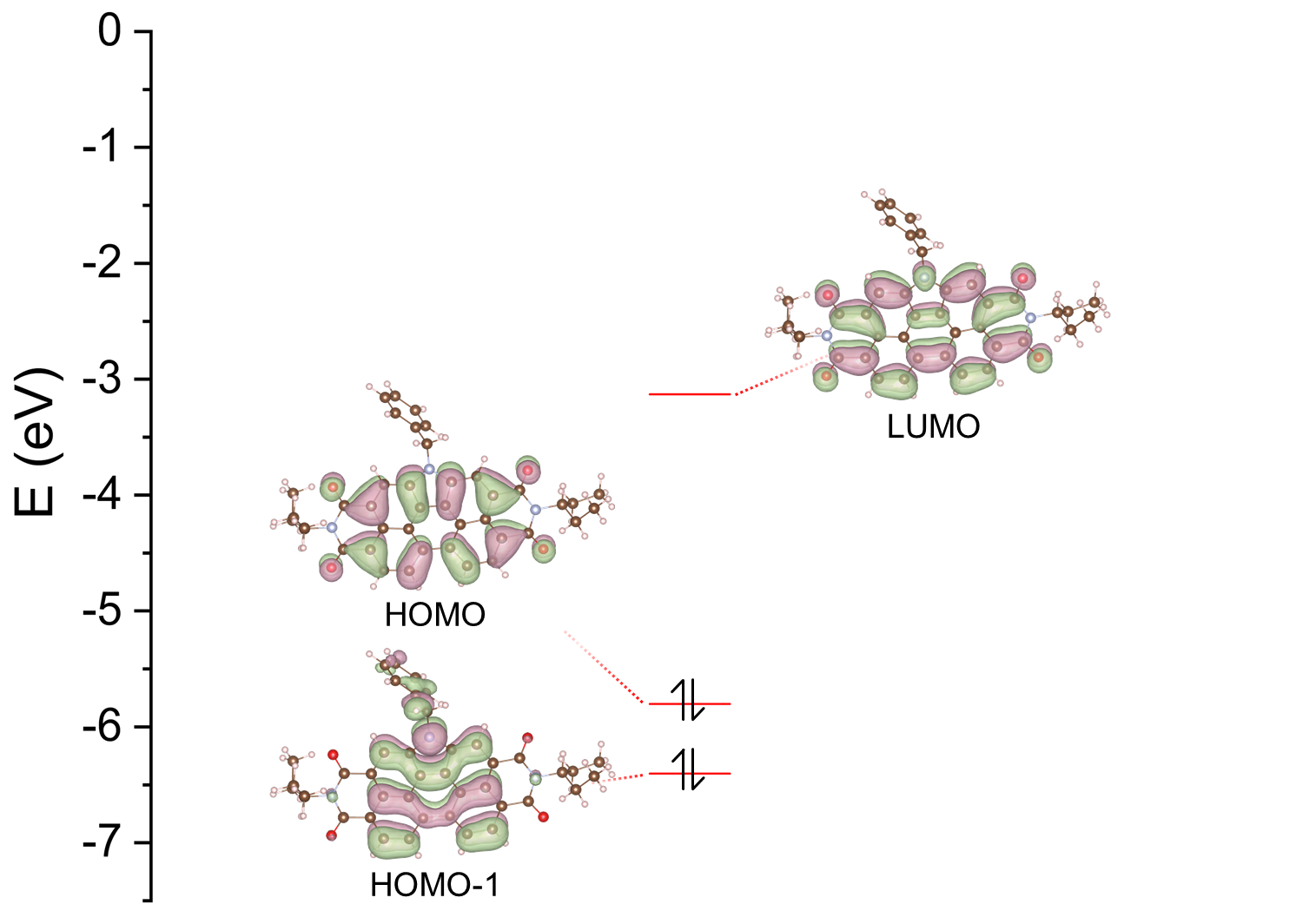

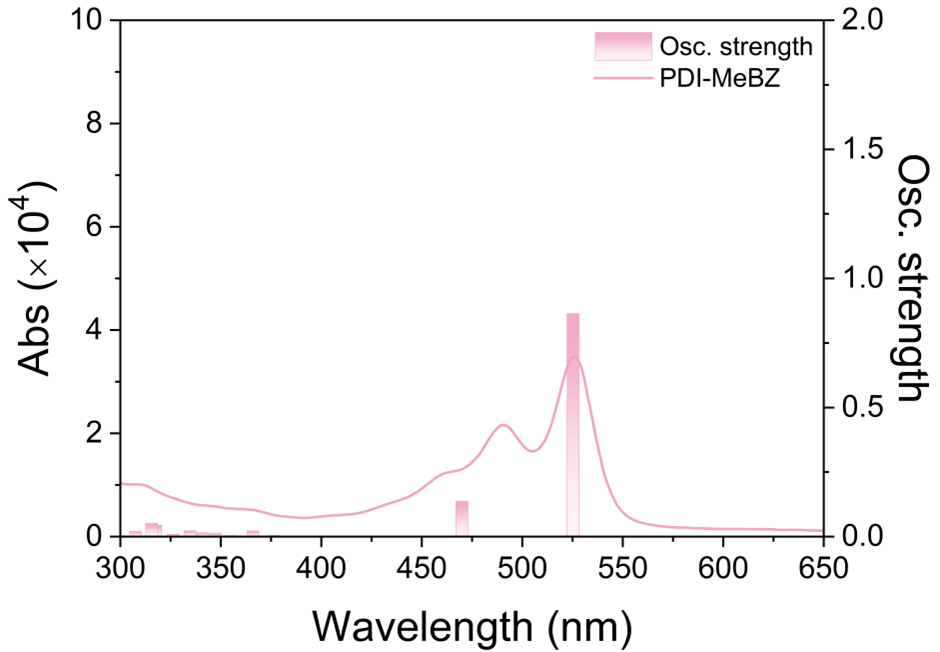
Figure S27. (A) Energy levels and isodensity plots (isovalue = 0.02 a.u.) of **PDI**–**MeBZ**,
(B) Electronic transition of simulated (lines)/experimental (sticks) absorption spectra of **PDI**–**MeBZ**.

**(A)**

**(B)**


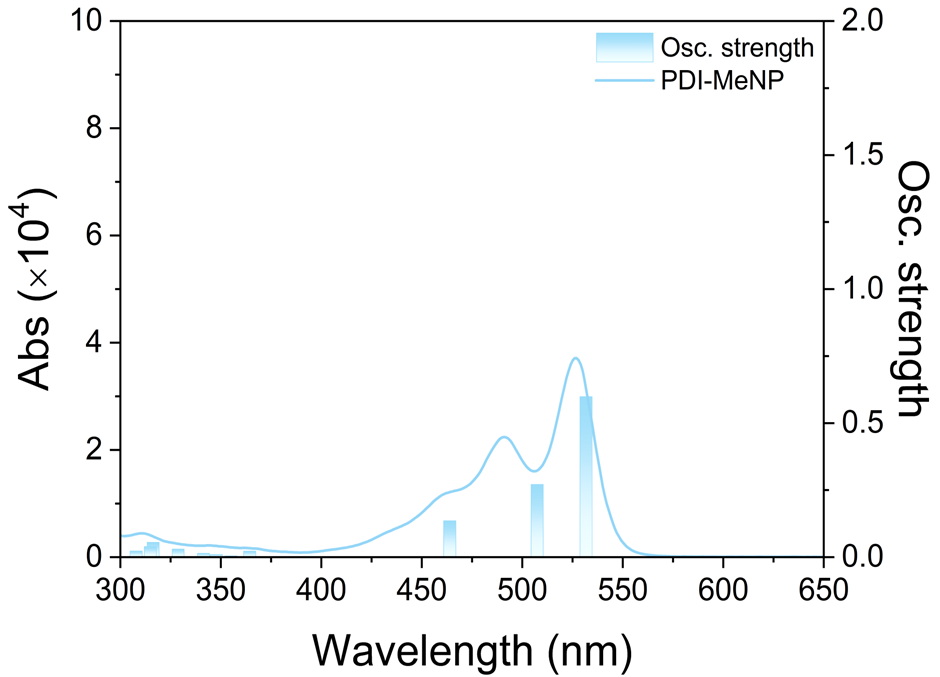
**
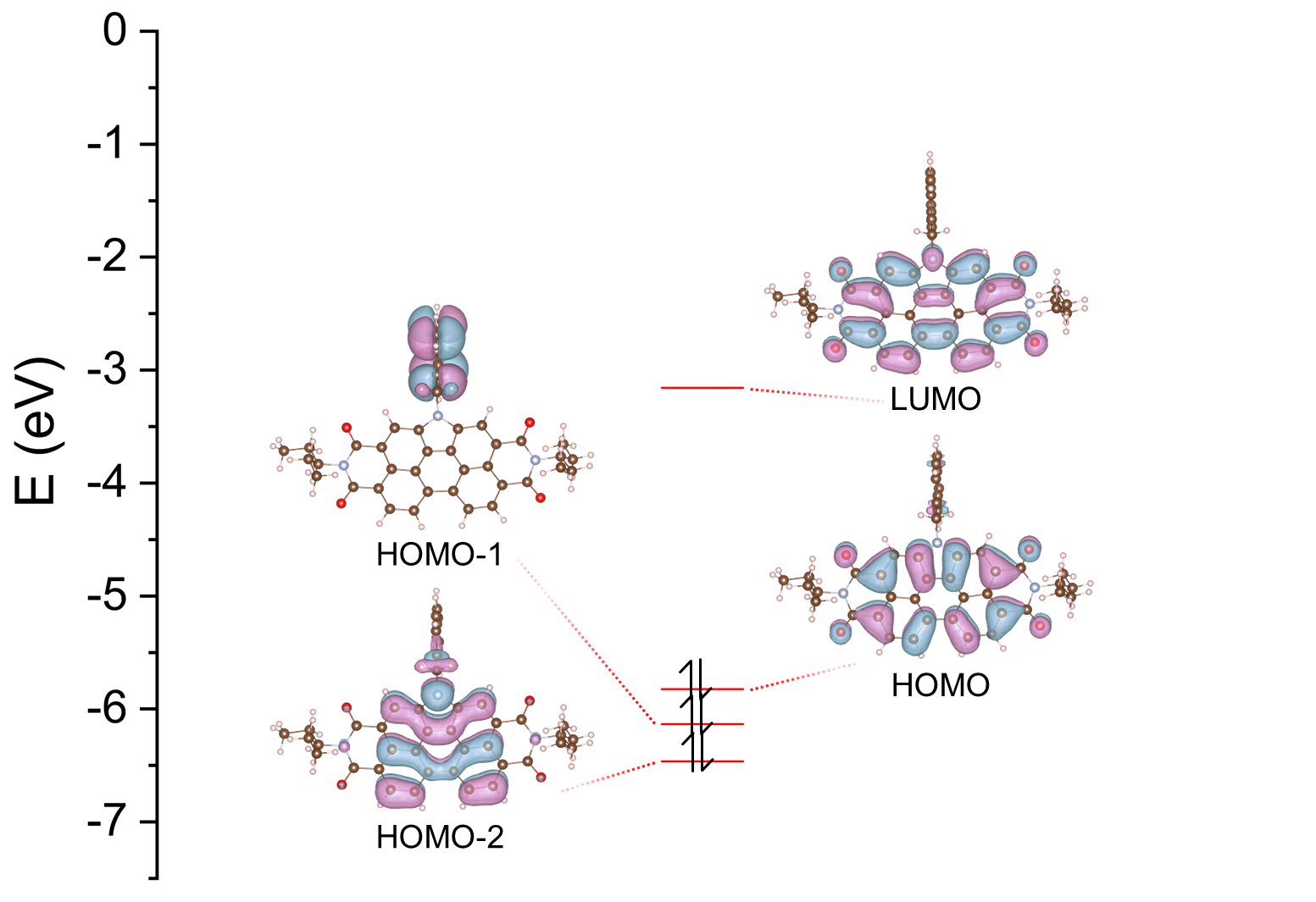
**Figure S28. (A) Energy levels and isodensity plots (isovalue = 0.02 a.u.) of **PDI**–**MeNP**,
(B) Electronic transition of simulated (lines)/experimental (sticks) absorption spectra of **PDI**–**MeNP**.

**(B)**

**(A)**


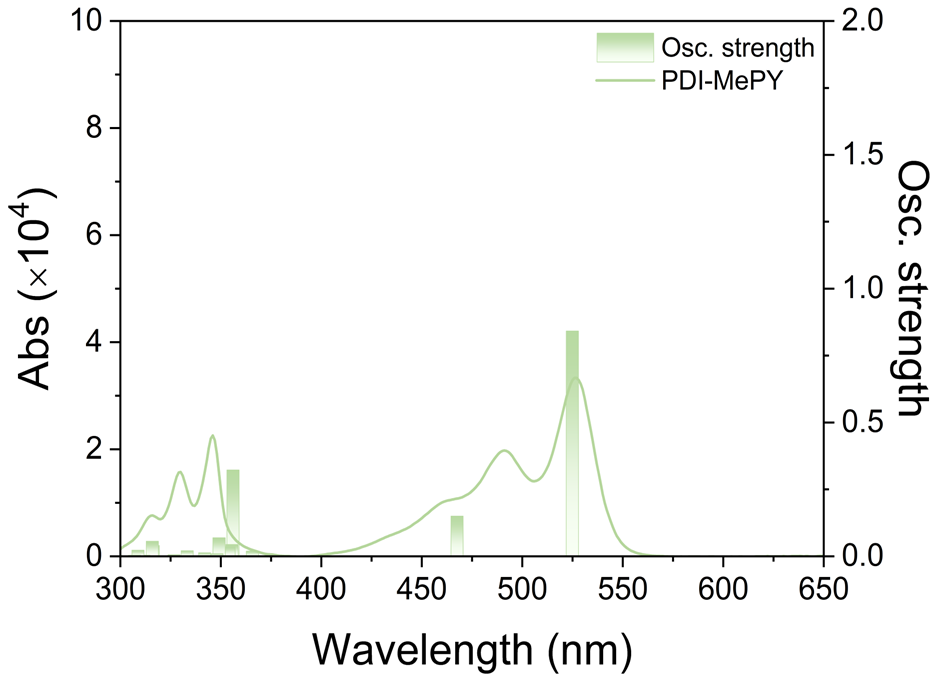
**
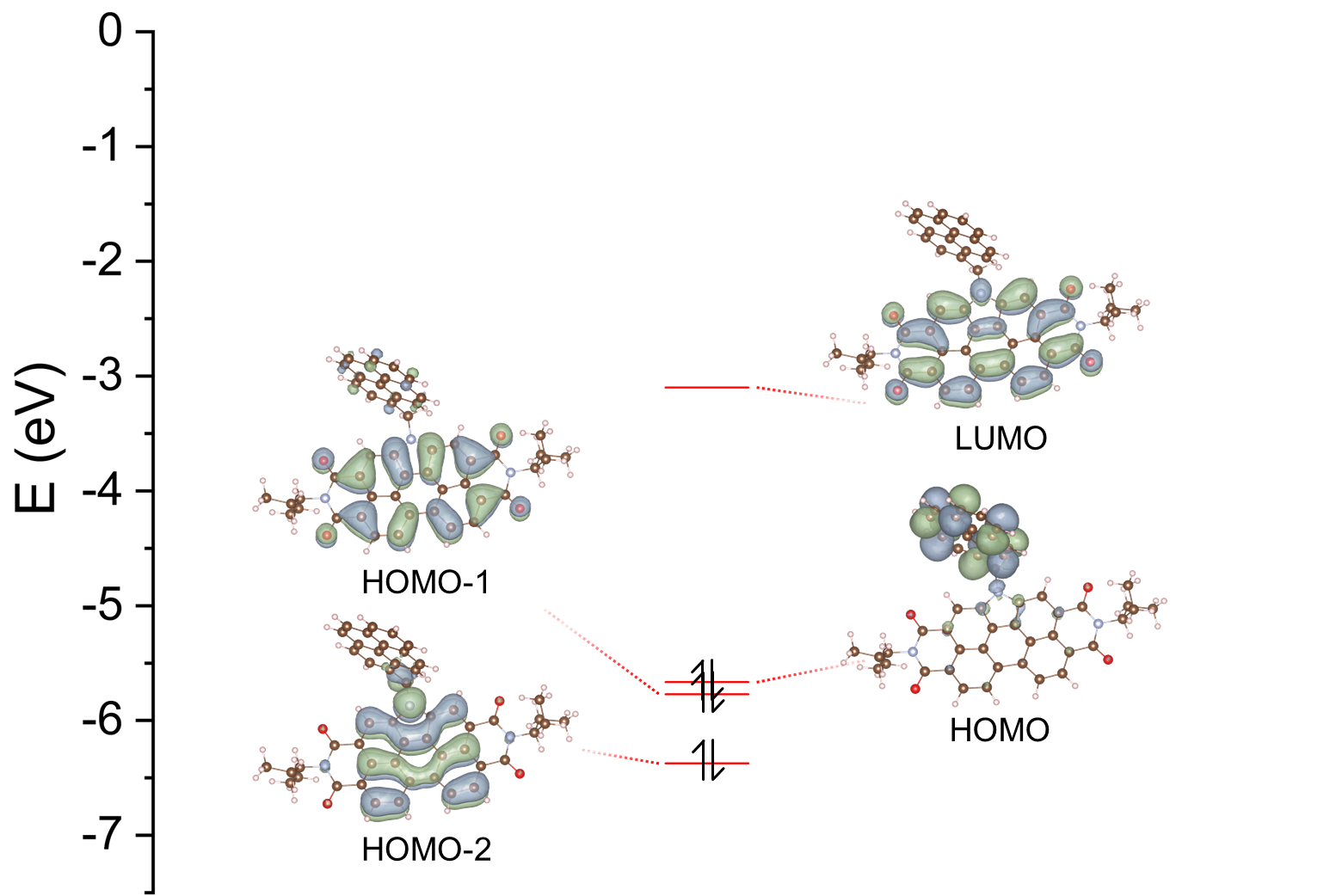
**Figure S29. (A) Energy levels and isodensity plots (isovalue = 0.02 a.u.) of **PDI**–**MePY**,
(B) Electronic transition of simulated (lines)/experimental (sticks) absorption spectra of **PDI**–**MePY**.

**(A)**

**(B)**


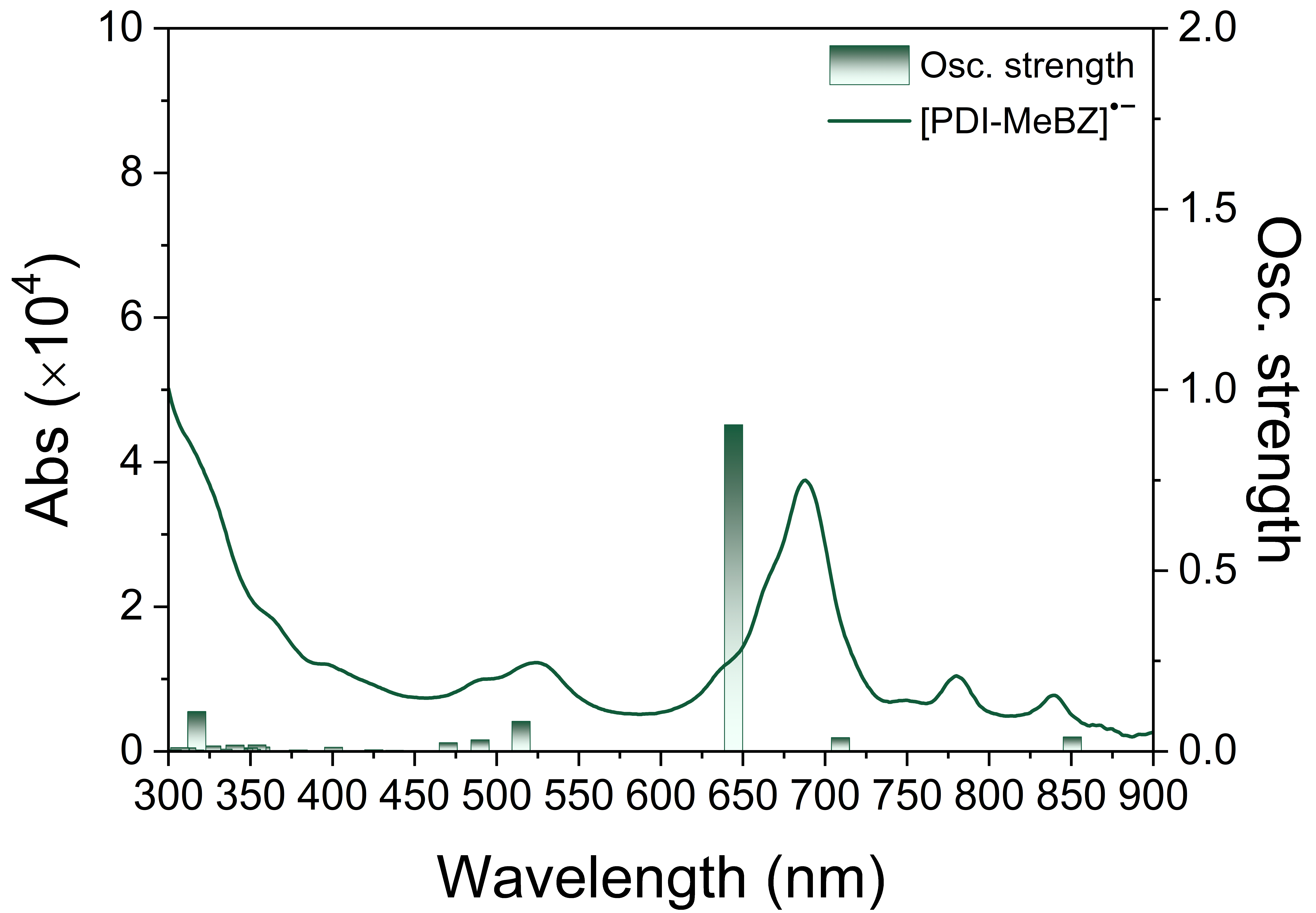
**
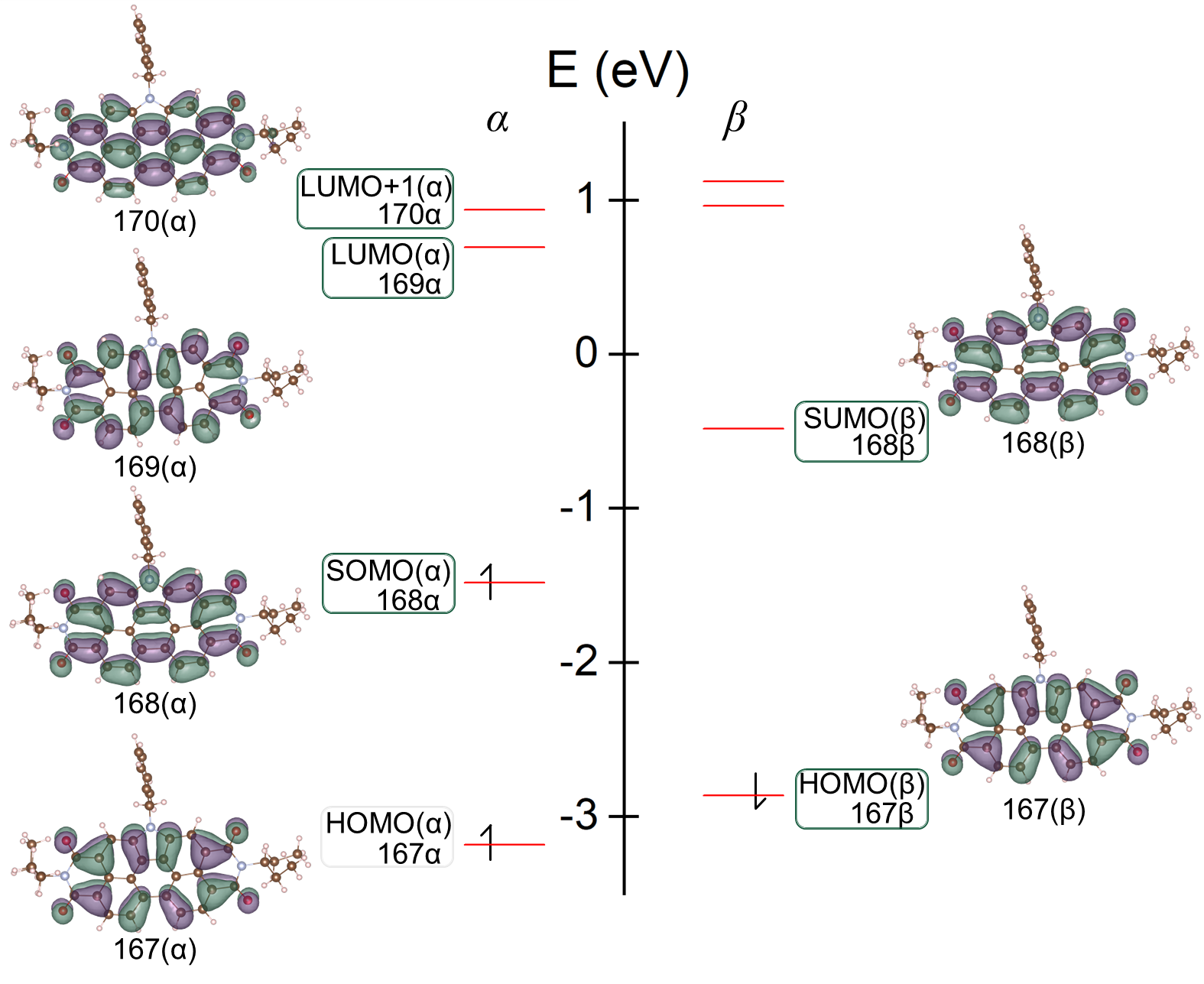
**Figure S30. (A) Energy levels and isodensity plots (isovalue = 0.02 a.u.) of [**PDI**–**MeBZ**]**^•−^**, (B) Electronic transition of simulated (lines)/experimental (sticks) absorption spectra of [**PDI**–**MeBZ**]**^•−^**.
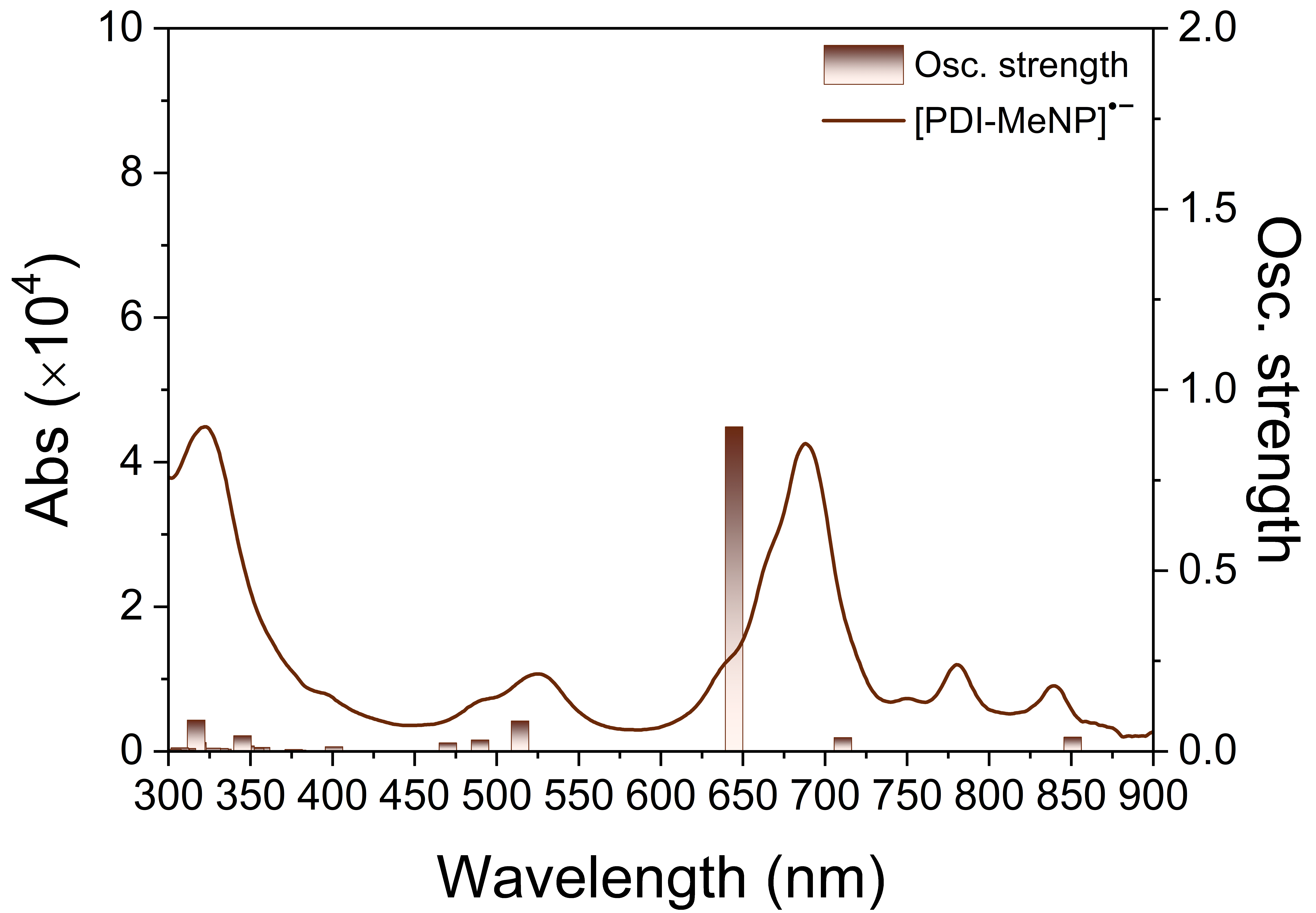
**
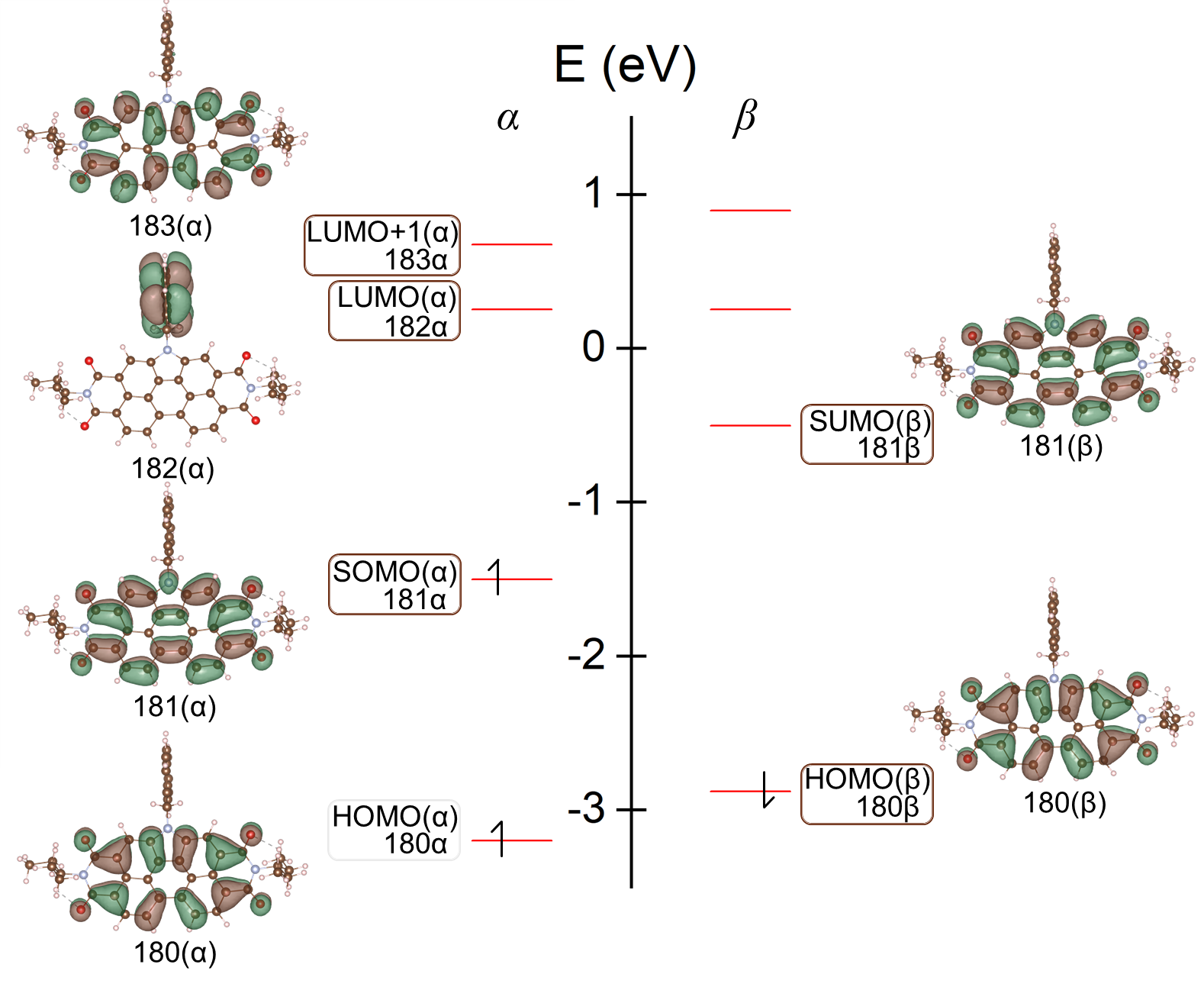
**Figure S31. (A) Energy levels and isodensity plots (isovalue = 0.02 a.u.) of [**PDI**–**MeNP**]**^•−^**, (B) Electronic transition of simulated (lines)/experimental (sticks) absorption spectra of [**PDI**–**MeNP**]**^•−^**.

**(B)**

**(A)**

**(B)**

**(A)**


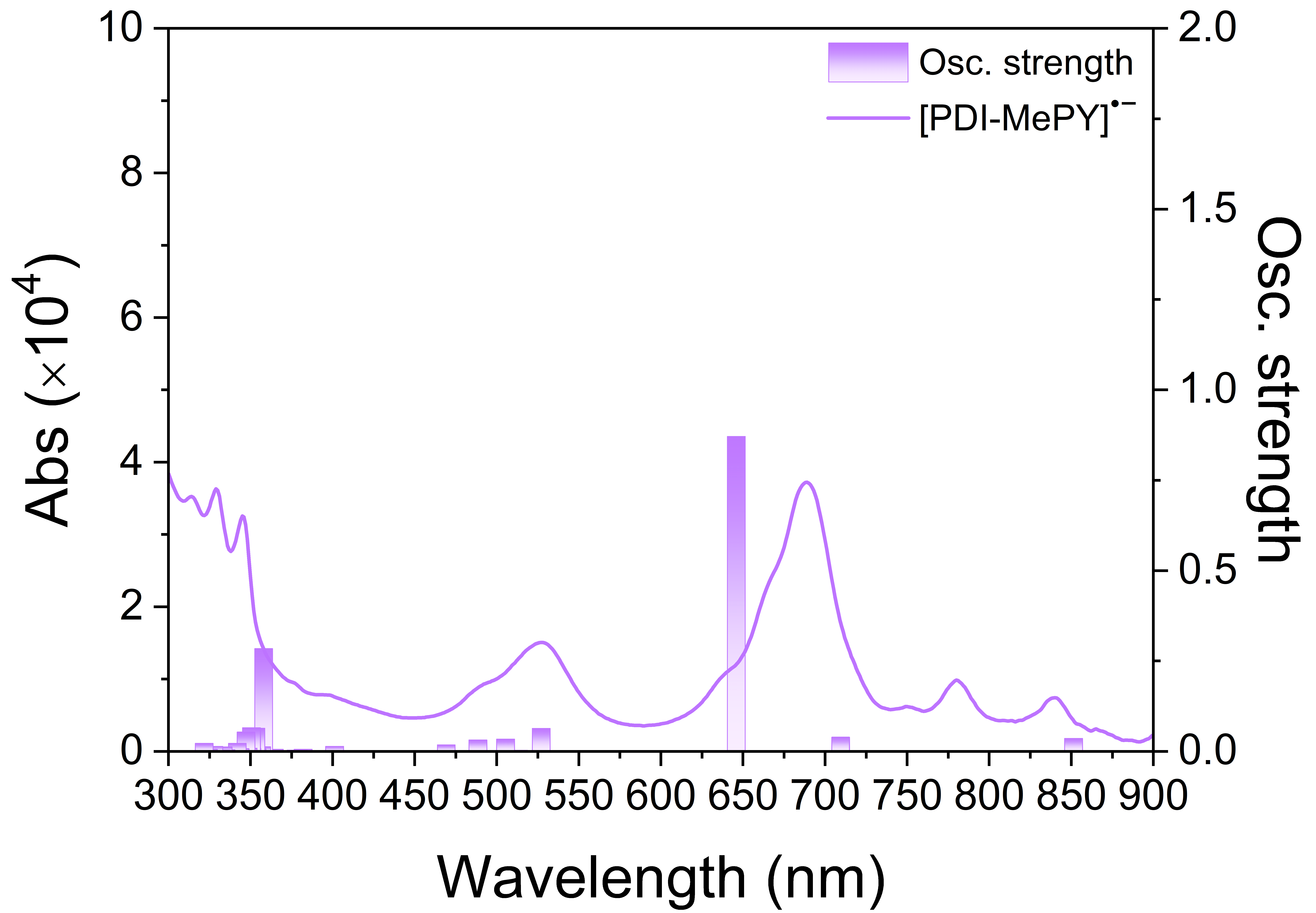
**
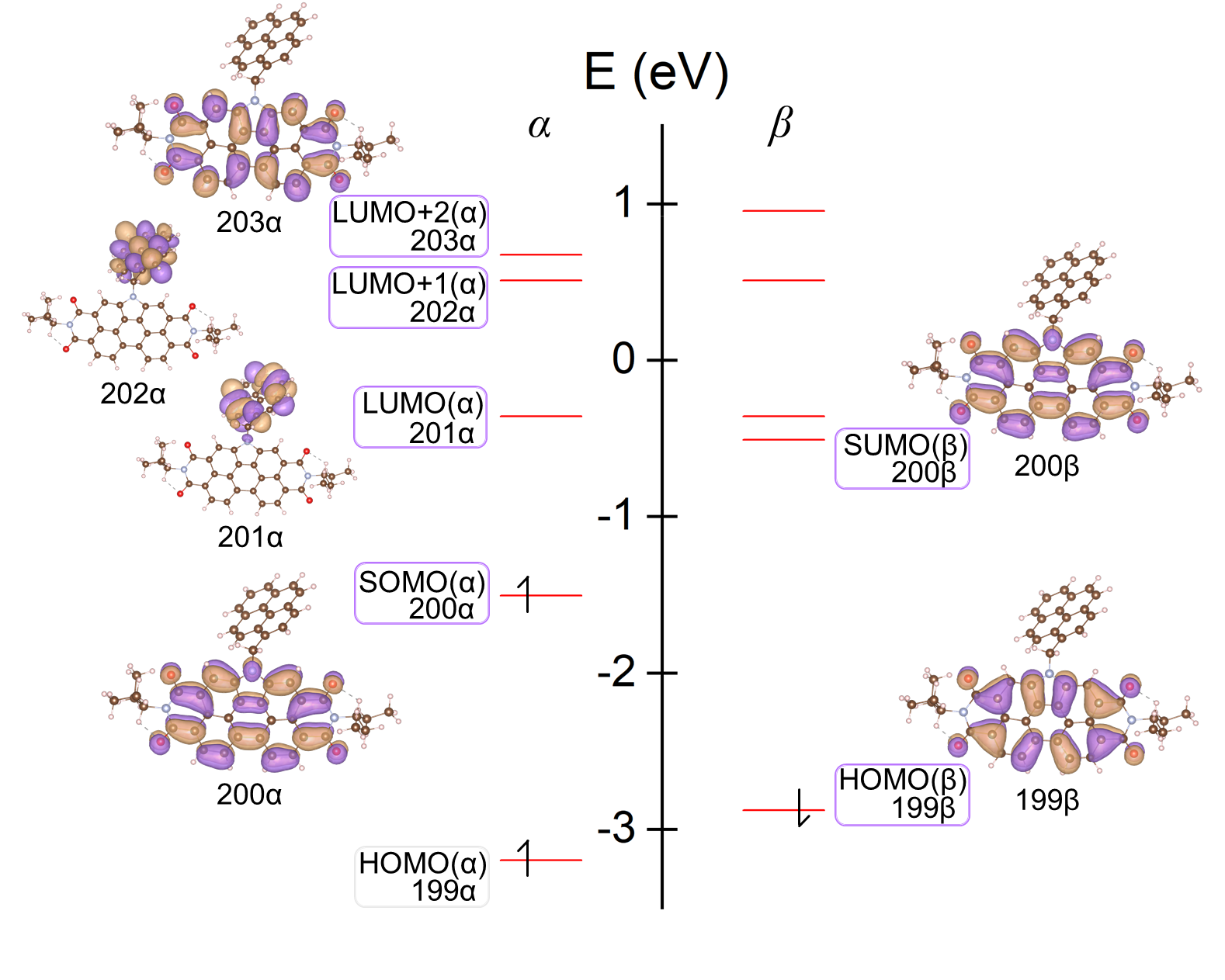
**Figure S32. (A) Energy levels and isodensity plots (isovalue = 0.02 a.u.) of [**PDI**–**MePY**]**^•−^**, (B) Electronic transition of simulated (lines)/experimental (sticks) absorption spectra of [**PDI**–**MePY**]**^•−^**.

**(A)**

**(B)**

Table S4. Cartesian coordinates for optimized structure for **PDI**–**MeBZ**

Symbolic Z-matrix:

Charge = 0 Multiplicity = 1

| Atom | X | Y | Z | Atom | X | Y | Z |
| --- | --- | --- | --- | --- | --- | --- | --- |
| C | 2.47098 | 1.48606 | 0.686398 | C | -0.88079 | 4.188508 | 0.235968 |
| C | 1.085231 | 1.202371 | 0.626335 | C | -0.33644 | 4.273067 | -1.05225 |
| C | 0.739413 | -0.09226 | 0.244828 | C | -0.89838 | 5.125205 | -2.00044 |
| C | 1.569977 | -1.14879 | -0.08417 | C | -2.00979 | 5.906134 | -1.67198 |
| C | 2.947306 | -0.86289 | -0.02301 | C | -2.5563 | 5.828586 | -0.39194 |
| C | 3.36482 | 0.453822 | 0.36265 | C | -1.99388 | 4.971319 | 0.556437 |
| C | 1.004731 | -2.41331 | -0.44988 | H | 2.890357 | 2.446815 | 0.965351 |
| C | 1.945285 | -3.40604 | -0.76405 | H | 1.619907 | -4.40038 | -1.05409 |
| C | 3.327619 | -3.14379 | -0.71244 | H | 4.035531 | -3.92759 | -0.96034 |
| C | 3.841521 | -1.89478 | -0.34771 | H | -3.19462 | 1.924637 | 0.902386 |
| C | -0.63032 | -0.21185 | 0.236728 | H | -3.19773 | -4.55859 | -1.00116 |
| C | -1.26059 | -1.39681 | -0.09923 | H | -0.7365 | -4.60608 | -1.06852 |
| C | -0.48094 | -2.54324 | -0.45785 | H | 7.197621 | 0.959604 | 0.575048 |
| C | -1.20201 | 1.002126 | 0.609518 | H | -7.15246 | -2.46032 | 0.19379 |
| C | -2.61697 | 1.043389 | 0.648542 | H | 7.166673 | -1.49568 | 1.776278 |
| C | -3.31299 | -0.13065 | 0.323592 | H | 8.399917 | -1.79505 | 0.579236 |
| C | -2.66702 | -1.35416 | -0.05169 | H | 7.67413 | -0.98581 | -1.72019 |
| C | -3.36533 | -2.52384 | -0.38511 | H | 7.10512 | 0.676973 | -1.85766 |
| C | -2.63914 | -3.66453 | -0.74551 | H | 9.408372 | -0.97047 | 2.712606 |
| C | -1.23199 | -3.68353 | -0.78204 | H | 9.683713 | 0.24747 | 1.464686 |
| C | 4.822467 | 0.727269 | 0.415502 | H | 8.406493 | 0.486283 | 2.660108 |
| C | 5.297533 | -1.6342 | -0.29722 | H | 9.510864 | 0.599318 | -2.37554 |
| C | -4.79949 | -0.11231 | 0.351466 | H | 9.295673 | 1.495383 | -0.86725 |
| C | -4.84344 | -2.52206 | -0.34448 | H | 9.881958 | -0.17547 | -0.83663 |
| N | -5.47375 | -1.319 | 0.066728 | H | -7.49569 | 0.307012 | 1.419318 |
| N | 5.696291 | -0.34273 | 0.102749 | H | -8.64633 | -1.02489 | 1.379502 |
| O | 5.259064 | 1.831545 | 0.719622 | H | -7.20965 | -1.51608 | -1.98583 |
| O | 6.124034 | -2.49227 | -0.58153 | H | -8.69384 | -1.24798 | -1.10046 |
| O | -5.41582 | 0.912768 | 0.618038 | H | -7.52673 | -0.96241 | 3.58541 |
| O | -5.49856 | -3.51193 | -0.64528 | H | -7.04628 | -2.44034 | 2.73995 |
| N | -0.12887 | 1.886912 | 0.856643 | H | -5.93488 | -1.06644 | 2.834803 |
| C | 7.163677 | -0.04919 | 0.161348 | H | -8.16901 | 0.733117 | -2.45349 |
| C | -6.97306 | -1.3853 | 0.141213 | H | -8.04889 | 1.183645 | -0.7476 |
| C | 7.903941 | -0.99699 | 1.139139 | H | -6.58551 | 0.919812 | -1.68727 |
| C | 7.742259 | 0.006973 | -1.26705 | H | -0.85241 | 3.313339 | 2.20355 |
| C | 8.907862 | -0.26558 | 2.040496 | H | 0.746208 | 3.62278 | 1.542008 |
| C | 9.188055 | 0.509469 | -1.33344 | H | 0.526106 | 3.666121 | -1.31433 |
| C | -7.57669 | -0.77958 | 1.420036 | H | -0.46875 | 5.181907 | -2.99615 |
| C | -7.6458 | -0.92692 | -1.17039 | H | -2.44718 | 6.569061 | -2.41237 |
| C | -6.98598 | -1.34623 | 2.714595 | H | -3.42298 | 6.428012 | -0.1303 |
| C | -7.60518 | 0.561945 | -1.52996 | H | -2.42514 | 4.91168 | 1.552826 |
| C | -0.26008 | 3.279833 | 1.282478 |  |  |  |  |

Table S5. Cartesian coordinates for optimized structure for [**PDI**–**MeBZ**]**^•−^**

Symbolic Z-matrix:

Charge = -1 Multiplicity = 2

| Atom | X | Y | Z | Atom | X | Y | Z |
| --- | --- | --- | --- | --- | --- | --- | --- |
| C | 2.407078 | 1.402334 | 0.696294 | C | -0.43389 | 4.303112 | 0.228122 |
| C | 1.032765 | 1.158717 | 0.627592 | C | -0.24438 | 4.011526 | -1.12639 |
| C | 0.635938 | -0.12882 | 0.235099 | C | -0.39061 | 5.010616 | -2.09396 |
| C | 1.43555 | -1.21203 | -0.09203 | C | -0.72773 | 6.312865 | -1.71928 |
| C | 2.830421 | -0.97304 | -0.02074 | C | -0.92148 | 6.611437 | -0.36668 |
| C | 3.288614 | 0.324734 | 0.371311 | C | -0.77666 | 5.611934 | 0.596867 |
| C | 0.821029 | -2.45406 | -0.46088 | H | 2.851257 | 2.351819 | 0.974742 |
| C | 1.738711 | -3.49362 | -0.77807 | H | 1.37339 | -4.47366 | -1.07073 |
| C | 3.11626 | -3.28238 | -0.7204 | H | 3.796287 | -4.09183 | -0.96768 |
| C | 3.689857 | -2.04404 | -0.34651 | H | -3.22749 | 2.010431 | 0.917535 |
| C | -0.73485 | -0.20604 | 0.22343 | H | -3.41135 | -4.49637 | -1.02592 |
| C | -1.4019 | -1.37234 | -0.11416 | H | -0.96058 | -4.60465 | -1.0906 |
| C | -0.64644 | -2.53673 | -0.47251 | H | 7.106752 | 0.693833 | 0.588571 |
| C | -1.28148 | 1.028049 | 0.606484 | H | -7.30403 | -2.27857 | 0.140678 |
| C | -2.67552 | 1.115377 | 0.65256 | H | 6.997639 | -1.78557 | 1.773179 |
| C | -3.42381 | -0.05592 | 0.319282 | H | 8.263782 | -2.07274 | 0.609605 |
| C | -2.81552 | -1.2919 | -0.06279 | H | 7.553907 | -1.27411 | -1.69165 |
| C | -3.54524 | -2.45062 | -0.40124 | H | 7.019774 | 0.40092 | -1.84199 |
| C | -2.83059 | -3.61621 | -0.76786 | H | 9.217606 | -1.27652 | 2.783481 |
| C | -1.43739 | -3.67194 | -0.80375 | H | 9.529425 | -0.04372 | 1.554737 |
| C | 4.732822 | 0.544249 | 0.435167 | H | 8.218925 | 0.185331 | 2.717429 |
| C | 5.132471 | -1.84225 | -0.29115 | H | 9.434558 | 0.271757 | -2.33587 |
| C | -4.88713 | 0.002163 | 0.353543 | H | 9.213428 | 1.185104 | -0.83563 |
| C | -5.00049 | -2.41499 | -0.36244 | H | 9.770456 | -0.49641 | -0.7829 |
| N | -5.59724 | -1.18607 | 0.047753 | H | -7.57305 | 0.462046 | 1.444044 |
| N | 5.572686 | -0.55989 | 0.114283 | H | -8.76474 | -0.83997 | 1.36387 |
| O | 5.232788 | 1.632906 | 0.755012 | H | -7.32974 | -1.27638 | -2.00917 |
| O | 5.959458 | -2.72192 | -0.57029 | H | -8.8137 | -1.00435 | -1.11773 |
| O | -5.50288 | 1.038009 | 0.640222 | H | -7.64123 | -0.86304 | 3.575785 |
| O | -5.70944 | -3.38652 | -0.66118 | H | -7.20356 | -2.33336 | 2.686385 |
| N | -0.17118 | 1.880575 | 0.860337 | H | -6.05362 | -0.99347 | 2.81193 |
| C | 7.041245 | -0.31619 | 0.179991 | H | -8.24931 | 0.999592 | -2.42683 |
| C | -7.09062 | -1.20815 | 0.119578 | H | -8.12164 | 1.408153 | -0.70654 |
| C | 7.753707 | -1.27927 | 1.165221 | H | -6.662 | 1.136445 | -1.64597 |
| C | 7.637694 | -0.27999 | -1.24303 | H | -1.08659 | 3.324841 | 2.030973 |
| C | 8.736278 | -0.56215 | 2.104385 | H | 0.658159 | 3.466735 | 1.884615 |
| C | 9.094788 | 0.196567 | -1.29636 | H | 0.01362 | 3.000415 | -1.42419 |
| C | -7.68781 | -0.62171 | 1.412293 | H | -0.2421 | 4.766141 | -3.14208 |
| C | -7.758 | -0.70115 | -1.17917 | H | -0.84354 | 7.087257 | -2.47248 |
| C | -7.11313 | -1.24008 | 2.692131 | H | -1.19099 | 7.619556 | -0.06314 |
| C | -7.69015 | 0.796256 | -1.50466 | H | -0.93639 | 5.849665 | 1.646904 |
| C | -0.25582 | 3.249425 | 1.318011 |  |  |  |  |

Table S6. Cartesian coordinates for optimized structure for **PDI**–**MeNP**

Symbolic Z-matrix:

Charge = 0 Multiplicity = 1

| Atom | X | Y | Z | Atom | X | Y | Z |
| --- | --- | --- | --- | --- | --- | --- | --- |
| C | 2.557482 | 0.64826 | 1.185767 | C | -0.08132 | 6.093037 | -0.10658 |
| C | 1.158247 | 0.473013 | 1.06477 | C | -0.04901 | 4.834553 | -0.78801 |
| C | 0.735408 | -0.66292 | 0.379042 | C | -0.04043 | 3.634433 | -0.025 |
| C | 1.502111 | -1.66027 | -0.19693 | C | -0.08984 | 7.290042 | -0.86973 |
| C | 2.893599 | -1.48442 | -0.0734 | C | -0.06747 | 7.246963 | -2.24579 |
| C | 3.38855 | -0.32949 | 0.617571 | C | -0.0355 | 6.002623 | -2.92048 |
| C | 0.863464 | -2.76441 | -0.84847 | C | -0.02648 | 4.824444 | -2.20785 |
| C | 1.744224 | -3.71095 | -1.39336 | H | 3.028704 | 1.488488 | 1.683771 |
| C | 3.139572 | -3.55501 | -1.28677 | H | 1.361526 | -4.58653 | -1.90872 |
| C | 3.725833 | -2.46311 | -0.63783 | H | 3.800382 | -4.30059 | -1.71608 |
| C | -0.6394 | -0.69465 | 0.355166 | H | -3.07422 | 1.347744 | 1.576852 |
| C | -1.33879 | -1.72597 | -0.2463 | H | -3.45816 | -4.46812 | -1.84295 |
| C | -0.62758 | -2.79877 | -0.87445 | H | -1.00396 | -4.6408 | -1.95049 |
| C | -1.13766 | 0.419976 | 1.024858 | H | 7.240334 | -0.06076 | 0.834147 |
| C | -2.54711 | 0.530453 | 1.09686 | H | -7.33898 | -2.26515 | -0.51726 |
| C | -3.31149 | -0.48419 | 0.501434 | H | 7.776409 | -3.05251 | 0.578898 |
| C | -2.74038 | -1.6146 | -0.17127 | H | 8.868188 | -1.83214 | 1.219408 |
| C | -3.50656 | -2.63013 | -0.76348 | H | 8.898961 | -0.59448 | -0.86874 |
| C | -2.8481 | -3.69317 | -1.39129 | H | 7.809354 | -1.72799 | -1.65577 |
| C | -1.44428 | -3.78437 | -1.44919 | H | 7.758582 | -3.01651 | 3.096083 |
| C | 4.859944 | -0.17272 | 0.732859 | H | 7.22138 | -1.32942 | 3.079042 |
| C | 5.195637 | -2.31675 | -0.53169 | H | 6.162006 | -2.60614 | 2.469056 |
| C | -4.79319 | -0.3952 | 0.563045 | H | 7.285197 | 1.299133 | -1.3552 |
| C | -4.98307 | -2.55307 | -0.70823 | H | 7.847498 | 0.498877 | -2.83117 |
| N | -5.53597 | -1.43209 | -0.03952 | H | 6.231748 | 0.164782 | -2.20812 |
| N | 5.673331 | -1.17836 | 0.14859 | H | -7.31435 | -0.54325 | 1.999665 |
| O | 5.357828 | 0.78596 | 1.310805 | H | -8.66258 | -1.48564 | 1.377997 |
| O | 5.962687 | -3.14342 | -1.0105 | H | -7.34695 | 0.772235 | -0.24555 |
| O | -5.35543 | 0.542178 | 1.118315 | H | -6.99699 | -0.10355 | -1.73007 |
| O | -5.69411 | -3.41268 | -1.2124 | H | -7.56543 | -2.74501 | 3.200881 |
| N | -0.01344 | 1.148417 | 1.475091 | H | -7.32551 | -3.61359 | 1.677084 |
| C | 7.154834 | -0.99566 | 0.278739 | H | -6.00641 | -2.6699 | 2.379206 |
| C | -7.03197 | -1.36096 | 0.010972 | H | -9.3622 | 0.583926 | -1.74941 |
| C | 7.809568 | -2.1051 | 1.12235 | H | -9.29133 | -1.17918 | -1.63977 |
| C | 7.842098 | -0.79687 | -1.08482 | H | -9.67333 | -0.2118 | -0.20829 |
| C | 7.203057 | -2.27065 | 2.518943 | H | -0.94449 | 2.386096 | 2.846762 |
| C | 7.268584 | 0.356653 | -1.91321 | H | 0.805334 | 2.422564 | 2.885099 |
| C | -7.56933 | -1.45332 | 1.451114 | H | -0.11151 | 4.939119 | 3.10932 |
| C | -7.55818 | -0.15207 | -0.78872 | H | -0.12795 | 7.046103 | 1.838476 |
| C | -7.08897 | -2.69027 | 2.216988 | H | -0.0159 | 2.685816 | -0.5528 |
| C | -9.0543 | -0.24938 | -1.11003 | H | -0.11435 | 8.243373 | -0.34844 |
| C | -0.05514 | 2.405058 | 2.206169 | H | -0.07423 | 8.168768 | -2.81969 |
| C | -0.06243 | 3.663482 | 1.350648 | H | -0.018 | 5.981326 | -4.00603 |
| C | -0.09436 | 4.91697 | 2.022119 | H | -0.00187 | 3.868732 | -2.72465 |
| C | -0.10358 | 6.094129 | 1.314862 |  |  |  |  |

Table S7. Cartesian coordinates for optimized structure for [**PDI**–**MeNP**]**^•−^**

Symbolic Z-matrix:

Charge = -1 Multiplicity = 2

| Atom | X | Y | Z | Atom | X | Y | Z |
| --- | --- | --- | --- | --- | --- | --- | --- |
| C | 2.551729 | 0.69099 | 1.136785 | C | -0.13947 | 6.171369 | -0.0775 |
| C | 1.171987 | 0.507946 | 1.014131 | C | -0.08316 | 4.925085 | -0.78192 |
| C | 0.741628 | -0.65126 | 0.350819 | C | -0.0578 | 3.708953 | -0.04304 |
| C | 1.51326 | -1.66173 | -0.19975 | C | -0.16487 | 7.383053 | -0.81996 |
| C | 2.913563 | -1.48365 | -0.07645 | C | -0.13581 | 7.367511 | -2.19877 |
| C | 3.405271 | -0.3157 | 0.5871 | C | -0.0799 | 6.134776 | -2.89667 |
| C | 0.867753 | -2.78059 | -0.82187 | C | -0.05427 | 4.942863 | -2.20349 |
| C | 1.759032 | -3.75507 | -1.35038 | H | 3.019314 | 1.543831 | 1.616512 |
| C | 3.141424 | -3.59995 | -1.24702 | H | 1.369338 | -4.64097 | -1.84322 |
| C | 3.745856 | -2.4852 | -0.61907 | H | 3.800865 | -4.35839 | -1.65764 |
| C | -0.63081 | -0.6889 | 0.328292 | H | -3.06642 | 1.379195 | 1.511768 |
| C | -1.32743 | -1.73965 | -0.24668 | H | -3.41411 | -4.55229 | -1.78435 |
| C | -0.60141 | -2.82044 | -0.84674 | H | -0.96666 | -4.70261 | -1.88612 |
| C | -1.14546 | 0.444652 | 0.975473 | H | 7.228308 | -0.05263 | 0.80856 |
| C | -2.53669 | 0.552554 | 1.050987 | H | -7.30055 | -2.37032 | -0.4497 |
| C | -3.31436 | -0.49919 | 0.475256 | H | 7.78149 | -3.04569 | 0.620934 |
| C | -2.7387 | -1.63839 | -0.16995 | H | 8.863097 | -1.80115 | 1.248153 |
| C | -3.49716 | -2.68233 | -0.74123 | H | 8.90301 | -0.61459 | -0.86546 |
| C | -2.81152 | -3.76051 | -1.35013 | H | 7.82403 | -1.78033 | -1.63278 |
| C | -1.42019 | -3.84042 | -1.40601 | H | 7.738892 | -2.97275 | 3.133714 |
| C | 4.854501 | -0.15982 | 0.704279 | H | 7.201176 | -1.282 | 3.087518 |
| C | 5.193817 | -2.34504 | -0.51541 | H | 6.147234 | -2.56535 | 2.48275 |
| C | -4.77457 | -0.42132 | 0.538016 | H | 7.285379 | 1.25276 | -1.426 |
| C | -4.952 | -2.62226 | -0.68771 | H | 7.854237 | 0.404839 | -2.87754 |
| N | -5.51173 | -1.48968 | -0.03043 | H | 6.237405 | 0.085492 | -2.23985 |
| N | 5.670693 | -1.18607 | 0.1427 | H | -7.32199 | -0.49364 | 1.954038 |
| O | 5.377359 | 0.813803 | 1.265086 | H | -8.63915 | -1.52434 | 1.391228 |
| O | 5.990706 | -3.17933 | -0.96839 | H | -7.33914 | 0.676846 | -0.36102 |
| O | -5.37261 | 0.528308 | 1.064688 | H | -6.98906 | -0.2883 | -1.79086 |
| O | -5.685 | -3.49369 | -1.17632 | H | -7.50252 | -2.61272 | 3.303473 |
| N | -0.01314 | 1.19325 | 1.403004 | H | -7.22948 | -3.57735 | 1.839667 |
| C | 7.144494 | -1.00089 | 0.274571 | H | -5.94496 | -2.54012 | 2.471107 |
| C | -7.00075 | -1.43316 | 0.023907 | H | -9.36147 | 0.376593 | -1.85176 |
| C | 7.806652 | -2.08689 | 1.145829 | H | -9.27472 | -1.37661 | -1.62133 |
| C | 7.848781 | -0.83346 | -1.08662 | H | -9.66232 | -0.31593 | -0.25691 |
| C | 7.188629 | -2.23234 | 2.541356 | H | -0.94629 | 2.394913 | 2.803793 |
| C | 7.273959 | 0.292352 | -1.95423 | H | 0.808786 | 2.446358 | 2.827953 |
| C | -7.54687 | -1.44549 | 1.465717 | H | -0.16475 | 4.949194 | 3.115196 |
| C | -7.54657 | -0.28113 | -0.84586 | H | -0.21084 | 7.084661 | 1.888394 |
| C | -7.02637 | -2.61097 | 2.315977 | H | -0.01549 | 2.767533 | -0.58248 |
| C | -9.04438 | -0.4096 | -1.15676 | H | -0.20774 | 8.326796 | -0.28122 |
| C | -0.05979 | 2.424719 | 2.156964 | H | -0.15564 | 8.300695 | -2.75491 |
| C | -0.08624 | 3.707555 | 1.334017 | H | -0.05722 | 6.132079 | -3.98279 |
| C | -0.14204 | 4.949563 | 2.02728 | H | -0.01142 | 3.996743 | -2.73652 |
| C | -0.16793 | 6.143803 | 1.344805 |  |  |  |  |

Table S8. Cartesian coordinates for optimized structure for **PDI**–**MePY**

Symbolic Z-matrix:

Charge = 0 Multiplicity = 1

| Atom | X | Y | Z | Atom | X | Y | Z |
| --- | --- | --- | --- | --- | --- | --- | --- |
| C | -2.17761 | -0.38214 | -1.1783 | C | 0.520945 | 4.160841 | 1.205548 |
| C | -0.76563 | -0.30908 | -1.07659 | C | 0.394333 | 3.393482 | 0.087503 |
| C | -0.15123 | -1.33165 | -0.35533 | C | -3.81223 | 6.295946 | 0.257436 |
| C | -0.73332 | -2.41354 | 0.282898 | C | -2.65968 | 6.175951 | 1.107191 |
| C | -2.13566 | -2.48046 | 0.1793 | C | -2.50459 | 6.949199 | 2.268177 |
| C | -2.82444 | -1.45911 | -0.55419 | C | -1.37441 | 6.812682 | 3.072745 |
| C | 0.087656 | -3.36257 | 0.973649 | C | -0.37507 | 5.902763 | 2.737249 |
| C | -0.61521 | -4.41516 | 1.579654 | C | -8.57888 | -2.21305 | 1.176333 |
| C | -2.01744 | -4.5004 | 1.493628 | C | 9.615382 | -0.69228 | -1.75549 |
| C | -2.78534 | -3.55515 | 0.804978 | H | -2.78747 | 0.344199 | -1.70092 |
| C | 1.209759 | -1.13611 | -0.35451 | H | -0.08448 | -5.18716 | 2.128337 |
| C | 2.07751 | -2.00323 | 0.285142 | H | -2.5392 | -5.32276 | 1.971447 |
| C | 1.562679 | -3.14673 | 0.9765 | H | 3.263042 | 1.220357 | -1.70583 |
| C | 1.507635 | 0.017075 | -1.07651 | H | 4.640531 | -4.26368 | 1.985703 |
| C | 2.879161 | 0.355678 | -1.17499 | H | 2.251399 | -4.84057 | 2.138033 |
| C | 3.808931 | -0.48641 | -0.54576 | H | -6.6677 | -1.85474 | -0.71912 |
| C | 3.440011 | -1.66266 | 0.184359 | H | 8.064075 | -1.76397 | 0.10974 |
| C | 4.370608 | -2.50246 | 0.813692 | H | -6.69188 | -4.87303 | -0.28209 |
| C | 3.905706 | -3.62697 | 1.504705 | H | -7.97707 | -3.89761 | -0.98145 |
| C | 2.537914 | -3.94975 | 1.587574 | H | -6.92935 | -3.43592 | 1.875641 |
| C | -4.30177 | -1.56012 | -0.65431 | H | -6.5707 | -1.71686 | 1.775818 |
| C | -4.26011 | -3.6597 | 0.724373 | H | -6.69976 | -4.98163 | -2.80002 |
| C | 5.252903 | -0.14371 | -0.62628 | H | -6.44781 | -3.23117 | -2.88486 |
| C | 5.811179 | -2.18182 | 0.725845 | H | -5.18859 | -4.27926 | -2.22179 |
| N | 6.169626 | -1.03743 | -0.03322 | H | 7.564023 | -1.12252 | -2.2603 |
| N | -4.92821 | -2.6567 | -0.00607 | H | 7.845873 | 0.555889 | -1.85135 |
| O | -4.96141 | -0.73352 | -1.27295 | H | 7.807095 | -0.23994 | 1.925738 |
| O | -4.87318 | -4.57321 | 1.263871 | H | 9.24852 | 0.057887 | 0.989435 |
| O | 5.631503 | 0.877317 | -1.18864 | H | 8.273974 | 2.196281 | 1.694393 |
| O | 6.661744 | -2.87135 | 1.274328 | H | 8.154736 | 2.093036 | -0.06624 |
| N | 0.273381 | 0.542725 | -1.51926 | H | 6.725774 | 1.822396 | 0.921845 |
| C | -6.42089 | -2.72786 | -0.11323 | H | 1.118712 | 2.179617 | -2.44521 |
| C | 7.647481 | -0.78652 | -0.13893 | H | -0.11384 | 1.375144 | -3.40641 |
| C | -6.88874 | -3.97918 | -0.8791 | H | -2.15931 | 2.302564 | -3.64384 |
| C | -7.08397 | -2.54186 | 1.266619 | H | -3.91985 | 3.917094 | -3.03781 |
| C | -6.27093 | -4.123 | -2.27375 | H | -4.81733 | 5.639636 | -1.50041 |
| C | 8.109312 | -0.46435 | -1.57281 | H | 1.401449 | 4.063342 | 1.834859 |
| C | 8.156558 | 0.162852 | 0.967803 | H | 1.177831 | 2.685555 | -0.15529 |
| C | 7.802674 | 1.650263 | 0.869848 | H | -4.58418 | 7.009608 | 0.532227 |
| C | 0.129923 | 1.714477 | -2.39307 | H | -3.28054 | 7.66129 | 2.535523 |
| C | -0.9142 | 2.718466 | -1.94437 | H | -1.27287 | 7.420305 | 3.966909 |
| C | -2.05065 | 2.895883 | -2.73936 | H | 0.504395 | 5.80016 | 3.36698 |
| C | -3.04537 | 3.808109 | -2.40306 | H | -8.98446 | -2.0042 | 2.171294 |
| C | -2.93199 | 4.589583 | -1.24554 | H | -8.75838 | -1.32961 | 0.552808 |
| C | -1.77425 | 4.438508 | -0.42071 | H | -9.15909 | -3.04095 | 0.757161 |
| C | -0.75517 | 3.496639 | -0.77196 | H | 9.902314 | -0.52694 | -2.79871 |
| C | -3.94138 | 5.539463 | -0.86556 | H | 10.21101 | -0.00768 | -1.14296 |
| C | -1.64055 | 5.238299 | 0.755068 | H | 9.905545 | -1.71504 | -1.48872 |
| C | -0.48743 | 5.106982 | 1.585311 |  |  |  |  |

Table S9. Cartesian coordinates for optimized structure for [**PDI**–**MePY**]**^•−^**

Symbolic Z-matrix:

Charge = -1 Multiplicity = 2

| Atom | X | Y | Z | Atom | X | Y | Z |
| --- | --- | --- | --- | --- | --- | --- | --- |
| C | 2.375171 | -0.20614 | 1.119653 | C | -1.38959 | 4.139639 | -0.97145 |
| C | 0.982234 | -0.25923 | 1.010753 | C | -0.96919 | 3.351539 | 0.058717 |
| C | 0.446965 | -1.34108 | 0.293664 | C | 2.557287 | 7.033584 | -0.39733 |
| C | 1.122426 | -2.37921 | -0.3283 | C | 1.338964 | 6.739029 | -1.10303 |
| C | 2.534177 | -2.32845 | -0.22132 | C | 0.883233 | 7.531718 | -2.17022 |
| C | 3.13168 | -1.24758 | 0.49948 | C | -0.30258 | 7.21957 | -2.83719 |
| C | 0.376294 | -3.40636 | -0.99499 | C | -1.05773 | 6.111546 | -2.45454 |
| C | 1.17399 | -4.41905 | -1.59558 | C | 8.91362 | -1.50897 | -1.16494 |
| C | 2.5661 | -4.38478 | -1.51348 | C | -9.30202 | -1.72006 | 1.833212 |
| C | 3.271051 | -3.36312 | -0.83497 | H | 2.921368 | 0.573122 | 1.636651 |
| C | -0.92416 | -1.27189 | 0.30548 | H | 0.703347 | -5.24059 | -2.12765 |
| C | -1.71458 | -2.23065 | -0.30752 | H | 3.152996 | -5.16978 | -1.98043 |
| C | -1.09106 | -3.3282 | -0.98635 | H | -3.16879 | 0.88048 | 1.699989 |
| C | -1.33246 | -0.14442 | 1.034141 | H | -4.05226 | -4.7789 | -1.94499 |
| C | -2.71014 | 0.05849 | 1.160422 | H | -1.6273 | -5.11178 | -2.12112 |
| C | -3.58212 | -0.89202 | 0.54345 | H | 6.961537 | -1.35278 | 0.721233 |
| C | -3.11161 | -2.02853 | -0.18349 | H | -7.67463 | -2.58401 | -0.08314 |
| C | -3.96324 | -2.97397 | -0.79218 | H | 7.276453 | -4.34577 | 0.226674 |
| C | -3.37945 | -4.06324 | -1.48273 | H | 8.44586 | -3.2603 | 0.980191 |
| C | -2.00053 | -4.24685 | -1.58062 | H | 7.375376 | -2.8569 | -1.89844 |
| C | 4.590104 | -1.22431 | 0.604587 | H | 6.871968 | -1.17522 | -1.76627 |
| C | 4.72725 | -3.34946 | -0.75278 | H | 7.238242 | -4.52119 | 2.738138 |
| C | -5.0296 | -0.69939 | 0.648447 | H | 6.81679 | -2.80201 | 2.860365 |
| C | -5.40587 | -2.80745 | -0.68701 | H | 5.679586 | -3.94511 | 2.135935 |
| N | -5.86436 | -1.68252 | 0.060971 | H | -7.21009 | -1.97146 | 2.298164 |
| N | 5.307637 | -2.28581 | -0.021 | H | -7.65162 | -0.31449 | 1.949 |
| O | 5.202502 | -0.33232 | 1.2094 | H | -7.56765 | -0.96707 | -1.84901 |
| O | 5.445384 | -4.21021 | -1.28105 | H | -9.04793 | -0.90849 | -0.92302 |
| O | -5.52854 | 0.278622 | 1.22301 | H | -8.37162 | 1.370805 | -1.53185 |
| O | -6.21746 | -3.58771 | -1.20529 | H | -8.25315 | 1.212528 | 0.228375 |
| N | -0.13642 | 0.503324 | 1.453259 | H | -6.79082 | 1.178044 | -0.74372 |
| C | 6.793083 | -2.2336 | 0.098531 | H | -1.11522 | 1.913738 | 2.589619 |
| C | -7.35029 | -1.57851 | 0.191466 | H | 0.366051 | 1.309898 | 3.319257 |
| C | 7.373137 | -3.45059 | 0.846699 | H | 2.22725 | 2.578533 | 3.345539 |
| C | 7.451437 | -1.9644 | -1.27105 | H | 3.563461 | 4.529843 | 2.651876 |
| C | 6.740863 | -3.69097 | 2.223156 | H | 3.902736 | 6.484394 | 1.160187 |
| C | -7.82506 | -1.34563 | 1.639333 | H | -2.30907 | 3.897053 | -1.49758 |
| C | -7.97904 | -0.65729 | -0.88071 | H | -1.55837 | 2.483515 | 0.327495 |
| C | -7.83539 | 0.861504 | -0.72103 | H | 3.140037 | 7.896902 | -0.70891 |
| C | -0.08054 | 1.633855 | 2.369262 | H | 1.468872 | 8.39569 | -2.47439 |
| C | 0.696195 | 2.842391 | 1.86445 | H | -0.63837 | 7.843157 | -3.6611 |
| C | 1.88193 | 3.193035 | 2.51798 | H | -1.97924 | 5.870536 | -2.97799 |
| C | 2.638877 | 4.296132 | 2.130815 | H | 9.305054 | -1.23992 | -2.1529 |
| C | 2.224494 | 5.1051 | 1.063592 | H | 9.011996 | -0.62847 | -0.51758 |
| C | 1.012093 | 4.777091 | 0.380392 | H | 9.562465 | -2.29419 | -0.76146 |
| C | 0.244047 | 3.636214 | 0.78075 | H | -9.58895 | -1.61759 | 2.886214 |
| C | 2.978731 | 6.252823 | 0.6364 | H | -9.96826 | -1.07352 | 1.250567 |
| C | 0.571091 | 5.60036 | -0.70267 | H | -9.49974 | -2.7568 | 1.533011 |
| C | -0.64054 | 5.289431 | -1.39239 |  |  |  |  |

Table S10. Calculated (TDDFT) values of the five lowest energy transition of **PDI**–**MeR**

| **PDI**–**MeBZ** | | | |
| --- | --- | --- | --- |
| No. | Calculated  transition energy (nm) | Oscillator  Strength  (f) | Orbital excitation contribution (%) |
| 1 | 525.1787 | 0.8637 | HOMO → LUMO (99%) |
| 2 | 469.9219 | 0.1375 | H-1 → LUMO (97%) |
| 3 | 376.7486 | 0.0045 | H-2 → LUMO (99%) |
| 4 | 365.9294 | 0.0217 | H-5 → LUMO (10%), H-4 → LUMO (82%) |
| 5 | 364.6487 | 0.001 | H-3 → LUMO (94%) |
|  | | | |
| **PDI**–**MeNP** | | | |
| No. | Calculated  transition energy (nm) | Oscillator  Strength  (f) | Orbital excitation contribution (%) |
| 1 | 531.6418 | 0.5988 | H-1 → LUMO (12%), HOMO → LUMO (87%) |
| 2 | 507.3418 | 0.2711 | H-1 → LUMO (88%), HOMO → LUMO (12%) |
| 3 | 463.8565 | 0.1354 | H-2 → LUMO (97%) |
| 4 | 400.5304 | 0.0014 | H-3 → LUMO (100%) |
| 5 | 364.3165 | 0.0227 | H-5 → LUMO (16%), H-4 → LUMO (83%) |
|  | | | |
| **PDI**–**MePY** | | | |
| No. | Calculated  transition energy (nm) | Oscillator  Strength  (f) | Orbital excitation contribution (%) |
| 1 | 619.0852 | 0.0038 | HOMO → LUMO (100%) |
| 2 | 524.823 | 0.842 | H-1 → LUMO (99%) |
| 3 | 467.4768 | 0.1504 | H-2 → LUMO (96%) |
| 4 | 423.8921 | 0.0001 | H-3 → LUMO (100%) |
| 5 | 366.2212 | 0.0028 | HOMO → L+2 (95%) |

Table S11. Calculated (TDDFT) values of the five lowest energy transition of [**PDI**–**MeR**]**^•−^**

| [**PDI**–**MeBZ**]**^•−^** | | | |
| --- | --- | --- | --- |
| No. | Calculated  transition energy (nm) | Oscillator  Strength  (f) | Orbital excitation contribution (%) |
| 1 | 850.5467  (D_0_ → D_1_**)** | 0.0404 | 168α (SOMO(α)) → 169α (LUMO(α)) (66%),  167β (HOMO(β)) → 168β (SUMO(β)) (34%) |
| 2 | 709.2917  (D_0_ → D_2_**)** | 0.0385 | 168α (SOMO(α)) → 170α (L+1(α)) (94%) |
| 3 | 644.2411  (D_0_ → D_3_**)** | 0.904 | 168α (SOMO(α)) → 169α (LUMO(α)) (32%),  167β (HOMO(β)) → 168β (SUMO(β)) (63%) |
| 4 | 514.7135 | 0.0834 | 167β (H-1(β)) → 168β (SUMO(β)) (87%) |
| 5 | 489.8044 | 0.0324 | 168α (SOMO(α)) → 171α (L+2(α)) (81%) |
|  | | | |
| [**PDI**–**MeNP**]**^•−^** | | | |
| No. | Calculated  transition energy (nm) | Oscillator  Strength  (f) | Orbital excitation contribution (%) |
| 1 | 850.7802  (D_0_ → D_1_**)** | 0.0394 | 181α (SOMO(α)) → 182α (LUMO(α)) (65%),  180β (HOMO(β)) → 181β (SUMO(β)) (34%) |
| 2 | 710.7555  (D_0_ → D_2_**)** | 0.0382 | 181α (SOMO(α)) → 183α (L+1(α)) (95%) |
| 3 | 644.509  (D_0_ → D_3_**)** | 0.8977 | 181α (SOMO(α)) → 182α (LUMO(α)) (32%),  180β (HOMO(β)) → 181β (SUMO(β)) (63%) |
| 4 | 572.1203 | 0.0003 | 181α (SOMO(α)) → 184α (L+2(α)) (100%) |
| 5 | 514.1799 | 0.0845 | 179β (H-1(β)) → 181β (SUMO(β)) (87%) |
|  | | | |
| [**PDI**–**MePY**]**^•−^** | | | |
| No. | Calculated  transition energy (nm) | Oscillator  Strength  (f) | Orbital excitation contribution (%) |
| 1 | 851.3059  (D_0_ → D_1_**)** | 0.036 | 200α (SOMO(α)) → 202α (L+1(α)) (65%),  199β (HOMO(β)) → 200β (SUMO(β)) (35%) |
| 2 | 803.1625  (D_0_ → D_2_**)** | 0 | 200α (SOMO(α)) → 201α (LUMO(α)) (100%) |
| 3 | 709.4135  (D_0_ → D_3_**)** | 0.0397 | 200α (SOMO(α)) → 203α (L+2(α)) (95%) |
| 4 | 645.8183  (D_0_ → D_4_**)** | 0.8716 | 200α (SOMO(α)) → 202α (L+1(α)) (33%),  199β (HOMO(β)) → 200β (SUMO(β)) (62%) |
| 5 | 596.0779 | 0 | 198α (H-1(α)) → 201α (LUMO(α)) (43%),  198β (H-1(β)) → 202β (L+1(β)) (46%) |

Table S12. Excited states calculated by TDDFT calculations of **PDI**–**MeBZ**

| **PDI**–**MeBZ (B3LYP/6-31+G(d))** |
| --- |
| Excited state symmetry could not be determined. |
| Excited State 1: Singlet-?Sym 2.3608 eV 525.18 nm f=0.8637 <S**2>=0.000 |
| 157 ->168 -0.01661 |
| 157 ->170 0.02460 |
| 158 ->169 0.02045 |
| 160 ->169 -0.01343 |
| 162 ->168 -0.02518 |
| 162 ->170 0.01958 |
| 162 ->171 -0.02490 |
| 163 ->168 -0.02997 |
| 163 ->170 0.03922 |
| 166 ->168 -0.01869 |
| 166 ->169 -0.02569 |
| 166 ->172 -0.04574 |
| 166 ->181 0.01055 |
| 167 ->168 0.70313 |
| 167 ->170 -0.01445 |
| 167 ->171 0.01049 |
| This state for optimization and/or second-order correction. |
| Total Energy, E(TD-HF/TD-DFT) = -2048.74000507 |
| Copying the excited state density for this state as the 1-particle RhoCI density. |
|  |
| Excited state symmetry could not be determined. |
| Excited State 2: Singlet-?Sym 2.6384 eV 469.93 nm f=0.1375 <S**2>=0.000 |
| 149 ->168 -0.01230 |
| 150 ->168 0.01098 |
| 154 ->168 0.01293 |
| 157 ->169 0.01046 |
| 158 ->168 -0.03790 |
| 158 ->170 -0.01073 |
| 158 ->171 -0.02495 |
| 160 ->171 0.01839 |
| 161 ->171 -0.01337 |
| 162 ->181 -0.01005 |
| 163 ->169 0.03913 |
| 166 ->168 0.69565 |
| 166 ->170 0.01172 |
| 166 ->171 0.01736 |
| 167 ->168 0.01871 |
| 167 ->169 0.04863 |
| 167 ->172 0.08778 |
| 167 ->173 0.01173 |
| 167 ->181 -0.01137 |
|  |
| Excited state symmetry could not be determined. |
| Excited State 3: Singlet-?Sym 3.2909 eV 376.75 nm f=0.0045 <S**2>=0.000 |
| 137 ->168 -0.01277 |
| 158 ->168 -0.01726 |
| 160 ->168 -0.01881 |
| 161 ->168 -0.01372 |
| 162 ->168 0.02898 |
| 163 ->169 0.01202 |
| 165 ->168 0.70303 |
| 165 ->169 -0.01104 |
| 165 ->170 0.02777 |
| 167 ->169 0.02113 |
| 167 ->171 0.01545 |
| 167 ->172 0.03112 |
|  |
| Excited state symmetry could not be determined. |
| Excited State 4: Singlet-?Sym 3.3882 eV 365.93 nm f=0.0217 <S**2>=0.000 |
| 149 ->169 -0.01116 |
| 155 ->168 -0.02493 |
| 156 ->168 -0.01769 |
| 157 ->168 0.04001 |
| 158 ->169 -0.01816 |
| 160 ->168 -0.02829 |
| 161 ->168 -0.02195 |
| 161 ->169 0.04838 |
| 162 ->168 0.22829 |
| 163 ->168 0.64128 |
| 164 ->168 0.16151 |
| 165 ->168 -0.01392 |
| 166 ->172 -0.01160 |
| 167 ->168 0.03601 |
|  |
| Excited state symmetry could not be determined. |
| Excited State 5: Singlet-?Sym 3.4001 eV 364.64 nm f=0.0010 <S**2>=0.000 |
| 161 ->169 -0.01147 |
| 162 ->168 -0.05054 |
| 163 ->168 -0.15173 |
| 164 ->168 0.68710 |
| 164 ->169 -0.01034 |
| 164 ->170 0.02685 |
| 167 ->169 -0.02302 |

Table S13. Excited states calculated by TDDFT calculations of **PDI**–**MeNP**

| **PDI**–**MeNP (B3LYP/6-31+G(d))** |
| --- |
| Excited state symmetry could not be determined. |
| Excited State 1: Singlet-?Sym 2.3321 eV 531.65 nm f=0.5988 <S**2>=0.000 |
| 168 -> 181 0.01517 |
| 168 -> 183 -0.02025 |
| 171 -> 182 0.01658 |
| 174 -> 182 0.01327 |
| 175 -> 181 -0.01803 |
| 175 -> 183 0.01665 |
| 175 -> 185 -0.02104 |
| 176 -> 181 -0.02488 |
| 176 -> 183 0.03340 |
| 177 -> 181 -0.01155 |
| 178 -> 182 0.02066 |
| 178 -> 186 -0.03814 |
| 179 -> 181 -0.24637 |
| 179 -> 183 -0.01321 |
| 180 -> 181 0.65996 |
| This state for optimization and/or second-order correction. |
| Total Energy, E(TD-HF/TD-DFT) = -2202.39812399 |
| Copying the excited state density for this state as the 1-particle RhoCI density. |
|  |
| Excited state symmetry could not be determined. |
| Excited State 2: Singlet-?Sym 2.4438 eV 507.35 nm f=0.2711 <S**2>=0.000 |
| 167 -> 181 0.01412 |
| 168 -> 183 -0.01392 |
| 171 -> 182 0.01051 |
| 175 -> 181 -0.01802 |
| 175 -> 183 0.01192 |
| 175 -> 185 -0.01444 |
| 176 -> 181 -0.01488 |
| 176 -> 183 0.02261 |
| 178 -> 182 0.01463 |
| 178 -> 186 -0.02515 |
| 179 -> 181 0.66164 |
| 179 -> 183 0.01702 |
| 180 -> 181 0.24435 |
| 180 -> 183 -0.01223 |
|  |
| Excited state symmetry could not be determined. |
| Excited State 3: Singlet-?Sym 2.6729 eV 463.85 nm f=0.1354 <S**2>=0.000 |
| 158 -> 181 0.01485 |
| 166 -> 181 0.01327 |
| 166 -> 183 0.01016 |
| 171 -> 181 0.04007 |
| 171 -> 183 0.01051 |
| 171 -> 185 0.02433 |
| 174 -> 185 0.02616 |
| 176 -> 182 -0.04085 |
| 177 -> 184 0.01036 |
| 178 -> 181 0.69497 |
| 178 -> 183 0.01027 |
| 178 -> 185 0.01779 |
| 179 -> 186 0.02051 |
| 180 -> 182 -0.05042 |
| 180 -> 186 0.08941 |
| 180 -> 187 -0.01614 |
|  |
| Excited state symmetry could not be determined. |
| Excited State 4: Singlet-?Sym 3.0955 eV 400.53 nm f=0.0014 <S**2>=0.000 |
| 163 -> 181 0.01545 |
| 175 -> 181 -0.01078 |
| 177 -> 181 0.70620 |
| 177 -> 183 0.02202 |
| 179 -> 181 -0.01322 |
|  |
| Excited state symmetry could not be determined. |
| Excited State 5: Singlet-?Sym 3.4032 eV 364.31 nm f=0.0227 <S**2>=0.000 |
| 158 -> 182 -0.01241 |
| 166 -> 182 -0.01128 |
| 168 -> 181 -0.04762 |
| 169 -> 181 0.01099 |
| 171 -> 182 -0.02510 |
| 174 -> 182 0.03672 |
| 175 -> 181 0.28017 |
| 176 -> 181 0.64307 |
| 178 -> 186 -0.01243 |
| 180 -> 181 0.03558 |

Table S14. Excited states calculated by TDDFT calculations of **PDI**–**MePY**

| **PDI**–**MePY (B3LYP/6-31+G(d))** |
| --- |
| Excited state symmetry could not be determined. |
| Excited State 1: Singlet-?Sym 2.0027 eV 619.09 nm f=0.0038 <S**2>=0.000 |
| 188 -> 200 0.01430 |
| 195 -> 200 -0.01214 |
| 198 -> 200 0.02868 |
| 199 -> 200 0.70559 |
| 199 -> 202 0.01048 |
| 199 -> 203 -0.02117 |
| This state for optimization and/or second-order correction. |
| Total Energy, E(TD-HF/TD-DFT) = -2432.28660581 |
| Copying the excited state density for this state as the 1-particle RhoCI density. |
|  |
| Excited state symmetry could not be determined. |
| Excited State 2: Singlet-?Sym 2.3624 eV 524.82 nm f=0.8420 <S**2>=0.000 |
| 186 -> 200 0.01482 |
| 186 -> 203 0.02188 |
| 187 -> 203 0.01272 |
| 189 -> 202 0.01946 |
| 192 -> 202 0.01375 |
| 193 -> 200 -0.02518 |
| 193 -> 203 -0.02083 |
| 193 -> 205 -0.02474 |
| 194 -> 200 -0.02858 |
| 194 -> 203 -0.03853 |
| 197 -> 202 -0.02532 |
| 197 -> 206 -0.04571 |
| 198 -> 200 0.70287 |
| 198 -> 203 0.01362 |
| 199 -> 200 -0.02836 |
|  |
| Excited state symmetry could not be determined. |
| Excited State 3: Singlet-?Sym 2.6522 eV 467.48 nm f=0.1504 <S**2>=0.000 |
| 177 -> 200 -0.01473 |
| 183 -> 200 0.01250 |
| 189 -> 200 -0.03715 |
| 189 -> 203 0.01043 |
| 189 -> 205 -0.02397 |
| 192 -> 205 -0.02161 |
| 194 -> 202 0.03895 |
| 196 -> 200 0.01806 |
| 197 -> 200 0.69441 |
| 197 -> 203 -0.01092 |
| 197 -> 205 0.01711 |
| 198 -> 202 0.04966 |
| 198 -> 206 0.08905 |
| 199 -> 201 -0.03510 |
| 199 -> 206 -0.01148 |
|  |
| Excited state symmetry could not be determined. |
| Excited State 4: Singlet-?Sym 2.9249 eV 423.89 nm f=0.0001 <S**2>=0.000 |
| 184 -> 200 0.02318 |
| 195 -> 200 -0.01005 |
| 196 -> 200 0.70570 |
| 196 -> 202 0.01364 |
| 196 -> 203 -0.02097 |
| 197 -> 200 -0.01999 |
|  |
| Excited state symmetry could not be determined. |
| Excited State 5: Singlet-?Sym 3.3855 eV 366.22 nm f=0.0028 <S**2>=0.000 |
| 188 -> 202 0.01321 |
| 189 -> 200 0.02041 |
| 192 -> 200 0.02450 |
| 193 -> 200 -0.04699 |
| 194 -> 200 -0.11357 |
| 195 -> 200 0.04206 |
| 195 -> 202 -0.01059 |
| 197 -> 200 0.01080 |
| 197 -> 202 -0.01545 |
| 198 -> 200 -0.01025 |
| 198 -> 202 -0.05822 |
| 198 -> 203 0.01110 |
| 199 -> 201 -0.03809 |
| 199 -> 202 0.68791 |
| 199 -> 203 -0.02048 |
| 199 -> 206 -0.02896 |
| 199 -> 208 0.02025 |

Table S15. Excited states calculated by TDDFT calculations of [**PDI**–**MeBZ**]**^•−^**

| [**PDI**–**MeBZ**]**^•−^ (B3LYP/6-31+G(d))** |
| --- |
| Excited state symmetry could not be determined. |
| Excited State 1: 2.006-?Sym 1.4577 eV 850.57 nm f=0.0404 <S**2>=0.756 |
| 155A ->170A -0.02199 |
| 158A ->169A -0.01153 |
| 158A ->175A 0.01213 |
| 166A ->175A -0.01064 |
| 167A ->171A 0.02109 |
| 167A ->178A -0.01010 |
| 167A ->179A -0.01203 |
| 168A ->169A 0.81041 |
| 168A ->187A 0.01444 |
| 157B ->168B 0.01059 |
| 157B ->170B 0.02819 |
| 158B ->176B -0.01474 |
| 162B ->168B -0.01812 |
| 163B ->169B 0.01228 |
| 165B ->170B -0.02283 |
| 166B ->174B -0.01679 |
| 167B ->168B 0.58370 |
| 167B ->172B 0.01028 |
| This state for optimization and/or second-order correction. |
| Total Energy, E(TD-HF/TD-DFT) = -2048.90824140 |
| Copying the excited state density for this state as the 1-particle RhoCI density. |
|  |
| Excited state symmetry could not be determined. |
| Excited State 2: 2.020-?Sym 1.7480 eV 709.29 nm f=0.0385 <S**2>=0.770 |
| 164A ->169A 0.01721 |
| 165A ->169A -0.02012 |
| 167A ->174A 0.03340 |
| 167A ->175A -0.04640 |
| 168A ->169A 0.05111 |
| 168A ->170A 0.97201 |
| 168A ->171A 0.02853 |
| 168A ->205A 0.01008 |
| 153B ->168B -0.01710 |
| 154B ->168B -0.01138 |
| 155B ->169B 0.01213 |
| 156B ->168B 0.01150 |
| 156B ->169B -0.01156 |
| 157B ->169B 0.02987 |
| 158B ->168B 0.05330 |
| 158B ->181B -0.01364 |
| 162B ->169B -0.01224 |
| 162B ->189B 0.01471 |
| 163B ->168B -0.01322 |
| 163B ->172B 0.02199 |
| 164B ->169B 0.01934 |
| 165B ->169B 0.01636 |
| 166B ->168B 0.18329 |
| 166B ->181B 0.01079 |
| 167B ->168B -0.06310 |
| 167B ->174B 0.03756 |
| 167B ->176B -0.03699 |
|  |
| Excited state symmetry could not be determined. |
| Excited State 3: 2.061-?Sym 1.9245 eV 644.25 nm f=0.9040 <S**2>=0.812 |
| 151A ->175A 0.01490 |
| 153A ->175A 0.01487 |
| 154A ->174A 0.02001 |
| 155A ->170A -0.03929 |
| 155A ->171A 0.01808 |
| 157A ->170A -0.01622 |
| 158A ->169A -0.03259 |
| 158A ->174A 0.01029 |
| 158A ->187A -0.01847 |
| 159A ->169A -0.02749 |
| 159A ->187A -0.01712 |
| 160A ->169A -0.01251 |
| 160A ->170A 0.02803 |
| 162A ->170A -0.01978 |
| 163A ->170A 0.01086 |
| 164A ->170A 0.07504 |
| 165A ->170A -0.07109 |
| 165A ->171A 0.01084 |
| 165A ->205A -0.01180 |
| 166A ->174A -0.01146 |
| 166A ->175A 0.01251 |
| 167A ->170A -0.01974 |
| 167A ->171A -0.02807 |
| 167A ->178A 0.02373 |
| 167A ->179A 0.03036 |
| 168A ->169A -0.56598 |
| 168A ->170A 0.08011 |
| 168A ->172A 0.02778 |
| 168A ->174A -0.05653 |
| 168A ->175A -0.02127 |
| 168A ->187A -0.01142 |
| 133B ->189B -0.01002 |
| 152B ->181B -0.01075 |
| 153B ->176B 0.01722 |
| 154B ->174B -0.01966 |
| 154B ->189B -0.01135 |
| 157B ->172B 0.02970 |
| 158B ->169B -0.01199 |
| 159B ->172B 0.01181 |
| 161B ->169B 0.01204 |
| 161B ->172B -0.01286 |
| 162B ->168B 0.02735 |
| 162B ->169B 0.01520 |
| 162B ->172B -0.03865 |
| 163B ->169B 0.08734 |
| 163B ->174B -0.01584 |
| 163B ->189B -0.02205 |
| 164B ->168B -0.02524 |
| 164B ->172B 0.03162 |
| 166B ->168B 0.01407 |
| 166B ->169B 0.04146 |
| 166B ->171B 0.01659 |
| 166B ->174B 0.08752 |
| 166B ->189B -0.01448 |
| 167B ->168B 0.79089 |
| 167B ->172B -0.03471 |
| 167B ->181B 0.02096 |
|  |
| Excited state symmetry could not be determined. |
| Excited State 4: 2.030-?Sym 2.4088 eV 514.70 nm f=0.0834 <S**2>=0.780 |
| 149A ->169A -0.01014 |
| 154A ->171A 0.01507 |
| 154A ->179A 0.01128 |
| 155A ->174A 0.01021 |
| 155A ->187A -0.01254 |
| 158A ->170A 0.01708 |
| 158A ->171A 0.01504 |
| 159A ->169A 0.01174 |
| 159A ->171A 0.01306 |
| 160A ->169A -0.02553 |
| 162A ->169A 0.02318 |
| 164A ->169A -0.03643 |
| 165A ->169A 0.02039 |
| 166A ->178A 0.02409 |
| 166A ->179A 0.03272 |
| 167A ->169A -0.03565 |
| 167A ->172A 0.01529 |
| 167A ->174A -0.07751 |
| 168A ->170A -0.16022 |
| 168A ->171A -0.27072 |
| 168A ->172A 0.03323 |
| 168A ->173A -0.01155 |
| 168A ->178A -0.01360 |
| 168A ->179A -0.02094 |
| 168A ->201A -0.01099 |
| 168A ->205A 0.01889 |
| 154B ->170B 0.02556 |
| 154B ->172B 0.01010 |
| 158B ->168B -0.02598 |
| 162B ->169B -0.02043 |
| 162B ->174B 0.01156 |
| 163B ->168B 0.03285 |
| 163B ->172B -0.02603 |
| 165B ->169B -0.02900 |
| 166B ->168B 0.93422 |
| 166B ->170B 0.02335 |
| 166B ->172B -0.02521 |
| 166B ->181B 0.02015 |
| 167B ->171B -0.01155 |
| 167B ->174B -0.04988 |
| 167B ->189B 0.01158 |
|  |
| Excited state symmetry could not be determined. |
| Excited State 5: 2.174-?Sym 2.5313 eV 489.80 nm f=0.0324 <S**2>=0.931 |
| 149A ->174A 0.01362 |
| 150A ->174A -0.01149 |
| 155A ->169A -0.01924 |
| 155A ->175A 0.01294 |
| 158A ->170A 0.01745 |
| 158A ->179A 0.01061 |
| 159A ->169A 0.01628 |
| 159A ->170A 0.03440 |
| 159A ->171A 0.01342 |
| 160A ->169A -0.02753 |
| 160A ->170A 0.01618 |
| 162A ->169A 0.02860 |
| 164A ->169A -0.03128 |
| 166A ->171A 0.04443 |
| 167A ->169A 0.12917 |
| 167A ->174A 0.03902 |
| 167A ->175A 0.03658 |
| 167A ->187A -0.01550 |
| 168A ->170A -0.07271 |
| 168A ->171A 0.89889 |
| 168A ->172A -0.09673 |
| 168A ->173A 0.03651 |
| 168A ->174A -0.01107 |
| 168A ->178A 0.02214 |
| 168A ->179A 0.03491 |
| 150B ->174B -0.01922 |
| 151B ->174B -0.01339 |
| 152B ->174B 0.02092 |
| 153B ->168B 0.01673 |
| 153B ->170B 0.02394 |
| 154B ->168B 0.01122 |
| 154B ->172B -0.02693 |
| 157B ->169B 0.01742 |
| 157B ->176B 0.02000 |
| 159B ->169B 0.01071 |
| 161B ->168B -0.01820 |
| 161B ->169B -0.01270 |
| 162B ->168B -0.02210 |
| 162B ->169B -0.03305 |
| 163B ->168B -0.11599 |
| 163B ->170B -0.02104 |
| 163B ->172B -0.01657 |
| 164B ->169B 0.02283 |
| 165B ->169B -0.02344 |
| 165B ->176B -0.01431 |
| 166B ->168B 0.25838 |
| 166B ->170B 0.01517 |
| 166B ->172B 0.02648 |
| 166B ->181B -0.01785 |
| 167B ->169B -0.21738 |
| 167B ->174B -0.06843 |
| 167B ->176B 0.02195 |
| 167B ->189B -0.01233 |

Table S16. Excited states calculated by TDDFT calculations of [**PDI**–**MeNP**]**^•−^**

| [**PDI**–**MeNP**]**^•−^ (B3LYP/6-31+G(d))** |
| --- |
| Excited state symmetry could not be determined. |
| Excited State 1: 2.006-?Sym 1.4573 eV 850.76 nm f=0.0394 <S**2>=0.756 |
| 168A -> 183A -0.02301 |
| 171A -> 182A -0.01068 |
| 171A -> 188A 0.01534 |
| 178A -> 188A -0.01067 |
| 180A -> 186A 0.02117 |
| 180A -> 193A -0.01018 |
| 181A -> 182A 0.80898 |
| 181A -> 201A 0.01538 |
| 168B -> 184B 0.02917 |
| 169B -> 184B -0.01150 |
| 171B -> 189B -0.01359 |
| 174B -> 181B 0.02316 |
| 175B -> 182B 0.01271 |
| 176B -> 184B -0.02314 |
| 179B -> 187B 0.01684 |
| 180B -> 181B 0.58565 |
| 180B -> 186B -0.01033 |
| This state for optimization and/or second-order correction. |
| Total Energy, E(TD-HF/TD-DFT) = -2202.56585418 |
| Copying the excited state density for this state as the 1-particle RhoCI density. |
|  |
| Excited state symmetry could not be determined. |
| Excited State 2: 2.020-?Sym 1.7444 eV 710.75 nm f=0.0382 <S**2>=0.770 |
| 168A -> 182A -0.01044 |
| 176A -> 182A 0.02709 |
| 180A -> 187A 0.03316 |
| 180A -> 188A -0.04630 |
| 181A -> 182A -0.03517 |
| 181A -> 183A 0.97419 |
| 181A -> 186A 0.02894 |
| 165B -> 181B 0.01683 |
| 166B -> 181B -0.01133 |
| 168B -> 182B 0.03239 |
| 169B -> 182B -0.01329 |
| 171B -> 181B 0.05485 |
| 171B -> 196B 0.01104 |
| 174B -> 182B 0.01655 |
| 174B -> 204B 0.01670 |
| 175B -> 181B -0.01545 |
| 175B -> 186B -0.02186 |
| 176B -> 182B 0.02171 |
| 179B -> 181B 0.18141 |
| 179B -> 196B -0.01035 |
| 180B -> 181B 0.04299 |
| 180B -> 187B -0.03848 |
| 180B -> 188B 0.01768 |
| 180B -> 189B -0.03246 |
|  |
| Excited state symmetry could not be determined. |
| Excited State 3: 2.061-?Sym 1.9237 eV 644.51 nm f=0.8977 <S**2>=0.812 |
| 159A -> 188A -0.01295 |
| 165A -> 188A -0.01595 |
| 166A -> 187A 0.02007 |
| 168A -> 183A -0.04170 |
| 168A -> 186A 0.01852 |
| 171A -> 182A -0.02833 |
| 171A -> 187A 0.01042 |
| 171A -> 201A -0.01676 |
| 172A -> 182A -0.03529 |
| 172A -> 201A -0.02207 |
| 173A -> 183A 0.02970 |
| 173A -> 186A 0.01057 |
| 176A -> 183A 0.10600 |
| 178A -> 187A -0.01123 |
| 178A -> 188A 0.01259 |
| 180A -> 183A -0.01837 |
| 180A -> 186A -0.02849 |
| 180A -> 191A -0.02171 |
| 180A -> 192A -0.01186 |
| 180A -> 193A 0.02596 |
| 180A -> 194A -0.01470 |
| 181A -> 182A -0.56930 |
| 181A -> 183A -0.05513 |
| 181A -> 184A -0.01077 |
| 181A -> 185A 0.01847 |
| 181A -> 187A -0.05860 |
| 181A -> 188A -0.02236 |
| 181A -> 201A -0.01173 |
| 164B -> 186B -0.01376 |
| 164B -> 196B 0.01236 |
| 165B -> 189B -0.01523 |
| 166B -> 187B 0.01963 |
| 168B -> 186B -0.02800 |
| 169B -> 186B 0.01158 |
| 174B -> 181B -0.03548 |
| 174B -> 182B 0.02512 |
| 174B -> 186B -0.05224 |
| 175B -> 181B 0.01091 |
| 175B -> 182B 0.08649 |
| 175B -> 186B 0.01497 |
| 175B -> 187B 0.01684 |
| 175B -> 204B 0.01997 |
| 176B -> 181B -0.01319 |
| 177B -> 186B 0.01153 |
| 179B -> 182B 0.04119 |
| 179B -> 187B -0.08884 |
| 179B -> 204B 0.01285 |
| 180B -> 181B 0.79079 |
| 180B -> 186B 0.03539 |
| 180B -> 195B -0.01150 |
| 180B -> 196B -0.01831 |
|  |
| Excited state symmetry could not be determined. |
| Excited State 4: 2.012-?Sym 2.1671 eV 572.13 nm f=0.0003 <S**2>=0.762 |
| 171A -> 184A 0.01372 |
| 178A -> 184A -0.02909 |
| 181A -> 184A 0.99881 |
| 181A -> 185A 0.01089 |
| 181A -> 190A 0.02162 |
| 181A -> 192A -0.01236 |
|  |
| Excited state symmetry could not be determined. |
| Excited State 5: 2.030-?Sym 2.4113 eV 514.19 nm f=0.0845 <S**2>=0.780 |
| 163A -> 182A -0.01232 |
| 166A -> 186A 0.01494 |
| 168A -> 187A 0.01039 |
| 168A -> 201A -0.01327 |
| 171A -> 183A 0.01589 |
| 171A -> 186A 0.01329 |
| 172A -> 183A 0.01396 |
| 172A -> 186A 0.01676 |
| 173A -> 182A -0.03720 |
| 173A -> 187A 0.01247 |
| 173A -> 201A -0.01660 |
| 176A -> 182A -0.04125 |
| 178A -> 191A -0.02220 |
| 178A -> 192A -0.01207 |
| 178A -> 193A 0.02743 |
| 178A -> 194A -0.01596 |
| 180A -> 182A -0.03679 |
| 180A -> 187A -0.07856 |
| 181A -> 183A -0.15810 |
| 181A -> 186A -0.27282 |
| 181A -> 191A 0.01135 |
| 181A -> 193A -0.01728 |
| 181A -> 194A 0.01438 |
| 181A -> 219A -0.01153 |
| 181A -> 220A 0.01213 |
| 166B -> 184B 0.02567 |
| 166B -> 186B -0.01014 |
| 171B -> 181B -0.02556 |
| 174B -> 182B 0.02793 |
| 174B -> 187B 0.01589 |
| 175B -> 181B 0.03238 |
| 175B -> 186B 0.02570 |
| 176B -> 182B -0.02384 |
| 179B -> 181B 0.93455 |
| 179B -> 184B 0.02301 |
| 179B -> 186B 0.02589 |
| 179B -> 195B -0.01134 |
| 179B -> 196B -0.01766 |
| 180B -> 187B 0.05074 |
| 180B -> 204B -0.01039 |

Table S17. Excited states calculated by TDDFT calculations of [**PDI**–**MePY**]**^•−^**

| [**PDI**–**MePY**]**^•−^ (B3LYP/6-31+G(d))** |
| --- |
| Excited state symmetry could not be determined. |
| Excited State 1: 2.006-?Sym 1.4564 eV 851.33 nm f=0.0360 <S**2>=0.756 |
| 186A -> 203A 0.02288 |
| 189A -> 202A 0.01043 |
| 189A -> 208A 0.01264 |
| 197A -> 208A 0.01051 |
| 199A -> 205A 0.01484 |
| 199A -> 206A 0.01397 |
| 200A -> 202A 0.80640 |
| 188B -> 203B -0.03046 |
| 190B -> 210B 0.01249 |
| 193B -> 200B 0.02132 |
| 194B -> 200B -0.01074 |
| 194B -> 202B -0.01150 |
| 195B -> 203B 0.02276 |
| 197B -> 207B -0.01615 |
| 198B -> 200B 0.02085 |
| 199B -> 200B 0.58883 |
| 199B -> 206B -0.01017 |
| This state for optimization and/or second-order correction. |
| Total Energy, E(TD-HF/TD-DFT) = -2432.44147006 |
| Copying the excited state density for this state as the 1-particle RhoCI density. |
|  |
| Excited state symmetry could not be determined. |
| Excited State 2: 2.013-?Sym 1.5437 eV 803.18 nm f=0.0000 <S**2>=0.763 |
| 189A -> 201A -0.01234 |
| 197A -> 201A -0.02877 |
| 198A -> 201A -0.01068 |
| 200A -> 201A 0.99840 |
| 200A -> 202A -0.01484 |
| 200A -> 203A 0.01539 |
| 200A -> 205A 0.01011 |
| 200A -> 206A -0.01764 |
| 200A -> 209A 0.02324 |
| 200A -> 227A 0.01069 |
|  |
| Excited state symmetry could not be determined. |
| Excited State 3: 2.020-?Sym 1.7477 eV 709.41 nm f=0.0397 <S**2>=0.770 |
| 186A -> 202A 0.01024 |
| 195A -> 202A -0.02658 |
| 198A -> 208A -0.01153 |
| 199A -> 207A -0.03200 |
| 199A -> 208A -0.04477 |
| 200A -> 201A -0.01740 |
| 200A -> 202A -0.04131 |
| 200A -> 203A 0.97284 |
| 200A -> 205A -0.02259 |
| 200A -> 206A -0.01750 |
| 200A -> 242A 0.01087 |
| 182B -> 200B 0.01684 |
| 184B -> 200B 0.01126 |
| 188B -> 202B -0.03322 |
| 190B -> 200B 0.05433 |
| 190B -> 216B -0.01364 |
| 193B -> 202B -0.01788 |
| 193B -> 206B -0.01287 |
| 193B -> 225B -0.01332 |
| 193B -> 226B -0.01022 |
| 194B -> 206B -0.01917 |
| 195B -> 202B -0.02121 |
| 197B -> 200B -0.17957 |
| 197B -> 216B -0.01184 |
| 198B -> 200B -0.04457 |
| 199B -> 200B 0.04788 |
| 199B -> 207B -0.03796 |
| 199B -> 209B -0.02225 |
| 199B -> 210B 0.02992 |
|  |
| Excited state symmetry could not be determined. |
| Excited State 4: 2.060-?Sym 1.9198 eV 645.82 nm f=0.8716 <S**2>=0.811 |
| 182A -> 208A -0.01638 |
| 184A -> 207A 0.01919 |
| 186A -> 203A 0.04028 |
| 186A -> 205A 0.01385 |
| 186A -> 206A 0.01190 |
| 187A -> 203A 0.01342 |
| 189A -> 202A 0.02863 |
| 189A -> 207A -0.01015 |
| 189A -> 221A 0.01109 |
| 189A -> 222A -0.01149 |
| 190A -> 202A 0.01242 |
| 191A -> 202A 0.03127 |
| 191A -> 221A 0.01262 |
| 191A -> 222A -0.01360 |
| 192A -> 203A 0.02999 |
| 193A -> 203A 0.01723 |
| 195A -> 203A -0.10345 |
| 195A -> 242A -0.01314 |
| 197A -> 207A -0.01087 |
| 197A -> 208A -0.01263 |
| 199A -> 203A 0.01898 |
| 199A -> 205A -0.02003 |
| 199A -> 206A -0.01890 |
| 199A -> 212A 0.02271 |
| 199A -> 213A 0.02375 |
| 199A -> 215A -0.01368 |
| 200A -> 201A -0.01385 |
| 200A -> 202A -0.57222 |
| 200A -> 203A -0.06261 |
| 200A -> 204A -0.01097 |
| 200A -> 207A -0.05800 |
| 200A -> 208A 0.02263 |
| 181B -> 206B 0.01312 |
| 181B -> 216B 0.01500 |
| 182B -> 209B -0.01004 |
| 182B -> 210B 0.01383 |
| 184B -> 207B -0.01915 |
| 188B -> 206B -0.02898 |
| 190B -> 202B 0.01348 |
| 192B -> 202B 0.01369 |
| 193B -> 200B -0.03253 |
| 193B -> 202B -0.04100 |
| 193B -> 206B -0.04894 |
| 193B -> 216B 0.01099 |
| 194B -> 200B 0.01988 |
| 194B -> 202B -0.07811 |
| 194B -> 206B 0.02453 |
| 194B -> 207B 0.01458 |
| 194B -> 225B -0.01370 |
| 194B -> 226B -0.01200 |
| 195B -> 200B -0.01126 |
| 197B -> 202B 0.03991 |
| 197B -> 207B 0.08719 |
| 197B -> 225B 0.01097 |
| 198B -> 200B 0.01604 |
| 198B -> 207B 0.01684 |
| 199B -> 200B 0.78806 |
| 199B -> 206B 0.03469 |
| 199B -> 216B 0.02220 |
|  |
| Excited state symmetry could not be determined. |
| Excited State 5: 3.470-?Sym 2.0800 eV 596.08 nm f=0.0000 <S**2>=2.761 |
| 169A -> 239A 0.01188 |
| 169A -> 283A -0.01126 |
| 170A -> 239A -0.01437 |
| 170A -> 277A -0.01104 |
| 170A -> 283A 0.01358 |
| 170A -> 285A -0.01085 |
| 183A -> 206A 0.01044 |
| 183A -> 223A -0.01057 |
| 183A -> 227A -0.05779 |
| 183A -> 228A -0.01920 |
| 183A -> 229A 0.01034 |
| 183A -> 231A 0.01004 |
| 184A -> 209A 0.01124 |
| 184A -> 227A -0.01397 |
| 185A -> 209A 0.09638 |
| 185A -> 210A -0.02137 |
| 185A -> 242A 0.01008 |
| 185A -> 243A -0.01375 |
| 185A -> 248A -0.01022 |
| 189A -> 205A -0.02585 |
| 189A -> 206A 0.02955 |
| 190A -> 205A 0.06672 |
| 190A -> 206A -0.07654 |
| 190A -> 227A 0.01815 |
| 196A -> 204A 0.12271 |
| 197A -> 201A 0.05045 |
| 198A -> 201A -0.65713 |
| 199A -> 201A 0.19402 |
| 200A -> 201A -0.01023 |
| 169B -> 239B -0.01375 |
| 169B -> 277B -0.01045 |
| 169B -> 282B 0.01028 |
| 169B -> 283B -0.01093 |
| 170B -> 239B 0.01189 |
| 183B -> 205B 0.01298 |
| 183B -> 222B 0.01185 |
| 183B -> 225B -0.01382 |
| 183B -> 227B 0.05757 |
| 183B -> 228B 0.01954 |
| 183B -> 229B -0.01220 |
| 183B -> 231B -0.01022 |
| 184B -> 208B -0.01461 |
| 185B -> 208B -0.09680 |
| 185B -> 209B 0.01181 |
| 185B -> 210B 0.01556 |
| 185B -> 242B -0.01496 |
| 189B -> 205B 0.10743 |
| 189B -> 206B -0.01353 |
| 189B -> 227B 0.01855 |
| 196B -> 204B -0.12276 |
| 197B -> 201B -0.09530 |
| 198B -> 201B 0.67761 |
| 199B -> 201B -0.06565 |

**
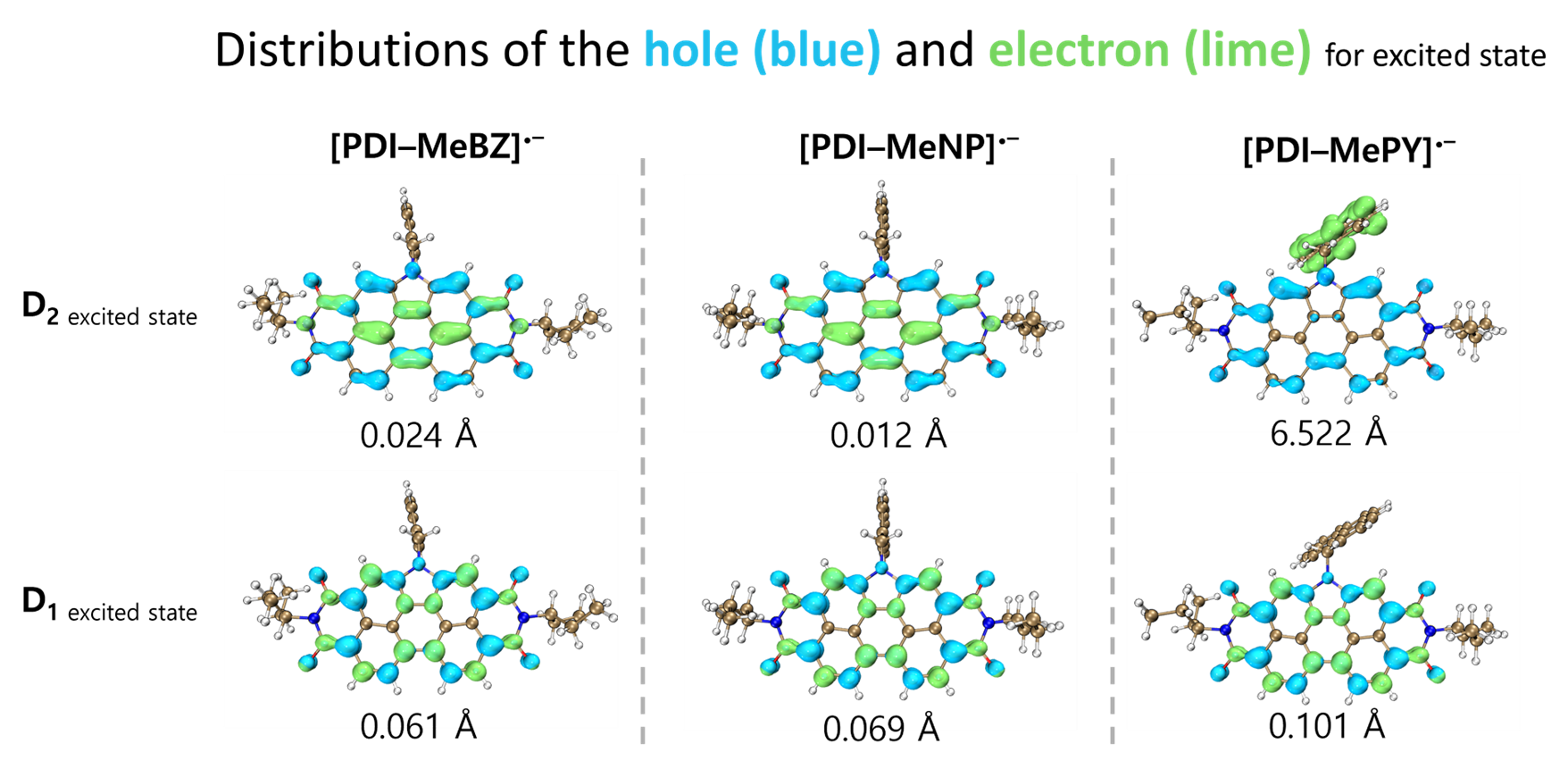
** Figure S33. Excited state hole (blue) and electron (lime) distributions with *D* index values of (A) [**PDI**–**MeBZ**]**^•−^**, (B) [**PDI**–**MeNP**]**^•−^**, and (C) [**PDI–MePY**]**^•−^** for the D_0_ → D_1_ and D_0_ → D_2_ transitions.

**(C)**

**(B)**

**(A)**


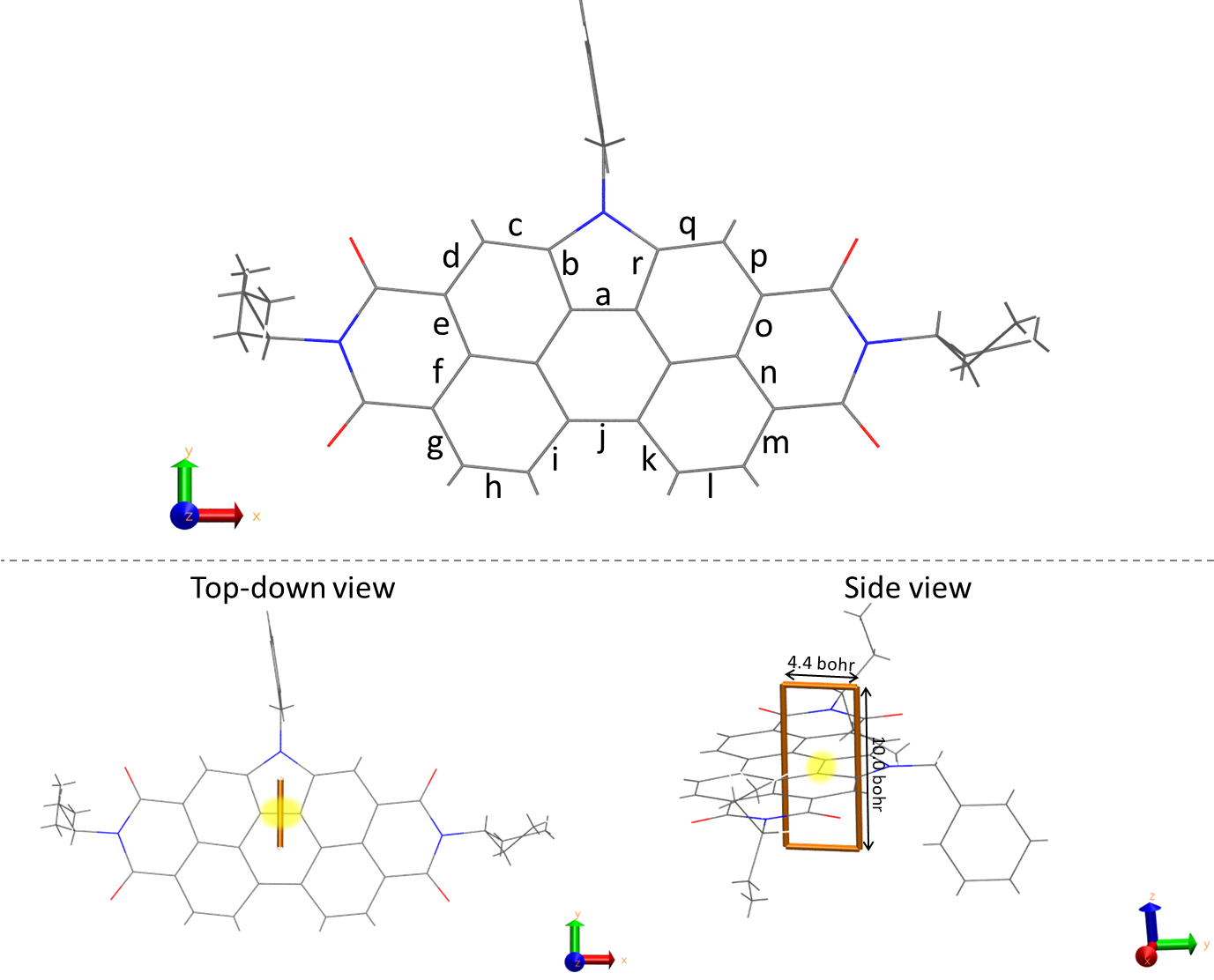


**(A)**

**(B)**

Figure S34. Current strength susceptibilities and pathways for [**PDI**–**MeR**]**^•−^** (B3LYP/6-31G(d)). The bond current strength were calculated by numerical integration of the current flow passing through cut planes (4.4 × 10.0 bohr) perpendicularly to the selected bonds. The selected bonds were integrated counterclockwise with the magnetic field oriented along +Z: negative and positive values correspond to clockwise (diatropic) and counterclockwise (paratropic) currents, respectively. (A) Selected bonds (a – r), (B) Representative cut plane perpendicularly to the bond a (top-down and side views).

Table S18. The induced current strengths (nA / T) of selected bonds (a – r) in [**PDI**–**MeBZ**]**^•−^** at the B3LYP/6-31G(d) level.

| Bond | a | b | c | d | e | f |
| --- | --- | --- | --- | --- | --- | --- |
| Induced current | 1.3 | -0.3 | -8.5 | -9.0 | -6.1 | -5.6 |
| Bond | g | h | i | j | k | l |
| Induced current | -8.4 | 8.4 | 8.6 | 9.0 | -8.7 | -8.4 |
| Bond | m | n | o | p | q | r |
| Induced current | 8.4 | 5.7 | 6.1 | 8.9 | 8.4 | 0.4 |

Table S19. The induced current strengths (nA / T) of selected bonds (a – r) in [**PDI**–**MeNP**]**^•−^** at the B3LYP/6-31G(d) level.

| Bond | a | b | c | d | e | f |
| --- | --- | --- | --- | --- | --- | --- |
| Induced current | -1.3 | -0.4 | 8.4 | -8.9 | -6.1 | -5.7 |
| Bond | g | h | i | j | k | l |
| Induced current | -8.5 | -8.4 | 8.6 | -9.0 | -8.6 | -8.4 |
| Bond | m | n | o | p | q | r |
| Induced current | 8.4 | 5.7 | 6.1 | 8.9 | 8.4 | 0.4 |

Table S20. The induced current strengths (nA / T) of selected bonds (a – r) in [**PDI**–**MePY**]**^•−^** at the B3LYP/6-31G(d) level.

| Bond | a | b | c | d | e | f |
| --- | --- | --- | --- | --- | --- | --- |
| Induced current | -1.2 | -0.4 | 7.8 | -8.7 | -5.9 | -5.6 |
| Bond | g | h | i | j | k | l |
| Induced current | -8.4 | -8.3 | 8.5 | -9.0 | -8.7 | -8.5 |
| Bond | m | n | o | p | q | r |
| Induced current | 8.5 | 5.8 | 6.2 | 9.0 | 8.6 | 0.2 |

**References**

[1] R. El-Berjawi, P. Hudhomme, “Synthesis of a perylenediimide-fullerene C_60_ dyad: A simple use of a nitro leaving group for a Suzuki-Miyaura coupling reaction”, *Dyes and Pigments* 159 (2018): 551-556, https://doi.org/10.1016/j.dyepig.2018.07.037

[2] R. K. Gupta, S. K. Pathak, B. Pradhan, et al., “Self-assembly of luminescent *N*-annulated perylene tetraesters into fluid columnar phases”, *Soft Matter* 11 (2015): 3629-3636, https://doi.org/10.1039/C5SM00463B

[3] H. Borchert 2014, in *Solar Cells Based on Colloidal Nanocrystals*. Switzerland: Springer Cham.

[4] K. J. Lee, N. Elgrishi, B. Kandemir, J. L. Dempsey, “Electrochemical and spectroscopic methods for evaluating molecular electrocatalysts”, *Nature Reviews Chemistry* 1 (2017): 0039, https://doi.org/10.1038/s41570-017-0039

[5] X. Li, K. Perera, J. He, A. Gumyusenge, J. Mei, “Solution-processable electrochromic materials and devices: roadblocks and strategies towards large-scale applications”, *Journal of Materials Chemistry C* 7 (2019): 12761-12789, https://doi.org/10.1039/C9TC02861G

[6] Y. Zhuang, M. Zhu, Q. Zhang, et al., “Electrochromic/Electrofluorochromic Devices based on Terpyridine‐Attached Viologen Derivatives”, *Advanced Materials Technologies* 9 (2024): 2302080, https://doi.org/10.1002/admt.202302080

[7] H. Schlegel, G. Scuseria, M. Robb, et al., “Gaussian 16, revision C. 01”, *Gaussian Inc.: Wallingford, CT, USA* (2016):

[8] Z. Liu, T. Lu, Q. Chen, “An sp-hybridized all-carboatomic ring, cyclo [18] carbon: Electronic structure, electronic spectrum, and optical nonlinearity”, *Carbon* 165 (2020): 461-467, https://doi.org/10.1016/j.carbon.2020.05.023

[9] T. Lu, F. Chen, “Multiwfn: A multifunctional wavefunction analyzer”, *Journal of computational chemistry* 33 (2012): 580-592, https://doi.org/10.1002/jcc.22885

[10] W. Humphrey, A. Dalke, K. Schulten, “VMD: visual molecular dynamics”, *Journal of molecular graphics* 14 (1996): 33-38, https://doi.org/10.1016/0263-7855(96)00018-5

[11] D. Geuenich, K. Hess, F. Köhler, R. Herges, “Anisotropy of the induced current density (ACID), a general method to quantify and visualize electronic delocalization”, *Chemical Reviews* 105 (2005): 3758-3772, https://doi.org/10.1021/cr0300901

[12] J. Jusélius, D. Sundholm, J. Gauss, “Calculation of current densities using gauge-including atomic orbitals”, *The Journal of chemical physics* 121 (2004): 3952-3963, https://doi.org/10.1063/1.1773136

[13] Povray, Persistence of vision raytracer, https://www.povray.org/, accessed: 4, September.

[14] U. Ayachit 2015, in *The paraview guide: a parallel visualization application*. Clifton Park, NY, USA: Kitware, Inc. 1930934300.
